# Supplementary material for: A unified strategy toward total syntheses of lindenane sesquiterpenoid [4 + 2] dimers
Source: Nat Commun. 2019 Apr 23;10:1892. doi: 10.1038/s41467-019-09858-8 (PMC6478710; doi:10.1038/s41467-019-09858-8)
Supplement: Supplementary file 1 — Supplementary Information [file 41467_2019_9858_MOESM1_ESM.pdf]

Supplementary Information

for

A Unified Strategy toward Total Syntheses of Lindenane Sesquiterpenoid [4+2] Dimers

Du *et al.*

Supplementary Methods

General Information

All reactions were performed under an argon atmosphere with dry solvents under anhydrous conditions, unless otherwise stated. DCM, DIPA, DIPEA, HMDS, MeCN, DMF, Et_3N and toluene were distilled from calcium hydride under argon; MeOH was distilled from dry magnesium turnings and iodine under argon; THF was distilled from sodium-benzophenone under argon; Ac_2O was distilled from K_2CO_3 after being preprocessed by P_2O_5. Unless otherwise noted, all the other chemicals were purchased commercially and used without further purification. Flash chromatography was performed using silica gel (200-300 mesh). Thin layer chromatography (TLC) was used for monitoring reactions and visualized by a UV lamp (254 nm and 365 nm), I_2 and developing the plates with *p*-anisaldehyde or phosphomolybdic acid. ^{1}H and ^{13}C NMR were recorded on Bruker DRX-400 MHz NMR spectrometer or Bruker 800 MHz NMR spectrometer with TMS as the internal standard and were calibrated using residual undeuterated solvent as an internal reference ($CDCl_3$: ^{1}H NMR = 7.26, ^{13}C NMR = 77.16; C_6D_6: ^{1}H NMR = 7.16, ^{13}C NMR = 128.06; CD_3COCD_3: ^{1}H NMR = 2.05, ^{13}C NMR = 29.84; CD_3OD: ^{1}H NMR = 3.31 and 4.87, ^{13}C NMR = 49.00; Pyridine-d^5: ^{1}H NMR = 8.74, 7.58 and 7.22). Abbreviations in ^{1}H NMR data are illustrated as follows: s = singlet, d = doublet, t = triplet, dd = doublet of doublet, ddd = doublet of doublet of doublet, dt = doublet of triplet, td = triplet of doublet, m = multiplet, br = broad. Coupling constants (*J*) are reported in Hertz (Hz). Optical rotations were measured at the sodium D line with a 100 mm path length cell, and are reported as follows: $[\alpha]_D^T$,

concentration (g/100 mL), and solvent. High resolution mass spectra (HRMS) were recorded by using Bruker-FT-MS spectrometers. Infrared (IR) spectra were recorded on a NEXUS 670 FT-IR device and are reported in wavenumbers (cm^{-1}).

Experimental Procedures

Procedure for the Preparation of Compound 19 (known procedure[1,2])

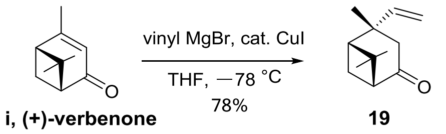

Supplementary Figure 1. Synthesis of compound **19**

In each pot (4 pots in total), to a suspension of CuI (38.1 g, 200 mmol) in THF (380 mL) at -78 °C was added vinyl magnesium bromide (1.0 M in THF, 800 mL, 800 mmol). The solution was stirred for 50 min at this temperature. A solution of (+)-verbenone (60.1 g, 400 mmol) in THF (380 mL) was added dropwise to this solution and the reaction mixture was stirred at -78 °C for 30 min. A mixture of water (30.0 mL) and THF (300 mL) was added to every pot at -78 °C to quench the reaction. Then the mixture of each pot was combined and poured to water (2.0 L) at rt. Then the mixture was filtered through a pad of silica gel and washed with EtOAc. The layers were separated and the aqueous layer was extracted with EtOAc (6 × 2.0 L). Then the combined organic layers were dried over Na_2SO_4, filtered and concentrated under reduced pressure. The residue was purified by flash column chromatography on silica gel (20:1 petroleum ether-EtOAc) to provide compound **19** (217 g, 78%) as a yellow oil.

Procedure for the Preparation of Compound 20

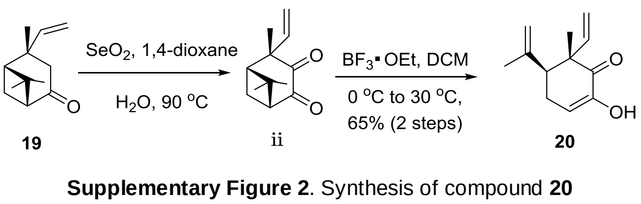

Supplementary Figure 2. Synthesis of compound **20**

Step Ⅰ:

To a solution of compound **19** (8.0 g, 44.87 mmol) in 1,4-dioxane (409 mL) and H_2O (40.9 mL) was added SeO_2 (24.905 g, 224.45 mmol) at room temperature. The solution was allowed to stir at 90 °C for 90 h, and then the solution of $Na_2S_2O_3$ (71 g) in water (600 mL) was added to the reaction at 0 °C. After the mixture was filtered through a pad of silica gel and washed with EtOAc, the filtrate was separated and the aqueous layer was extracted with EtOAc (4 × 300 mL). The combined organic layers were dried over Na_2SO_4, filtered and concentrated under reduced pressure to get the crude product **ii** as a yellow oil. The crude compound **ii** was used for next step without further purification.

Step Ⅱ:

BF_3•Et_2O (3.82 g, 3.32 mL, 26.91 mmol) was added dropwise to a solution of above **ii** in dry DCM (440.0 mL) at 0 °C. Then the reaction was allowed to warm to 30 °C and stirred overnight, before a solution of $NaHCO_3$ (72.0 g) in H_2O (200 mL) was added at 0 °C. The layers were separated and the aqueous layer was extracted with DCM (4 × 300 mL). The combined organic layers were dried over Na_2SO_4, filtered and concentrated under reduced pressure. The residue was purified by flash column chromatography on silica gel (12:1 petroleum ether-EtOAc) to furnish compound **20** (5.596 g, 65 % over two steps) as a colorless oil.

$[\alpha]_D^{22}$ – 35.1 (*c* 0.74, $CHCl_3$);

IR (thin film) 3447, 2979, 2920, 1722, 1680, 1644, 1387, 1220, 1083, 1045, 901, 825,

750 cm $^{-1}$;

^{1}H NMR (400 MHz, $CDCl_3$) δ 6.01 (t, J = 4.8 Hz, 1H), 5.98 (s, 1H), 5.87 (dd, J = 17.5, 10.7 Hz, 1H), 5.18 (d, J = 10.7 Hz, 1H), 5.04 (d, J = 17.5 Hz, 1H), 4.85 (t, J = 1.6 Hz, 1H), 4.75 (s, 1H), 2.72 (t, J = 5.2 Hz, 1H), 2.64 (dt, J = 19.0, 4.8 Hz, A of AB, 1H), 2.38 (dt, J = 19.0, 5.2 Hz, B of AB, 1H), 1.66 (s, 3H), 1.20 (s, 3H);

^{13}C NMR (100 MHz, $CDCl_3$) δ 198.3, 145.9, 145.2, 140.7, 115.4, 114.8, 52.1, 50.8, 26.6, 23.7, 18.0;

HRMS (ESI$^+$) m/z calc'd for $C_{12}H_{17}O_2$ $[M+H]^+$: 193.1223, found 193.1223.

Procedure for the Preparation of Compound 22

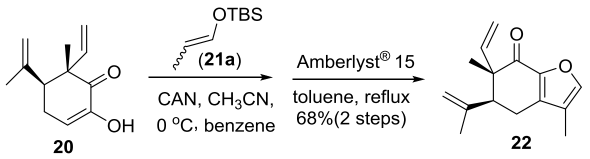

Supplementary Figure 3. Synthesis of compound **22**

Known compound **21a** can be prepared according to the known procedure [4].

Step Ⅰ:

Compound **20** (2.0 g, 10.40 mmol) and **21a** (35.9 g, 208.3 mmol) were dissolved in dry CH_3CN (156 mL) and dry Benzene (52 mL), Then the solution of CAN (14.27 g, 26.03 mmol) in dry CH_3CN (52 mL) was added to the above solution during 15 min at 0 °C. The reaction was immediately quenched with saturated aqueous $Na_2S_2O_3$ solution (50 mL) right after completion of the CAN addition. The layers were separated and the aqueous layer was extracted with EtOAc (3 × 50 mL). The combined organic layers

were dried over Na_2SO_4, filtered and concentrated under reduced pressure to furnish crude compound as a palepale yellow oil.

Step Ⅱ:

The above crude compound was dissolved into toluene (100 mL), and the resin (Amberlyst®15, 3.140 g) was added. The reaction was filtered after refluxing for 8 h, and the filtrate was concentrated under reduced pressure. The residue was purified by flash column chromatography on silica gel (5:1 petroleum ether-EtOAc) to furnish compound **22** (1.630g, 68 % for 2 steps) as a pale yellow oil.

$[\alpha]_D^{22}$ – 213.9 (*c* 0.74, $CHCl_3$);

IR (thin film) 3082, 2974, 2928, 1675, 1640, 1537, 1415, 1374, 1309, 1252, 1158, 1120, 1020, 993, 950, 793, 600 cm^{-1};

^1H NMR (400 MHz, $CDCl_3$) δ 7.37 (d, *J* = 1.4 Hz, 1H), 5.91 (dd, *J* = 17.5, 10.7 Hz, 1H), 5.14 (d, *J* = 10.7 Hz, 1H), 5.08 (d, *J* = 17.5 Hz, 1H), 4.83 (s, 1H), 4.76 (s, 1H), 2.93 (dd, *J* = 17.0, 5.6 Hz, A of AB, 1H), 2.86 (t, *J* = 4.8, 1H), 2.63 (dd, B of AB, *J* = 17.0, 4.8 Hz, 1H), 1.98 (d, *J* = 1.2 Hz, 3H), 1.55 (s, 3H), 1.24 (s, 3H);

^{13}C NMR (100 MHz, $CDCl_3$) δ 188.5, 147.1, 145.6, 145.0, 141.2, 137.5, 120.7, 115.3, 114.7, 53.8, 52.4, 24.5, 23.3, 18.4, 7.9;

HRMS (ESI^+) m/z calc'd for $C_{15}H_{18}O_2Na$ $[M+Na]^+$: 253.1199, found 253.1198.

Procedure for the Preparation of Compound 23

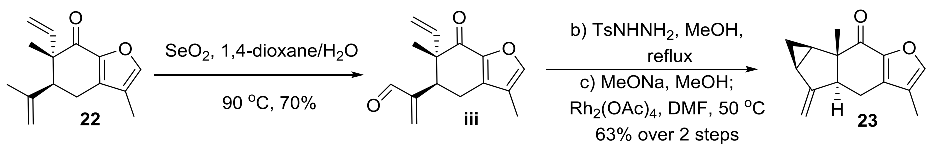

Supplementary Figure 4. Synthesis of compound **23**

Step Ⅰ:

Compound **22** (1.73 g, 7.51 mmol) and SeO_2 (834.2 mg, 7.52 mmol) were dissolved in 1,4-dioxane (130 mL) and H_2O (26 mL). Then the reaction was allowed to stir at 90 °C, and additional SeO_2 (834.2 g, 7.52 mmol) was added per hour. After five-time addition (5.005 g SeO_2 in total), the reaction was allowed to stir at 90 °C for next 3 h. Then the reaction was cooled to room temperature and quenched with saturated aqueous $Na_2S_2O_3$ solution (80 mL). The resulting mixture was extracted with EtOAc (5 × 100 mL). The combined organic layers were dried over Na_2SO_4, filtered and concentrated under reduced pressure. The residue was purified by flash column chromatography on silica gel (5:1 petroleum ether-EtOAc) to furnish compound **iii** (1.284 g, 70 %) as a pale yellow oil.

$[\alpha]_D^{22}$ – 96.1 (*c* 0.43, $CHCl_3$);

IR (thin film) 2980, 2929, 1766, 1672, 1536, 1459, 1416, 1370, 1208, 1021, 996, 953, 881, 750, 668, 597 cm^{-1};

^1H NMR (400 MHz, $CDCl_3$) δ 9.43 (s, 1H), 7.40 (q, *J* =1.2, 1H), 6.22 (s, 1H), 6.10 (s, 1H), 5.88 (dd, *J* = 17.5, 10.7 Hz, 1H), 5.14 (d, *J* = 10.8 Hz, 1H), 5.01 (d, *J* = 17.5 Hz, 1H), 3.66(t, *J* = 6.0, 1H), 2.84 (dd, *J* = 17.2, 5.2 Hz, A of AB,1H), 2.68 (dd, B of AB, *J* = 17.2, 6.9 Hz, 1H), 1.96 (d, *J* =1.2, 3H), 1.18 (s, 3H);

^{13}C NMR (100 MHz, $CDCl_3$) δ 194.2, 188.1, 149.3, 146.8, 145.4, 140.1, 137.2, 136.0,

121.1, 115.9, 52.4, 41.3, 24.2, 17.1, 7.8;

HRMS (ESI+) m/z calc'd for $C_{15}H_{17}O_3$ $[M+H]^+$: 245.1172, found 245.1177.

Step Ⅱ:

To a solution of compound **iii** (2.62 g, 10.72 mmol) in absolute MeOH (107 mL) was added *p*$TsNHNH_2$ (5.09 g, 27.33 mmol) at room temperature. The solution was allowed to reflux for 0.5 h, and then the reaction was cooled to room temperature and concentrated under reduced pressure. The residue was dissolved in EtOH (200 mL) again, and the ethanol solvent was removed under reduced pressure to completely remove water in the crude mixture, affording a pale yellow solid without further purification.

Step Ⅲ:

To a solution of the above crude compound in absolute methanol (150 mL) at 0 °C was added NaOMe (24.81 mL, 1.0 M in MeOH). After stirring for 10 min at 0 °C, the solution was allowed to stir at 30 °C for additional 30 min. The methanol was removed under reduced pressure to afford tosyl hydrazone sodium salt as a pale yellow solid without further purification. A mixture of this tosyl hydrazone sodium salt and $Rh_2(OAc)_4$ (2.417 g, 5.36 mmol) in dry DMF (630 mL) was stirred for 4 h at 50 °C. After removal of the solvent by vacuum distillation, the residue was purified by flash column chromatography on silica gel (10:1 petroleum ether-EtOAc) to provide compound **23** (1.537 g, 63% for 2 steps) as a white solid.

$[\alpha]_D^{22}$ – 16.1 (*c* 0.30, $CHCl_3$);

IR (thin film) 2924, 2853, 1767, 1684, 1457, 1409, 1340, 1261,1020, 976, 951, 886, 798, 650, 575 cm $^{-1}$;

^{1}H NMR (400 MHz, $CDCl_3$) δ 7.36 (q, *J* = 1.2 Hz, 1H), 5.08 (dt, *J* = 2.4, 0.9 Hz, 1H), 4.85 (t, *J* = 2.4 Hz, 1H), 3.39 – 3.29 (m, 1H), 2.60 (dd, A of AB, *J* = 16.8, 3.6 Hz, 1H), 2.30 (dd, *J* = 16.8, 11.2 Hz, B of AB, 1H), 2.21 (ddd, *J* = 8.2, 7.3, 3.6 Hz, 1H), 2.00 (s, 3H), 1.98 – 1.92 (m, 1H), 0.97 (td, *J* = 8.4, 5.6 Hz, 1H), 0.90 (s, 3H), 0.81 (dt, *J* = 5.6, 3.6 Hz, 1H);

^{13}C NMR (100 MHz, $CDCl_3$) δ 191.9, 150.5, 147.7, 144.5, 138.1, 121.1, 107.4, 62.4, 50.4, 22.6, 22.2, 20.3, 17.1, 16.5, 8.1;

HRMS (ESI$^+$) m/z calc'd for $C_{15}H_{17}O_2$ $[M+H]^+$: 229.1223, found 229.1224.

Procedure for the Preparation of Compound 6

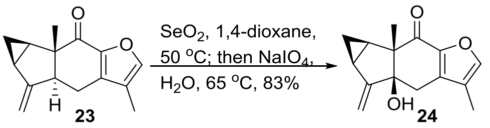

Supplementary Figure 5. Synthesis of compound **24**

To a solution of compound **23** (1.10 g, 4.818 mmol) in 1,4-dioxane (125 mL) was added SeO_2 (588.1 mg, 5.30 mmol) at room temperature. The solution was allowed to stir at 50 °C for 55 min, and then the reaction was cooled to room temperature. A solution of $NaIO_4$ (5.15 g, 24.1 mmol) in H_2O (12.5 mL) were added, and the resultant solution was allowed to stir at 65 °C for additional 1 h. Then the reaction was cooled to room temperature and quenched with saturated aqueous $Na_2S_2O_3$ solution (20 mL). The 1,4-

dioxane was removed under reduced pressure, and the aqueous layer was extracted with EtOAc (5 × 20 mL). The combined organic layers were dried over Na_2SO_4, filtered and concentrated under reduced pressure. The residue was purified by flash column chromatography on silica gel (4:1 petroleum ether-EtOAc) to furnish compound **24** (977.6 mg, 83 %) as a pale yellow solid.

$[\alpha]_D^{22}$ – 10.4 (*c* 0.41, $CHCl_3$);

IR (thin film) 3439, 2927, 2326, 1772, 1710, 1650, 1540, 1456, 1423, 1380, 1046, 949, 816, 762, 685 cm $^{-1}$;

^{1}H NMR (400 MHz, $CDCl_3$) δ 7.39 (s, 1H), 5.00 (s, 1H), 4.95 (s, 1H), 3.15 (d, *J* = 12.6 Hz, A of AB, 1H), 2.80 (d, *J* = 12.6 Hz, B of AB, 1H), 2.25 (ddd, *J* = 8.5, 6.1, 4.3 Hz, 1H), 2.00 (d, *J* = 1.1 Hz, 3H), 1.84 (ddd, *J* = 9.0, 6.1, 3.3 Hz, 1H), 1.40 – 1.37 (m, 1H), 1.37 (s, 3H), 0.77 (td, *J* = 8.5, 4.7 Hz, 1H);

^{13}C NMR (100 MHz, $CDCl_3$) δ 188.5, 154.3, 146.5, 146.3, 136.7, 120.5, 105.2, 80.1, 58.5, 28.8, 26.5, 22.4, 16.1, 12.8, 7.8;

HRMS (ESI$^+$) m/z calc'd for $C_{15}H_{17}O_3$ $[M+H]^+$: 245.1172, found 245.1176.

Procedure for the Preparation of Compound iv

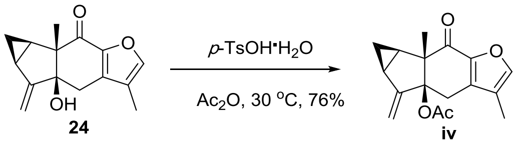

Supplementary Figure 6. Synthesis of compound **iv**

p-TsOH•H_2O (2.42 g, 12.7 mmol) was added to a solution of **24** (778.9 mg, 3.19 mmol) in Ac_2O (34 mL) at 0 °C. After the reaction was allowed to warm to 30 °C and stir for

2.5 h, it was added dropwise to a solution of $NaHCO_3$ (97.0 g) in H_2O (150 mL) at 0 °C. Stirring continued until the bubbling of CO_2 ceased. The mixture was extracted with EtOAc (3 × 100 mL). The combined organic layers were dried over Na_2SO_4, filtered and concentrated under reduced pressure. The residue was purified by flash column chromatography on silica gel (6:1 petroleum ether-EtOAc) to furnish **iv** (694.0 mg, 76 %) as a pale yellow solid.

$[\alpha]_D^{22}$ – 137.3 (*c* 0.99, $CHCl_3$);

IR (thin film) 3122, 2931, 1739, 1669, 1543, 1458, 1422, 1369, 1351, 1307, 1240, 1180, 1057, 1025, 996, 972, 945, 840,775, 600, 566 cm $^{-1}$;

^1H NMR (400 MHz, $CDCl_3$) δ 7.40 (s, 1H), 5.27 (s, 1H), 5.22 (s, 1H), 4.31 (d, *J* = 12.6 Hz, A of AB, 1H), 2.75 (d, B of AB, *J* = 12.6 Hz, 1H), 2.30 (ddd, *J* = 8.5, 6.3, 4.3 Hz, 1H), 2.00 (s, 3H), 1.99 (s, 3H), 1.85 (ddd, *J* = 9.0, 6.2, 3.2 Hz, 1H), 1.40 (s, 3H), 1.04 (q, *J* = 4.4 Hz, 1H), 0.74 (td, *J* = 8.4, 5.0 Hz, 1H);

^{13}C NMR (100 MHz, $CDCl_3$) δ 187.0, 169.1, 146.6, 146.2, 146.0, 136.1, 120.9, 111.9, 87.7, 59.3, 25.0, 24.8, 22.7, 22.4, 17.2, 12.0, 7.8;

HRMS (ESI$^+$) m/z calc'd for $C_{17}H_{19}O_4$ $[M+H]^+$: 287.1278, found 287.1286.

Procedure for the Preparation of Compound v and vi

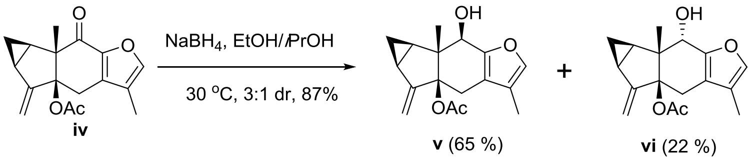

Supplementary Figure 7. Synthesis of compound **v** and **vi**

$NaBH_4$ (519.9 mg, 13.743 mmol) was added to a solution of **iv** (787.8 mg, 2.75 mmol)

in EtOH (37.5 mL) and *i*PrOH (37.5 mL) at 0 °C. Then the reaction was allowed to warm to 30 °C and stir for 10 h, before extra $NaBH_4$ (519.9 mg, 13.74 mmol) was added to the solution. After stirring at 30 °C for another 10 h, the reaction was quenched with H_2O (30 mL) at 0 °C. The EtOH and *i*PrOH were removed under reduced pressure, and the aqueous layer was extracted with EtOAc (5 × 40 mL). The combined organic layers were dried over Na_2SO_4, filtered and concentrated under reduced pressure. The residue was purified by flash column chromatography on silica gel (8:1 petroleum ether-EtOAc) to furnish compound **v** (513.3 mg, 65 %) as a colorless oil and compound **vi** (174.3 mg, 22 %) as a colorless oil.

Compound **v**:

$[\alpha]_D^{22}$ –184.0 (*c* 0.76, $CHCl_3$);

IR (thin film) 3490, 2920, 1740, 1654, 1369, 1241, 1183, 1098, 1013, 986, 968, 896, 814, 760, 668 cm^{-1};

^1H NMR (400 MHz, $CDCl_3$) δ 7.09 (s, 1H), 5.29 (s, 1H), 5.25 (s, 1H), 4.50 (s, 1H), 3.83 (d, *J* = 12.6 Hz, A of AB, 1H), 2.51 (d, *J* = 12.6 Hz, B of AB, 1H), 2.37 (br, 1H), 1.98 (s, 3H), 1.90 (s, 3H), 1.87 – 1.85 (m, 2H), 1.29 – 1.25 (m, 1H), 1.17 (s, 3H), 0.73 (td, *J* = 8.3, 5.1 Hz, 1H);

^{13}C NMR (100 MHz, $CDCl_3$) δ 169.2, 147.9, 147.1, 139.4, 119.4, 115.6, 111.7, 88.1, 72.0, 52.0, 27.6, 26.5, 22.7, 20.8, 14.3, 11.6, 8.1;

HRMS (ESI^+) m/z calc'd for $C_{17}H_{20}O_4Na$ $[M+Na]^+$: 311.1254, found 311.1252.

Compound **vi**:

$[\alpha]_D^{22}$ – 192.9 (*c* 0.35, $CHCl_3$);

IR (thin film) 3475, 2980, 2915, 2820, 2326, 1740, 1686, 1610, 1493, 1388, 1350, 1090, 985, 917, 820, 807, 881, 830, 810 cm^{-1};

^{1}H NMR (400 MHz, $CDCl_3$) δ 7.13 (s, 1H), 5.36 (s, 1H), 5.33 (s, 1H), 4.37 (d, *J* = 8.4 Hz, 1H), 3.97 (d, *J* = 12.0 Hz, A of AB, 1H), 2.51 (d, *J* = 12.0 Hz, B of AB, 1H), 2.06–2.02 (m, 1H), 1.97 (s, 3H), 1.92 (s, 3H), 1.74 – 1.71 (m, 1H), 1.52 (d, *J* = 9.9 Hz, 1H), 1.32 (dq, *J* = 5.1, 3.3, 2.0 Hz, 1H), 1.21 (s, 3H), 0.75 (td, *J* = 8.2, 4.9 Hz, 1H);

^{13}C NMR (100 MHz, $CDCl_3$) δ 168.8, 148.9, 148.8, 140.1, 119.1, 116.6, 110.5, 86.3, 71.8, 51.7, 26.9, 26.4, 23.1, 22.7, 18.6, 12.7, 8.1;

HRMS (ESI$^+$) m/z calc'd for $C_{17}H_{20}O_4Na$ [M+Na]$^+$: 311.1254, found 311.1255.

Procedure for the Preparation of Compound iv from vi

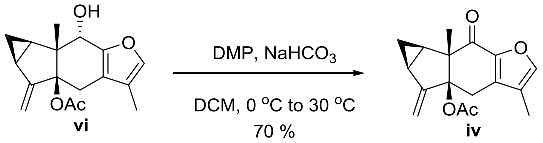

Supplementary Figure 8. Synthesis of compound **iv** from **vi**

$NaHCO_3$ (147.4 mg, 1.75 mmol) and Dess-Martin periodinane (371.5 mg, 0.876 mmol) were sequentially added to a solution of **vi** (126.3 mg, 0.438 mmol) in DCM (10 mL) at 0 °C. Then the reaction was allowed to stir at 30 °C for 4 h, before it was quenched with saturated solution of $Na_2S_2O_3$ (3 mL) at 0 °C. Then H_2O (5 mL) was added to the mixture. The layers were separated and the aqueous layer was extracted with DCM (5 × 8 mL). The combined organic layers were dried over Na_2SO_4, filtered and concentrated under reduced pressure. The residue was purified by flash column

chromatography on silica gel (6:1 petroleum ether-EtOAc) to furnish compound **iv** (87.7 mg, 70%) as a pale yellow solid.

Procedure for the Preparation of Compound 25

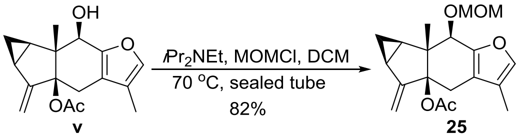

Supplementary Figure 9. Synthesis of compound **25**

To a solution of compound **v** (438.9 mg, 1.523 mmol) in DCM (20.7 mL) were added DIPEA (1.97 g, 2.5 mL, 15.24 mmol) and MOMCl (613.49 mg, 0.58 mL, 7.62 mmol) in order at 0 °C. Then the reaction was heated at 70 °C in a sealed tube for 10 h, before it was quenched with saturated aqueous $NaHCO_3$ solution (40.0 mL) at 0 °C. The layers were separated and the aqueous layer was extracted with DCM (5 × 20.0 mL). The combined organic layers were dried over Na_2SO_4, filtered and concentrated under reduced pressure. The residue was purified by flash column chromatography on silica gel (10:1 petroleum ether-EtOAc) to furnish compound **25** (413.3 mg, 82%) as a colorless oil.

$[\alpha]_D^{22}$ – 95.4 (*c* 0.52, $CHCl_3$);

IR (thin film) 2930, 2326, 1743, 1656, 1367, 1307, 1286, 1243, 1190, 1152, 1101, 1025, 969, 919, 897, 808, 760, 666, 597 cm^{-1};

^1H NMR (400 MHz, $CDCl_3$) δ 7.07 (s, 1H), 5.30 (s, 1H), 5.25 (s, 1H), 5.07 (d, *J* = 6.9 Hz, A of AB, 1H), 4.87 (d, *J* = 6.9 Hz, B of AB, 1H), 4.41 (s, 1H), 3.88 (d, *J* = 17.4 Hz, 1H), 3.51 (s, 3H), 2.49 (dd, *J* = 17.3, 2.6 Hz, 1H), 1.97 (s, 3H), 1.95 – 1.84 (m, 2H),

1.90 (s, 3H), 1.30 (td, $J = 4.6, 3.2$ Hz, 1H), 1.24 (s, 3H), 0.72 (td, $J = 8.3, 5.1$ Hz, 1H);

^{13}C NMR (100 MHz, $CDCl_3$) δ 169.0, 147.6, 146.5, 139.3, 119.1, 115.7, 111.9, 98.0, 88.0, 77.8, 56.3, 51.9, 27.7, 25.8, 22.8, 21.2, 14.1, 12.2, 8.1;

HRMS (ESI^+) m/z calc'd for $C_{19}H_{24}O_5Na$ $[M+Na]^+$: 355.1516, found 355.1510.

Procedure for the Preparation of Compound 32

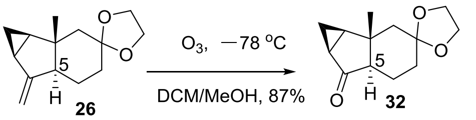

Supplementary Figure 10. Synthesis of compound **32**

Known compound **26** can be prepared in a ten-gram scale over six steps from commercially available (+)-verbenone according to our reported procedure [1-3].

Ozone was generated in the usual fashion by a laboratory ozonator using commercial-grade oxygen (gas flow rate: 5 L / min) as a source. The ozonolysis was carried out in ordinary round-bottomed flasks at -78 °C. Ozone (ozone concentration: 29.8 mg/L) was introduced through a glass tube extended very close to the magnetic stirring bar.

Compound **26** (1.50 g, 6.81 mmol) was dissolved in MeOH (60 mL) and DCM (60 mL), and ozone was passed through the solution at 78 °C for 25 min. Then ozone generation was stopped, but the oxygen was still passed through the solution until there was no peroxide detectable by potassium iodide starch test paper. After removal of the solvent until about 4 mL solution remained, the residue was immediately transferred to the top of a column and purified by flash column chromatography on silica gel (10:1 petroleum ether-EtOAc) to provide compound **32** (1.320 g, 87 %) as a colorless oil.

***Causion**: normal treatment with dimethyl sulfide or triphenyl phosphine resulted in*

*serious epimerization at C5 to afford compound **33**, instead of **32**, as the major or sole product. So this workup procedure must be avoided.*

[α] $_D^{22}$ + 7.3 (*c* 0.40, MeOH);

IR (thin film) 2960, 2950, 2926, 1722, 1488, 1476, 1345, 1322, 1261, 1205, 1094, 1032, 857, 802, 501 cm^{-1};

^{1}H NMR (400 MHz, CD_3OD) δ 3.98 – 3.92 (m, 2H), 3.82 (t, *J* = 6.1 Hz, 2H), 2.20 (dd, *J* = 12.1, 2.2 Hz, 1H), 1.93 – 1.84 (m, 2H), 1.79 (d, *J* = 2.0 Hz, 1H), 1.73 (d, *J* = 13.0 Hz, 1H), 1.56 (td, *J* = 12.9, 4.7 Hz, 1H), 1.48 (td, *J* = 12.7, 12.1, 3.1 Hz, 1H), 1.38 – 1.36 (m, 1H), 1.34 (dd, *J* = 4.1, 1.4 Hz, 1H), 0.96 (s, 3H), 0.95 – 0.91 (m, 1H), 0.75 (td, *J* = 8.7, 5.6 Hz, 1H);

^{13}C NMR (100 MHz, CD_3OD) δ 114.0, 111.1, 67.4, 65.5, 64.4, 50.3, 39.5, 37.5, 30.7, 24.7, 21.3, 18.4, 12.7;

HRMS (ESI$^+$) m/z calc'd for $C_{13}H_{19}O_3$ [M+H]$^+$: 233.1329, found 233.1328.

Procedure for the Preparation of Compound 34

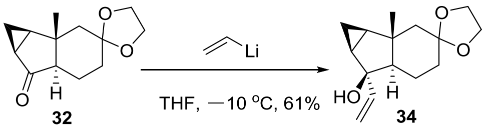

Supplementary Figure 11. Synthesis of compound **34**

*n*BuLi (2.5 M in hexane, 8.1 mL, 20.24 mmol) was added to a solution of tributyl(vinyl)tin (7.84 g, 24.73 mmol) in THF (50 mL) in every pot (9 pots in total) at -78 °C and the mixture was stirred for 50 min at the same temperature. Then a solution of compound **32** (1.0 g, 4.497 mmol) in THF (6 mL) was added dropwise to the above

solution at -10 °C during 5 min. After stirring for 2 h, H_2O (10 mL) was added to every pot and the resulting mixture of every pot was combined. The layers were separated and the aqueous layer was extracted with EtOAc (3 × 180 mL). The combined organic layers were dried over Na_2SO_4, filtered and concentrated under reduced pressure. The residue was purified by flash column chromatography on silica gel (10:1 petroleum ether-EtOAc) to furnish compound **34** (6.198 g, 61%) as a colorless oil.

$[\alpha]_D^{22}$ + 45.6 (*c* 2.30, $CHCl_3$);

IR (thin film) 3443, 3075, 2996, 2954, 2913, 2875, 1438, 1317, 1302, 1265, 1206, 1189, 1172, 1075, 1027, 1001, 967, 938, 904, 874, 835, 795, 774 cm^{-1};

^1H NMR (400 MHz, $CDCl_3$) δ 5.99 (dd, *J* = 17.2, 10.6 Hz, 1H), 5.24 (d, *J* = 18.3 Hz, 1H), 5.08 (d, *J* = 10.6 Hz, 1H), 3.94 (qt, *J* = 7.9, 3.7 Hz, 2H), 3.86 – 3.78 (m, 2H), 1.94 – 1.81 (m, 2H), 1.73 (d, *J* = 13.2 Hz, 1H), 1.62 (dd, *J* = 11.6, 3.6, 1H), 1.55 (ddd, *J* = 8.9, 7.0, 4.1 Hz, 1H), 1.50 – 1.43 (m, 1H), 1.37 – 1.30 (m, 2H), 1.29 – 1.23 (m, 2H), 1.21 (s, 1H), 1.08 (s, 3H), 0.69 – 0.60 (m, 1H);

^{13}C NMR (100 MHz, $CDCl_3$) δ 145.1, 111.6, 110.3, 80.3, 66.9, 64.6, 63.5, 49.8, 40.3, 36.7, 31.4, 31.3, 18.4, 17.2, 12.3;

HRMS (ESI$^+$) m/z calc'd for $C_{15}H_{23}O_3$ $[M+H]^+$: 251.1642, found 251.1637.

Procedure for the Preparation of Compound 33

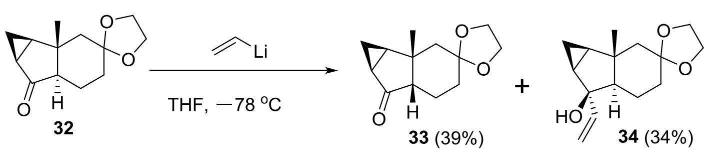

Supplementary Figure 12. Synthesis of compound **33**

*n*BuLi (2.5 M in hexane, 0.162 mL, 0.405 mmol) was added to a solution of

tributyl(vinyl)tin (157.2 mg, 0.496 mmol) in THF (2 mL) at -78 °C and the mixture was stirred for 50 min at the same temperature. Then a solution of compound **32** (20.0 mg, 0.090 mmol) in THF (1 mL) was added dropwise to the above solution at -78 °C during 3 min. After stirring for 0.5 h, H_2O (2 mL) was added to quench the reaction. The layers were separated and the aqueous layer was extracted with EtOAc (3 × 3 mL). The combined organic layers were dried over Na_2SO_4, filtered and concentrated under reduced pressure. The residue was purified by flash column chromatography on silica gel (10:1 petroleum ether-EtOAc) to furnish compound **33** (7.7 mg, 39%) as a colorless oil and **34** (7.7mg, 34%) as a colorless oil.

Compound **33**:

$[\alpha]_D^{22}$ – 4.7 (*c* 0.19, $CHCl_3$);

IR (Neat Film) 2955, 2927, 2856, 2326, 1722, 1644, 1382, 1284, 1176, 1093, 1032, 826 cm^{-1};

^1H NMR (400 MHz, CD_3COCD_3) δ 3.97 – 3.88 (m, 2H), 3.87 – 3.80 (m, 2H), 1.91 (ddd, *J* = 14.2, 5.3, 2.2 Hz, 2H), 1.87 – 1.82 (m, 1H), 1.79 (ddd, *J* = 13.9, 2.8, 1.4 Hz, 1H), 1.72 (ddd, *J* = 8.5, 5.4, 3.0 Hz, 1H), 1.65 – 1.55 (m, 1H), 1.49 – 1.43 (m, 1H), 1.43 – 1.39 (m, 1H), 1.30 (s, 3H), 1.26 (td, *J* = 4.8, 2.9 Hz, 1H), 1.20 (dd, *J* = 13.6, 4.0 Hz, 1H), 1.15 – 1.09 (m, 1H);

^{13}C NMR (100 MHz, CD_3OD) δ 216.2, 109.4, 65.5, 64.6, 45.7, 45.4, 40.5, 35.3, 32.1, 27.7, 24.0, 18.1, 14.9;

HRMS (ESI^+) m/z calc'd for $C_{13}H_{18}O_3$ $[M+Na]^+$: 245.1148, found 245.1142.

Procedure for the Preparation of Compound 17

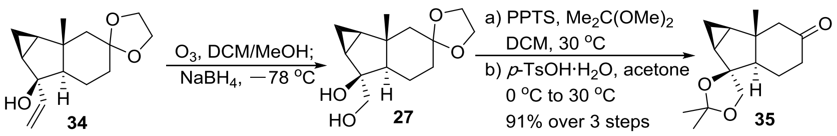

Supplementary Figure 13. Synthesis of compound **35**

Ozone is generated in the usual fashion by a laboratory ozonator using commercial-grade oxygen (gas flow rate: 5.5 L / min) as a source. The ozonolysis was carried out in ordinary round-bottomed flasks. Ozone (ozone concentration: 33.6 mg/L) was introduced through a glass tube extended very close to the magnetic stirring bar.

Step Ⅰ:

In every pot (3 pots in total), ozone was passed through a solution of compound **34** (2.38 g, 9.507 mmol) in MeOH (60 mL) and DCM (60 mL) at -78 °C for 15 min. Then $NaBH_4$ (1.70 g, 44.849 mmol) was added with stirring and the mixture was allowed to stir at -78 °C for 1.5 h. The reaction was quenched with H_2O (15 mL) and the resulting mixture of every pot was combined. After removal of the solvent, the aqueous layer was extracted with EtOAc (4 × 100.0 mL). The combined organic layers were dried over Na_2SO_4, filtered and concentrated under reduced pressure to furnish crude compound **27** as a white solid. The compound **27** was used for next step without further purification.

Step Ⅱ:

The above crude compound **27** was divided into 3 parts equally, and every part of **27**

was dissolved in dry DCM, 2,2-Dimethoxypropane and PPTS (923.8 mg, 3.674 mmol) were added to the solution in order at 0 °C. The mixture was allowed to stir at 30 °C for 8 h, and then the reaction was quenched with saturated solution of $NaHCO_3$ (30 mL) and the resulting mixture of every pot was combined. After removal of the solvent, the aqueous layer was extracted with EtOAc (7 × 100 mL). The combined organic layers were dried over Na_2SO_4, filtered and concentrated under reduced pressure to furnish crude product as a colorless oil. This compound was used for next step without further purification.

Step III:

The above crude compound was divided into 3 parts equally, every part of the compound was dissolved in acetone (105 mL), *p*-TsOH•H_2O (904.3 mg, 4.755 mmol) was added to the solution at 0 °C. After stirring for 40 min at 30 °C, the reaction was quenched with saturated aqueous $NaHCO_3$ solution (20 mL) and the resulting mixture of every pot was combined. After removal of the solvent, the aqueous layer was extracted with EtOAc (5 × 100 mL). The combined organic layers were dried over Na_2SO_4, filtered and concentrated under reduced pressure. The residue was purified by flash column chromatography on silica gel (6:1 petroleum ether-EtOAc) to furnish compound **35** (6.5 g, 91% for 3 steps) as a white solid.

$[\alpha]_D^{22}$ – 7.6 (*c* 2.00, $CHCl_3$);

IR (thin film) 3024, 2940, 2883, 1707, 1465, 1440, 1375, 1368, 1206, 1160, 1105, 1057, 1977, 965, 883,871, 795 cm^{-1};

^{1}H NMR (400 MHz, $CDCl_3$) δ 4.06 (d, J = 8.0 Hz, A of AB, 1H), 3.86 (d, J = 8.1 Hz, B of AB, 1H), 2.49 (d, J = 16.0 Hz, 1H), 2.47 – 2.40 (m, 1H), 2.38 (d, J = 14.4 Hz, 1H), 2.19 – 2.10 (m, 1H), 2.10 – 2.02 (m, 1H), 1.67 – 1.55 (m, 3H), 1.39 (s, 3H), 1.38 – 1.33 (m, 1H), 1.26 (s, 3H), 1.15 (dt, J = 5.5, 4.0 Hz, 1H), 0.76 (s, 3H), 0.71 (td, J = 8.7, 5.6 Hz, 1H);

^{13}C NMR (100 MHz, $CDCl_3$) δ 211.5, 110.0, 86.4, 72.7, 61.4, 56.8, 41.5, 40.0, 30.6, 29.9, 26.7, 26.5, 20.0, 18.6, 12.9;

HRMS (ESI$^+$) m/z calc'd for $C_{15}H_{23}O_3$ $[M+H]^+$: 251.1642, found 251.1639.

Procedure for the Preparation of Compound 36

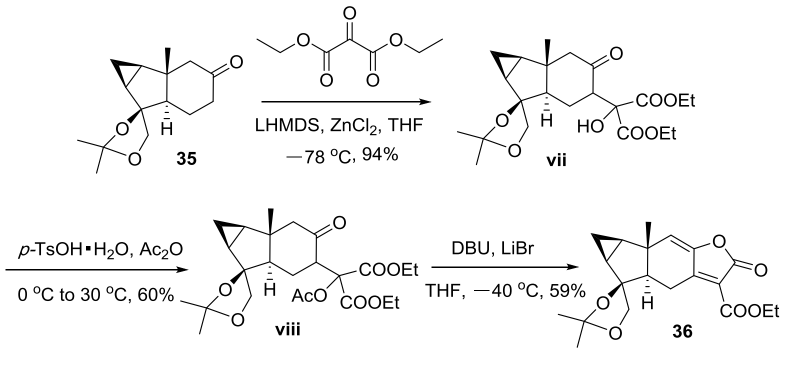

Supplementary Figure 14. Synthesis of compound **36**

Step Ⅰ:

To a solution of LHMDS, prepared from HMDS (6.19 g, 8.0 mL, 38.37 mmol) and *n*BuLi (2.5 M in hexane, 14.37 mL, 35.97 mmol) in THF (1000 mL), was added **35** (3.0 g, 11.98 mmol) in THF (23 mL) at -78 °C and the resulting mixture was stirred for 30 min at the same temperature. The mixture of diethyl ketomalonate (2.92 g, 2.56 mL, 16.79 mmol) and $ZnCl_2$ (2.29 g, 16.79 mmol) in THF (20 mL) was added to the above solution at -78 °C during 10 min. After stirring for another 10 min, saturated aqueous

NH_4Cl solution (50 mL) and NaCl solution (75 mL) were added. The layers were separated and the aqueous layer was extracted with EtOAc (4 × 100 mL). The combined organic layers were dried over Na_2SO_4, filtered and concentrated under reduced pressure. The residue was purified by flash column chromatography on silica gel (6:1 petroleum ether-EtOAc) to furnish compound **vii** (4.76 g, 94 %) as a white solid.

Step Ⅱ:

p-TsOH•H_2O (558.7 mg, 2.94 mmol) was added to a solution of **vii** (1.30 g, 3.062 mmol) in Ac_2O (33 mL) at 0 °C. Then the reaction was allowed to warm to 30 °C and stirred for 1 h, before it was added to a solution of $NaHCO_3$ (65.0 g) in H_2O (250 mL) at 0 °C. Stirring continued until the bubbling of CO_2 ceased. The mixture was extracted with EtOAc (3 × 200 mL). The combined organic layers were dried over Na_2SO_4, filtered and concentrated under reduced pressure. The residue was purified by flash column chromatography on silica gel (4:1 petroleum ether-EtOAc) to furnish **viii** (861.3 mg, 60 %) as a pale yellow oil.

Step Ⅲ:

To the solution of the above compound **viii** (860.0 mg, 1.843 mmol) and LiBr (1.122 g, 12.919 mmol) in THF (30 mL) was added DBU (1.966 g, 1.81 mL, 12.914 mmol) at -40 °C. The resulting mixture was allowed stirred at -40°C for 7 h, before it was quenched with saturated aqueous NH_4Cl solution (15 mL), and then H_2O (40 mL) was added to the solution. The solvent was removed under reduced pressure and the aqueous

layer was extracted with EtOAc (5 × 80 mL). The combined organic layers were dried over Na_2SO_4, filtered and concentrated under reduced pressure. The residue was purified by flash column chromatography on silica gel (5:1 petroleum ether-EtOAc) to provide **36** (388.8 mg, 59 %) as a pale yellow oil.

$[\alpha]_D^{22}$ + 15.4 (*c* 0.81, MeOH);

IR (thin film) 2985, 2934, 2873, 1793, 1712, 1612, 1381, 1319, 1218, 1164, 1101, 1064, 1033, 984, 923, 881, 861, 795 cm^{-1};

^1H NMR (400 MHz, $CDCl_3$) δ 6.59 (s, 1H), 4.35 (q, *J* = 7.1 Hz, 2H), 4.08 (d, *J* = 8.3 Hz, 1H), 3.93 (d, *J* = 8.3 Hz, 1H), 3.21 (dd, *J* = 18.9, 3.5 Hz, A of AB, 1H), 2.74 (dd, *J* = 18.9, 13.7 Hz, B of AB, 1H), 2.27 (dd, *J* = 13.7, 3.5 Hz, 1H), 1.73 – 1.62 (m, 2H), 1.44 (s, 3H), 1.37 (t, *J* = 7.2 Hz, 6H), 1.31 (dt, *J* = 5.9, 4.0 Hz, 1H), 1.10 (s, 3H), 0.85 (td, *J* = 8.7, 5.9 Hz, 1H);

^{13}C NMR (100 MHz, $CDCl_3$) δ 165.2, 162.8, 161.5, 148.3, 129.5, 115.8, 110.5, 85.9, 72.0, 61.5, 61.0, 42.6, 30.1, 28.7, 26.8, 26.4, 22.9, 22.9, 14.3, 13.8;

HRMS (ESI^+) m/z calc'd for $C_{20}H_{25}O_6$ $[M+H]^+$: 361.1646, found 361.1640.

Procedure for the Preparation of Compound 39

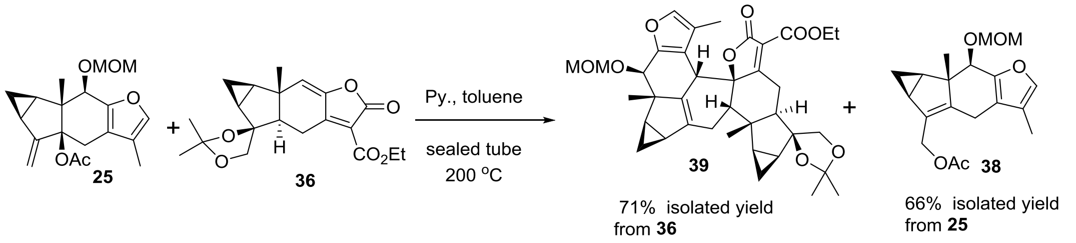

Supplementary Figure 15. Synthesis of compound **39**

A solution of compound **25** (458.1 mg, 1.378 mmol), compound **36** (100.0 mg, 0.277

mmol) and pyridine (439.0 mg, 5.55 mmol) in toluene (0.4 mL) was heated at 200 °C in a sealed tube for 3 h. Then the reaction was cooled to room temperature and purified directly by flash column chromatography on silica gel (6:1 petroleum ether-EtOAc) to furnish compound **39** (124.1 mg, 71% isolated yield from **36**) as a pale yellow solid and compound **38** (302.4 mg, 66% isolated yield from **25**) as a pale yellow oil.

Compound **39**:

$[\alpha]_D^{22}$ – 16.1 (*c* 0.67, $CHCl_3$);

IR (thin film) 2991, 2932, 2863, 2852, 1778, 1716, 1645, 1451, 1372, 1328, 1107, 1039, 992, 920, 875, 867, 803 cm^{-1};

^{1}H NMR (400 MHz, C_6D_6) δ 6.64 (s, 1H), 5.09 (d, *J* = 7.0 Hz, A of AB, 1H), 4.70 (d, *J* = 7.0 Hz, B of AB,1H), 4.56 (d, *J* = 1.9 Hz, 1H), 4.24 (d, *J* = 8.1 Hz, A′ of A′B′, 1H), 4.13 – 4.02 (m, 2H), 3.64 (s, 1H), 3.46 (d, *J* = 8.1 Hz, B′ of A′B′, 1H), 3.30 – 3.22 (m, 1H), 3.27 (s, 3H), 2.49 (ddd, *J* = 16.2, 5.8, 3.3 Hz, 1H), 2.33 (dd, *J* = 16.1, 1.9 Hz, 1H), 2.14 – 1.98 (m, 2H), 1.83 (ddd, *J* = 8.1, 5.9, 4.1 Hz, 1H), 1.68 (dd, *J* = 5.7, 2.0 Hz, 1H), 1.65 (s, 3H), 1.52 – 1.45 (m, 2H), 1.38 – 1.33 (m, 1H), 1.36 (s, 3H), 1.21 (s, 3H), 1.22 – 1.18 (m, 1H), 1.01 (t, *J* = 7.1 Hz, 3H), 0.94 (s, 3H), 0.74 (s, 3H), 0.70 (dd, *J* = 7.8, 4.3 Hz, 1H), 0.53 (td, *J* = 8.8, 5.5 Hz, 1H), 0.25 (td, *J* = 4.2, 2.9 Hz, 1H);

^{13}C NMR (100 MHz, C_6D_6) δ 183.7, 166.9, 161.8, 153.1, 141.8, 140.1, 135.7, 121.5, 120.0, 117.9, 110.1, 97.3, 91.3, 85.5, 76.5, 72.1, 61.2, 59.5, 58.1, 55.8, 55.0, 43.4, 39.5, 31.1, 26.9, 26.6, 26.6, 26.3, 25.6, 25.6, 25.5, 25.1, 16.9, 15.6, 14.1, 13.4, 9.6;

HRMS (ESI$^+$) m/z calc'd for $C_{37}H_{45}O_9$ $[M+H]^+$: 633.3058, found 633.3054.

Compound **38**:

[α] $_D^{22}$ – 36.3 (*c* 2.47, $CHCl_3$);

IR (thin film) 2968, 2932, 2326, 1740, 1685, 1382, 1230, 1150, 1102, 1038, 956, 918, 825, 804, 747, 601 cm^{-1};

^1H NMR (400 MHz, $CDCl_3$) δ 7.10 (s, 1H), 5.08 (d, *J* = 6.8 Hz, A of AB, 1H), 4.90 (d, *J* = 6.8 Hz, B of AB, 1H), 4.66 (q, *J* = 12.3 Hz, 2H), 4.51 (s, 1H), 3.53 (s, 3H), 3.07 (d, *J* = 17.3 Hz, 1H), 2.65 (d, *J* = 18.7 Hz, 1H), 2.07 (s, 3H), 1.92 (s, 3H), 1.88 – 1.81 (m, 2H), 1.09 (s, 3H), 0.81 (td, *J* = 7.7, 4.2 Hz, 1H), 0.17 (q, *J* = 3.8 Hz, 1H);

^{13}C NMR (100 MHz, $CDCl_3$) δ 171.2, 150.2, 140.5, 139.0, 134.6, 119.0, 118.1, 97.6, 78.4, 60.2, 56.0, 53.2, 24.0, 23.3, 21.1, 19.4, 16.8, 14.9, 8.2;

HRMS (ESI^+) m/z calc'd for $C_{19}H_{24}O_5Na$ $[M+Na]^+$:355.1516, found 355.1516.

Procedure for the Preparation of Compound 40

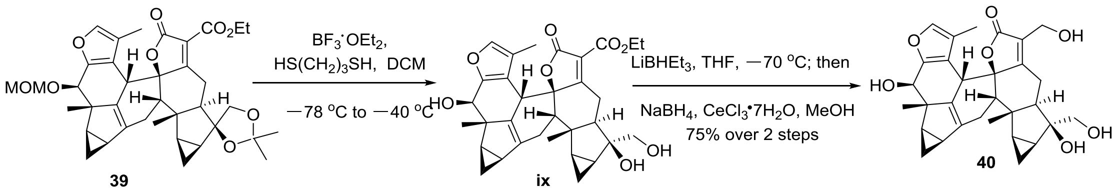

Supplementary Figure 16. Synthesis of compound **40**

Step Ⅰ:

To a solution of compound **39** (112.7 mg, 0.178 mmol) and 1,3-propanedithiol (154.73 mg, 0.14 mL, 1.430 mmol) in DCM (11.6 mL) was added $BF_3{\cdot}OEt_2$ (302.59 mg, 0.26 mL, 2.132 mmol) at -78 °C, and then the reaction was allowed to warm to -40 °C slowly. After stirring at -40 °C for 4 h, MeOH (2 mL) was added to quench the reaction, and then saturated aqueous $NaHCO_3$ solution (15 mL) was added. The layers were separated

and the aqueous layer was extracted with DCM (6× 10 mL). The combined organic layers were dried over Na_2SO_4, filtered and concentrated under reduced pressure. The residue was purified by flash column chromatography on silica gel (EtOAc) to give a crude compound **ix**, which was used in the next reaction without further purification. An aliquot sample was collected, purified and characterized.

$[\alpha]_D^{22}$ – 87.0 (*c* 0.22, $CHCl_3$);

IR (thin film) 3464, 2918, 2851, 1769, 1715, 1641, 1396, 1376, 1338, 1240, 1056, 984, 802, 792, 785 cm^{-1};

^1H NMR (400 MHz, CD_3OD) δ 7.27 (s, 1H), 4.64 (d, *J* = 2.1 Hz, 1H), 4.34 (qt, *J* = 7.1, 3.8 Hz, 2H),, 3.57 (s, 1H), 3.29 (d, *J* = 8.4 Hz, 1H), 3.18 (d, *J* = 10.7 Hz, 1H), 3.00 (dd, *J* = 19.2, 13.5 Hz, 1H), 2.77 (dd, *J* = 16.3, 1.8 Hz, 1H), 2.53 (ddd, *J* = 16.3, 6.1, 3.3 Hz, 1H), 2.04 – 1.98 (m, 1H), 1.98 – 1.91 (m, 2H), 1.90 (d, *J* = 5.5 Hz, 1H), 1.77 (dd, *J* = 6.2, 1.8 Hz, 1H), 1.74 (d, *J* = 1.2 Hz, 3H), 1.61 (td, *J* = 8.1, 4.1 Hz, 1H), 1.51 (ddd, *J* = 9.1, 7.4, 3.6 Hz, 1H), 1.33 (t, *J* = 7.1 Hz, 3H), 1.20 (dt, *J* = 5.5, 3.9 Hz, 1H), 0.93 (dt, *J* = 7.7, 3.9 Hz, 1H), 0.86 (s, 3H), 0.82 (s, 3H), 0.69 (td, *J* = 8.7, 5.5 Hz, 1H), 0.33 (q, *J* = 4.1, 1H);

^{13}C NMR (100 MHz, CD_3OD) δ 187.3, 169.7, 162.5, 155.1, 142.6, 141.3, 136.4, 120.9, 120.9, 117.7, 93.4, 79.3, 73.9, 69.8, 62.4, 60.8, 59.5, 56.3, 45.1, 40.2, 29.3, 27.0, 26.9, 26.4, 26.0, 25.9, 25.8, 16.3, 15.7, 14.5, 12.8, 9.8;

HRMS (ESI^+) m/z calc'd for $C_{32}H_{36}O_8Na$ $[M+Na]^+$: 571.2302, found 571.2297.

Step Ⅱ:

To a solution of above compound **ix** in THF (7.6 mL) was added $LiHBEt_3$ (1.0 M in THF, 0.87 mL, 0.867 mmol) at -70 °C. After stirring at this temperature for 1 h, MeOH (7.6 mL) was added. The resulting mixture was warmed to 0 °C, and $CeCl_3$•$7H_2O$ (269.2 mg, 0.723 mmol) and $NaBH_4$ (54.6 mg, 1.443 mmol) were added in order. The resulting mixture was stirred at 0 °C for 0.5 h, before it was quenched with saturated $Na_2S_2O_3$ solution (10 mL), The solvent was removed under reduced pressure and the aqueous layer was extracted with EtOAc (5 × 6 mL). The combined organic layers were dried over Na_2SO_4, filtered and concentrated under reduced pressure. The residue was purified by flash column chromatography on silica gel (15:1 EtOAc – MeOH) to provide **40** (68.0 mg, 75% over 2 steps) as a pale yellow solid.

$[\alpha]_D^{22}$ – 12.0 (*c* 0.16, THF);

IR (thin film) 3365, 2973, 2918, 2837, 1756, 1744, 1488, 1403, 1279, 1090, 1049, 881, 817, 792 cm^{-1};

^1H NMR (400 MHz, CD_3OD) δ 7.24 (s, 1H), 4.63 (s, 1H), 4.29 (s, 2H), 3.52 (s, 1H), 3.27 (d, *J* = 10.5 Hz, 1H), 3.17 (d, *J* = 10.8 Hz, 1H), 2.75 – 2.62 (m, 2H), 2.51 (ddd, *J* = 16.2, 6.0, 3.3 Hz, 1H), 1.96 (td, *J* = 4.2, 1.9 Hz, 1H), 1.94 – 1.89 (m, 2H), 1.84 (dd, *J* = 27.8, 5.6 Hz, 1H), 1.77 (s, 3H), 1.69 (dd, *J* = 6.0, 1.8 Hz, 1H), 1.60 (td, *J* = 8.1, 4.1 Hz, 1H), 1.50 (ddd, *J* = 9.0, 7.5, 3.6 Hz, 1H), 1.23 – 1.18 (m, 1H), 0.93 (dd, *J* = 7.9, 4.3 Hz, 1H), 0.90 (s, 3H), 0.81 (s, 3H), 0.68 (td, *J* = 8.7, 5.5 Hz, 1H), 0.32 (td, *J* = 4.1, 3.0 Hz, 1H);

^{13}C NMR (100 MHz, CD_3OD) δ 174.6, 173.0, 154.7, 142.5, 141.0, 136.8, 127.5, 121.2, 118.3, 93.7, 79.4, 74.0, 69.8, 68.9, 60.9, 59.6, 56.2, 55.0, 45.1, 39.5, 29.2, 27.0, 26.5,

26.0, 25.7, 23.8, 16.3, 15.7, 12.8, 10.2;

HRMS (ESI$^+$) m/z calc'd for $C_{30}H_{34}O_7Na$ $[M+Na]^+$: 529.2197, found 529.2203.

Procedure for the Preparation of 11 (sarglabolide I)

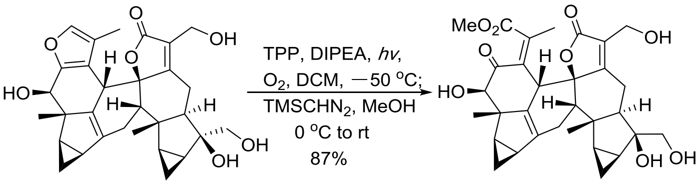

Supplementary Figure 17. Synthesis of compound **11**

Oxygen was bubbled through a solution of compound **40** (10.0 mg, 0.0197 mmol) in DCM (5.4 mL) containing a catalytic amount of tetraphenylporphyrin (TPP, 1.82 mg, 2.96 μmol) and *N,N*-diisopropylethylamine (3.18 mg, 4.07 μL, 0.0246 mmol) at -50 °C. After the mixture was irradiated with a high voltage sodium lamp (100 W, Galilee®) for 5 min, irradiation was discontinued, but oxygen was still passed through the solution for additional 5 minutes. Then the cooling bath was removed, and MeOH (2.7 mL) and $TMSCHN_2$ (2.0 M in hexane, 0.500 mL) was added in order at 0 °C. The resulting mixture was allowed to stir at room temperature for 5 min, and then the mixture was concentrated under reduced pressure. The residue was purified by flash column chromatography on silica gel (20:1, DCM/MeOH) to furnish compound **11** (sarglabolide I, 9.5 mg, 87%) as a pale yellow solid.

$[\alpha]_D^{22}$ – 58.0 (*c* 0.30, MeOH) {Lit. $[\alpha]_D^{23}$ – 68.3 (*c* 0.29, MeOH)}[5];

IR (thin film) 3470, 2950, 2914, 11735, 1623, 1600, 1436, 1386, 1278, 1203, 1086, 1038, 990, 800, 737, 665 cm^{-1};

^{1}H NMR (400 MHz, CD_3OD) δ 4.26 (d, *J* = 13.2 Hz, 1H), 4.23 (d, *J* = 13.2 Hz, 1H), 4.04 (s, 1H), 3.94 (d, *J* = 3.9 Hz, 1H), 3.77 (s, 3H), 3.35 (d, *J* = 10.9 Hz, 1H), 3.31 (d, *J* = 10.9 Hz, 1H), 2.84 (br. d, *J* = 16.3 Hz, 1H), 2.77 (dd, *J* = 18.4, 14.0 Hz, 1H), 2.53 (ddd, *J* = 16.4, 6.2, 4.0 Hz, 1H), 2.24 (dd, *J* = 18.2, 6.0 Hz, 1H), 2.03 – 1.97 (m, 1H), 1.95 – 1.91 (m, 1H), 1.85 (s, 3H), 1.81 (dd, *J* = 6.1, 1.8 Hz, 1H), 1.70 (dd, *J* = 13.8, 5.9 Hz, 1H), 1.60 (dd, *J* = 8.3, 3.9 Hz, 1H), 1.56 – 1.49 (m, 1H), 1.22 (dt, *J* = 5.6, 3.9 Hz, 1H), 1.01 (s, 3H), 0.98 (td, *J* = 7.6, 4.1 Hz, 1H), 0.91 (s, 3H), 0.68 (td, *J* = 8.7, 5.4 Hz, 1H), 0.32 (dd, *J* = 7.2, 3.6 Hz, 1H);

^{13}C NMR (100 MHz, CD_3OD) δ 202.1, 174.2, 173.1, 171.6, 147.1, 143.2, 134.4, 133.7, 128.5, 94.7, 81.2, 79.4, 69.2, 60.6, 57.3, 54.3, 53.0, 52.6, 45.8, 41.9, 29.1, 27.1, 26.5, 26.3, 26.0, 25.4, 23.0, 20.5, 16.1, 15.8, 12.7;

HRMS (ESI^+) m/z calc'd for $C_{31}H_{36}O_9Na$ $[M+Na]^+$: 575.2252, found 575.2248.

Procedure for the Preparation of 12 (multistalide B)

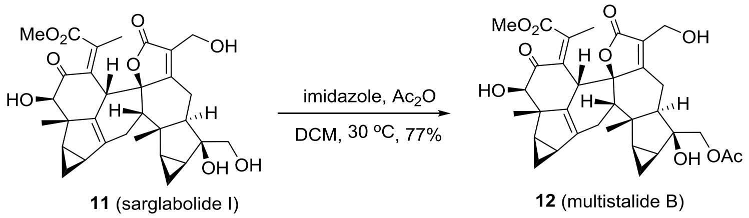

11 (sarglabolide I) **12** (multistalide B)

Supplementary Figure 18. Synthesis of compound **12**

To a solution of compound 11 (sarglabolide I, 10.0 mg, 0.0181 mmol) in DCM (6.00 mL), was added imidazole (61.6 mg, 0.905 mmol) and Ac_2O (92.4 mg, 86 μL, 0.905 mmol) at 0 °C. The resulting mixture was stirred at 30 °C for 10 h, before it was quenched with saturated aqueous $NaHCO_3$ solution (5.0 mL) at 0 °C. The layers were

separated and the aqueous layer was extracted with DCM (5 × 5.0 mL). The combined organic layers were dried over Na_2SO_4, filtered and concentrated under reduced pressure. The residue was purified by flash column chromatography on silica gel (1:2 petroleum ether-EtOAc) to provide compound **12** (multistalide B, 8.3 mg, 77 %) as a colorless oil.

$[\alpha]_D^{22}$ – 103.0 (*c* 0.06, $CHCl_3$) {Lit. $[\alpha]_D^{23}$ – 113 (*c* 0.155, $CHCl_3$)}[6];

IR (thin film) 3430, 2959, 2918, 2850, 2326, 1740, 1735, 1586, 1572, 1807, 1435, 1352, 1277, 1262, 1055, 761, 749 cm^{-1};

^1H NMR (800 MHz, $CDCl_3$) δ 4.42 (d, *J* = 12.7 Hz, 1H), 4.35 (d, *J* = 14.2 Hz, 1H), 4.08 (d, *J* = 11.6 Hz, 1H), 3.94 (s, 1H), 3.93 (d, *J* = 3.9 Hz, 1H), 3.82 (d, *J* = 11.7 Hz, 1H), 3.80 (s, 3H), 3.36 (s, 1H), 2.80 (d, *J* = 16.3 Hz, 1H), 2.69 (dd, *J* = 18.3, 13.7 Hz, 1H), 2.58 (dt, *J* = 16.3, 5.2 Hz, 1H), 2.48 (s, 1H), 2.32 (dd, *J* = 18.3, 6.0 Hz, 1H), 2.13 (s, 3H), 2.07 (dt, *J* = 9.1, 4.7 Hz, 1H), 1.94 (s, 3H), 1.92 (d, *J* = 1.8 Hz, 1H), 1.88 – 1.85 (m, 1H), 1.85 – 1.83 (m, 1H), 1.79 (s, 1H), 1.62 (td, *J* = 8.1, 4.1 Hz, 1H), 1.46 (td, *J* = 8.4, 3.6 Hz, 1H), 1.27 (d, *J* = 4.4 Hz, 1H), 1.01 (s, 3H), 1.00 – 0.98 (m, 1H), 0.88 (s, 3H), 0.72 (td, *J* = 8.8, 5.8 Hz, 1H), 0.32 (q, *J* = 4.0 Hz, 1H);

^{13}C NMR (200 MHz, $CDCl_3$) δ 200.2, 172.5, 171.4, 171.1, 168.3, 147.8, 142.7, 132.2, 131.0, 127.4, 93.6, 80.2, 77.3, 71.7, 60.2, 55.2, 55.1, 52.9, 51.2, 45.0, 41.1, 28.4, 26.4, 26.1, 25.7, 25.4, 25.0, 22.5, 21.0, 20.7, 16.1, 15.6, 11.9;

HRMS (ESI$^+$) m/z calc'd for $C_{33}H_{38}O_{10}Na$ $[M+Na]^+$: 617.2357, found 617.2358.

Procedure for the Preparation of 13 (shizukaol C)

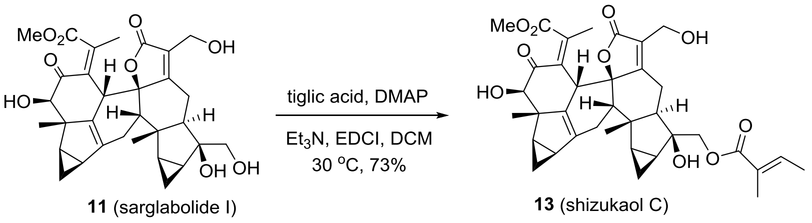

Supplementary Figure 19. Synthesis of compound **13**

To a solution of compound **11** (7.4 mg, 0.0134 mmol) in DCM (2.5 mL), were added Et_3N (6.78 mg, 0.0670 mmol, 9.3 μL), tiglic acid (2.7 mg, 0.0270 mmol), EDCI (5.1 mg, 0.0266 mmol) and DMAP (0.3 mg, 0.00246 mmol) in order. The resulting mixture was stirred at 30 °C for 19 h and purified directly by flash column chromatography on silica gel (1:2 petroleum ether-EtOAc) to furnish compound **13** (shizukaol C, 6.2 mg, 73%) as a colorless oil.

$[\alpha]_D^{22}$ – 66.9 (*c* 0.12, $CHCl_3$) {Lit. $[\alpha]_D^{23}$ – 65 (*c* 1.9, $CHCl_3$)}[7];

IR (thin film) 3467, 2958, 2925, 2854, 1735, 1652, 1459, 1446, 1382, 1262, 1120, 1103, 1026, 1007, 833, 800, 737 cm^{-1};

^1H NMR (400 MHz, Pyridine-d_5) δ 6.92 (qd, *J* = 6.8, 3.4 Hz, 1H), 6.30 (s, 1H, -*OH* exchangeable with D_2O), 4.81 (d, *J* = 13.0 Hz, 1H), 4.76 (d, *J* = 12.9 Hz, 1H), 4.57 (d, *J* = 11.3 Hz, 1H), 4.45 (s, 1H), 4.30 (d, *J* = 11.4 Hz, 1H), 4.25 (d, *J* = 3.7 Hz, 1H), 3.70 (s, 3H), 3.48 (dd, *J* = 18.1, 13.8 Hz, 1H), 2.99 (dd, *J* = 18.1, 6.0 Hz, 1H), 2.84 (dd, *J* = 16.2, 1.8 Hz, 1H), 2.69 (dt, *J* = 16.3, 4.6 Hz, 1H), 2.36 (ddd, *J* = 8.2, 5.8, 4.1 Hz, 1H), 2.18 (s, 3H), 2.17 – 2.12 (m, 1H), 1.97 (ddd, *J* = 13.1, 6.3, 2.3 Hz, 1H), 1.81 (d, *J* = 1.4 Hz, 3H), 1.73 (td, *J* = 8.0, 4.2 Hz, 1H), 1.60 – 1.59 (m, 1H), 1.58 (d, *J* = 6.0 Hz, 2H), 1.56 – 1.54 (m, 2H), 1.35 (s, 3H), 1.21 (s, 3H), 0.99 (td, *J* = 7.8, 4.2 Hz, 1H), 0.91 – 0.84 (m, 1H), 0.77 (ddd, *J* = 9.2, 9.0, 6.3 Hz, 1H), 0.40 (q, *J* = 3.8 Hz, 1H);

^{13}C NMR (200 MHz, $CDCl_3$) δ 200.1, 172.4, 171.2, 168.4, 168.4, 147.6, 142.8, 139.1, 132.4, 130.9, 128.1, 127.6, 93.7, 80.1, 77.7, 71.2, 60.0, 55.1, 55.0, 52.9, 51.2, 45.0, 41.3, 28.5, 26.3 (2C), 25.7, 25.4, 25.0, 22.4, 20.6, 16.2, 15.7, 14.3, 12.3, 11.9;

HRMS (ESI$^+$) m/z calc'd for $C_{36}H_{42}O_{10}Na$ $[M+Na]^+$: 657.2670, found 657.2665.

Procedure for the Preparation of 15 (shizukaol I)

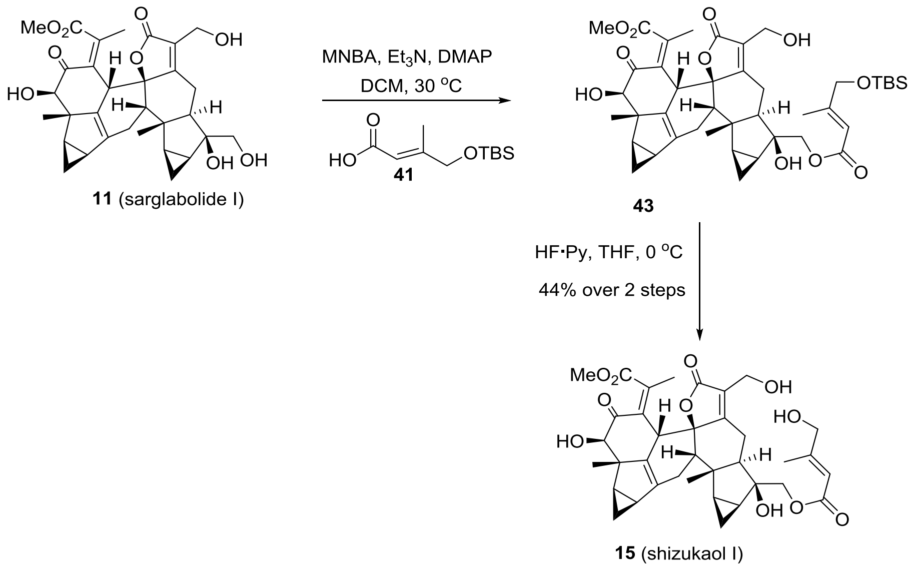

Supplementary Figure 20. Synthesis of compound **15**

Known compound **41** can be prepared according to the known procedure[8].

Step Ⅰ:

To a solution of compound **11** (9.0 mg, 0.0163 mmol) and **41** (6.76 mg, 0.0293 mmol) in DCM (4.1 mL), were added Et_3N (5.4 mg, 0.0534 mmol, 7.5 μL), DMAP (3.0 mg, 0.0246 mmol) and MNBA (2-methyl-6-nitrobenzonic anhydride, 10.1 mg, 0.0293 mmol) in order. The mixture was allowed to stir at 30 °C for 3 h, and then purified directly by flash column chromatography on silica gel (pre-treated with Et_3N, 1:1 petroleum ether-EtOAc) to furnish crude compound **43**. Because of its instability,

compound **43** was used in the next reaction quickly without further purification.

Step Ⅱ:

To a solution of above compound **43** in THF (6 mL) was added aqueous HF•Py (70% in water, 0.3 mL) at 0 °C. Then the reaction was allowed to stirred for 1.5 h at 0 °C, before it was added to saturated aqueous $NaHCO_3$ (30 mL) at 0 °C. The mixture was extracted with EtOAc (5 × 10 mL). The combined organic layers were dried over Na_2SO_4, filtered and concentrated under reduced pressure. The residue was purified by flash column chromatography on silica gel (EtOAc) to provide **15** (shizukaol I, 4.7 mg, 44% over 2 steps) as a colorless oil.

$[\alpha]_D^{22}$ – 111.8 (*c* 0.03, $CHCl_3$) {Lit. $[\alpha]_D^{23}$ – 122 (*c* 3.40, $CHCl_3$)}[9];

IR (thin film) 3440, 2960, 2918, 2850, 2328, 1750, 1734, 1652, 1477, 1460, 1423, 1090, 1020, 800, 655, 633 cm^{-1};

^1H NMR (800 MHz, $CDCl_3$) δ 6.00 (br. s, 1H), 4.40 (d, *J* = 13.5 Hz, 1H), 4.33 (d, *J* = 13.5 Hz, 1H), 4.30 (d, *J* = 11.8 Hz, 1H), 4.18 (d, *J* = 9.3 Hz, 2H), 4.01 (s, 1H), 3.93 (br. d, *J* = 3.3 Hz, 1H), 3.81 (d, *J* = 11.9 Hz, 1H), 3.77 (s, 3H), 3.43 (s, 1H, -*OH* exchangeable with D_2O), 2.86 (s, 1H, -*OH* exchangeable with D_2O), 2.83 (d, *J* = 16.4 Hz, 1H), 2.68 (dd, *J* = 18.2, 13.6 Hz, 1H), 2.59 – 2.55 (m, 1H), 2.53 (br. s, 1H, -*OH* exchangeable with D_2O), 2.30 (dd, *J* = 18.2, 5.9 Hz, 1H), 2.16 (s, 1H, -*OH* exchangeable with D_2O), 2.12 (s, 3H), 2.08 (dd, *J* = 8.8, 4.8 Hz, 1H), 1.94 (d, *J* = 6.4 Hz, 1H), 1.94 (dd, *J* = 14.4, 6.4 Hz, 1H), 1.93 (s, 3H), 1.87 (ddd, *J* = 5.6, 4.8, 3.2 Hz, 1H), 1.64 (ddd, *J* = 8.0, 7.2, 4.0 Hz, 1H), 1.47 (ddd, *J* = 8.8, 7.2, 3.2 Hz, 1H), 1.32 –

1.26 (m, 1H), 1.03 – 1.00 (m, 1H), 0.99 (s, 3H), 0.87 (s, 3H), 0.71 (td, J = 8.7, 5.9 Hz, 1H), 0.33 (dd, J = 7.3, 4.0 Hz, 1H).

^{13}C NMR (100 MHz, $CDCl_3$) δ 199.8, 172.6, 171.7, 168.5, 166.9, 160.2, 147.7, 142.6, 132.2, 131.0, 127.2, 112.4, 93.6, 80.0, 77.6, 71.8, 67.3, 60.2, 55.1, 55.0, 53.1, 51.2, 45.0, 41.1, 28.5, 26.4, 26.1, 25.7, 25.4, 25.0, 22.7, 20.6, 16.1, 15.9, 15.7, 11.7;

HRMS (ESI$^+$) m/z calc'd for $C_{36}H_{42}O_{11}Na$ [M+Na]$^+$: 673.2619, found 673.2614.

Procedure for the Preparation of 16 (chlorajaponilide C)

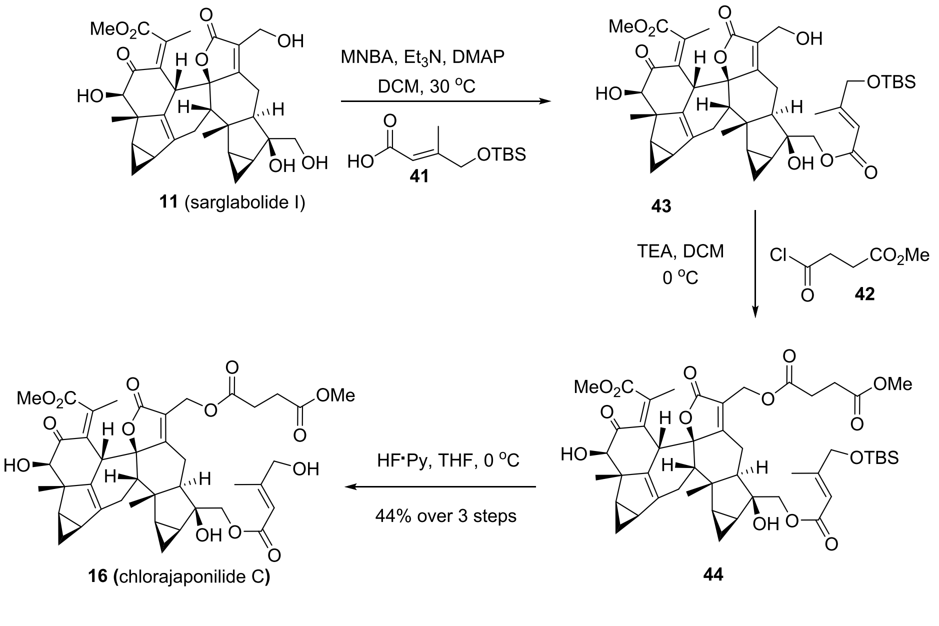

Supplementary Figure 21. Synthesis of compound **16**

Step Ⅰ:

To a solution of compound **11** (10.0 mg, 0.0181 mmol) and **41** (7.5 mg, 0.0326 mmol) in DCM (4.5 mL) were added Et_3N (6.04 mg, 0.0597 mmol, 8.3 μL), DMAP (3.3 mg, 0.0270 mmol) and MNBA (2-methyl-6-nitrobenzonic anhydride, 11.2 mg, 0.0325 mmol) in order. The mixture was allowed to stir at 30 °C for 3 h, and then purified

directly by flash column chromatography on silica gel (pre-treated with Et_3N, 1:1 petroleum ether-EtOAc) to furnish crude compound **43**. Because of its instability compound **43** was used in the next reaction quickly without further purification.

Step Ⅱ:

To a solution of above compound **43** and Et_3N (11.0 mg, 15 μL, 0.1086 mmol) in DCM (5 mL) was added compound **42** (8.2 mg, 6.6 μL, 0.0543 mmol) at 0 °C. Then the reaction was allowed to stirred for 3 h at 0 °C, before it was added to saturated aqueous $NaHCO_3$ (5 mL) at 0 °C. The mixture was extracted with DCM (5 × 5 mL). The combined organic layers were dried over Na_2SO_4, filtered and concentrated under reduced pressure to furnish crude compound **44.** Compound **44** was used in the next reaction without further purification.

Step Ⅲ:

To a solution of above compound **44** in THF (8 mL) was added aqueous HF•Py (70% in water, 0.4 mL) at 0 °C. Then the reaction was allowed to stir for 1.5 h at 0 °C, before it was added to saturated aqueous $NaHCO_3$ solution (40 mL) at 0 °C. The mixture was extracted with EtOAc (6 × 10 mL). The combined organic layers were dried over Na_2SO_4, filtered and concentrated under reduced pressure. The residue was purified by flash column chromatography on silica gel (1:2 petroleum ether-EtOAc) to furnish **16** (chlorajaponilide C, 6.1 mg, 44% over 3 steps) as a colorless oil.

$[\alpha]_D^{22}$ – 60.9 (*c* 0.06, MeOH) {Lit. $[\alpha]_D^{20}$ – 168.0 (*c* 0.2, MeOH)}[10];

IR (thin film) 3469, 2923, 1731, 1601, 1438, 1360, 1280, 1218, 1084, 989 cm^{-1};

^1H NMR (400 MHz, $CDCl_3$) δ 5.97 (d, *J* = 1.9 Hz, 1H), 5.00 (d, *J* = 13.5 Hz, 1H), 4.77 (d, *J* = 13.5 Hz, 1H), 4.32 (d, *J* = 11.8 Hz, 1H), 4.17 (d, *J* = 6.3 Hz, 2H), 3.99 (s, 1H), 3.93 (d, *J* = 3.5 Hz, 1H), 3.73 (s, 3H), 3.69 (d, *J* = 3.6 Hz, 1H), 3.66 (s, 3H), 3.37 (s, 1H, -*OH* exchangeable with D_2O), 3.17 (t, *J* = 6.7 Hz, 1H, -*OH* exchangeable with D_2O), 2.81 – 2.77 (m, 1H), 2.76 – 2.73 (m, 2H), 2.69 (d, *J* = 4.6 Hz, 1H), 2.68 – 2.64 (m, 2H), 2.60 (br. d, *J* = 7.8 Hz, 1H), 2.60 (br. s, 1H, -*OH* exchangeable with D_2O), 2.51 (dd, *J* = 18.3, 5.8 Hz, 1H), 2.12 (s, 3H), 2.08 – 2.03 (m, 1H), 1.88 (d, *J* = 3.9 Hz, 3H), 1.88 – 1.85 (br. d, *J* = 8.0, 1H), 1.85 – 1.84 (br. d, *J* = 6.0, 2H), 1.62 – 1.58 (m, 1H), 1.45 – 1.39 (m, 1H), 1.32 (d, *J* = 5.1 Hz, 1H), 1.02 – 0.91 (m, 1H) , 0.99 (s, 3H), 0.86 (s, 3H), 0.72 (td, *J* = 8.8, 6.0 Hz, 1H), 0.29 (q, *J* = 4.1 Hz, 1H);

^{13}C NMR (200 MHz, $CDCl_3$) δ 199.9, 173.3, 172.3, 172.1, 171.5, 171.2, 166.7, 159.7, 147.6, 142.4, 131.8, 131.5, 123.5, 112.6, 93.4, 80.2, 77.4, 71.8, 67.3, 61.1, 55.9, 55.8, 52.9, 52.2, 51.2, 44.9, 40.9, 28.8, 28.7, 27.9, 26.6, 25.8, 25.6, 25.4, 24.9, 23.7, 20.6, 16.0, 15.8, 15.4, 11.7.

HRMS (ESI^+) m/z calc'd for $C_{41}H_{48}O_{14}Na$ $[M+Na]^+$: 787.2936, found 787.2936.

Procedure for the Preparation of 46

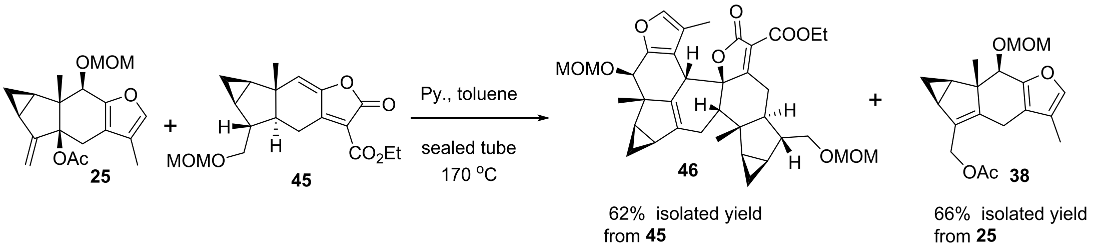

Supplementary Figure 22. Synthesis of compound **46**

Known compound **45** was prepared by following the known procedure in our previous

work[2].

A solution of compound **25** (94.9 mg, 0.285 mmol), compound **45** (20.0 mg, 0.0574 mmol) and pyridine (54.6 mg, 0.690 mmol) in toluene (0.75 mL) was heated at 170 °C in a sealed tube for 20 h. Then the reaction was cooled to room temperature and purified directly by flash column chromatography on silica gel (4:1 petroleum ether-EtOAc) to furnish compound **46** (22.1 mg, 62% isolated yield from **45**) as a pale yellow oil and compound **38** (62.8 mg, 66% isolated yield from **25**) as a pale yellow oil. The analytical data of **46** are in excellent agreement with our reported data[2].

Procedure for the Preparation of 48

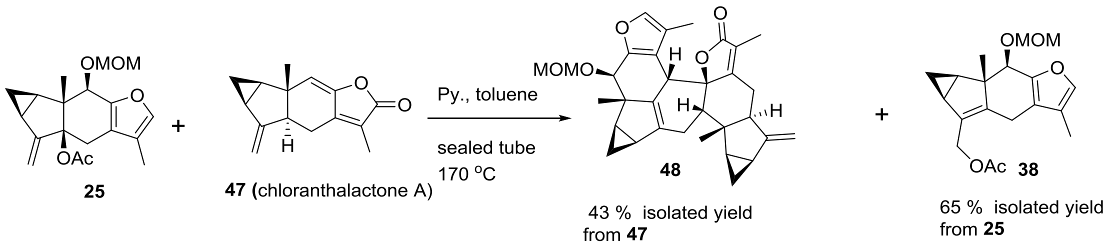

Supplementary Figure 23. Synthesis of compound **48**

Compound **47** was prepared according to our previous work[1,2,11].

A solution of compound **25** (144.6 mg, 0.435 mmol), compound **47** (20.0 mg, 0.0876 mmol) and pyridine (90.0 mg, 1.138 mmol) in toluene (2.0 mL) was heated at 170 °C in a sealed tube for 20 h. Then the reaction was cooled to room temperature and purified directly by flash column chromatography on silica gel (15:1 petroleum ether-EtOAc) to furnish compound **48** (18.9 mg, 43% isolated yield from **47**) as a colorless oil and compound **38** (94.0 mg, 65% isolated yield from **25**) as a pale yellow oil. Part of compound **47** (4.2 mg) was recovered in 21 % yield.

Compound **48:**

$[\alpha]_D^{22}$ – 11.8 (*c* 0.29, $CHCl_3$);

IR (thin film) 2092, 2941, 2833, 1707, 1483, 1432, 1395, 1387, 1240, 1206, 1160, 1130, 1105, 993, 883, 872, 795 cm^{-1};

^{1}H NMR (400 MHz, C_6D_6) δ 6.81 (s, 1H), 5.11 (d, *J* = 6.8 Hz, 1H), 4.91 (d, *J* = 1.2 Hz, 1H), 4.82 (d, *J* = 6.8 Hz, 1H), 4.65 (s, 1H), 4.40 (s, 1H), 3.68 (s, 1H), 3.28 (s, 3H), 2.87 – 2.78 (m, 1H), 2.57 (ddd, *J* = 15.9, 5.4, 3.2 Hz, 1H), 2.41 (dd, *J* = 15.9, 1.8 Hz, 1H), 2.00 (ddd, *J* = 8.3, 6.0, 4.2 Hz, 1H), 1.88 (br. s, 1H), 1.82 (dd, *J* = 16.8, 12.8 Hz, 1H), 1.74 (dd, *J* = 5.4, 2.0 Hz, 1H), 1.63 (d, *J* = 1.2 Hz, 3H), 1.61 – 1.56 (m, 1H), 1.54 (d, *J* = 1.0 Hz, 3H), 1.52 – 1.50 (m, 1H), 1.43 (td, *J* = 7.7, 3.8 Hz, 1H), 1.01 (s, 3H), 0.76 (td, *J* = 7.8, 4.4 Hz, 1H), 0.68 – 0.63 (m, 1H), 0.63 – 0.57 (m, 1H), 0.34 (dd, *J* = 7.3, 4.1 Hz, 1H), 0.29 (s, 3H);

^{13}C NMR (100 MHz, C_6D_6) δ 172.9, 165.0, 153.3, 151.1, 141.4, 139.7, 137.0, 124.3, 120.1, 118.1, 105.6, 97.8, 91.7, 77.7, 60.3, 57.0, 55.8, 55.1, 41.7, 38.9, 30.2, 25.9, 25.8, 24.9, 24.4, 23.8, 23.3, 17.4, 16.7, 15.7, 9.4, 8.9;

HRMS (ESI$^+$) m/z calc'd for $C_{32}H_{37}O_5$ $[M+H]^+$: 501.2636, found 501.2636.

Procedure for the Preparation of 4 (shizukaol A)

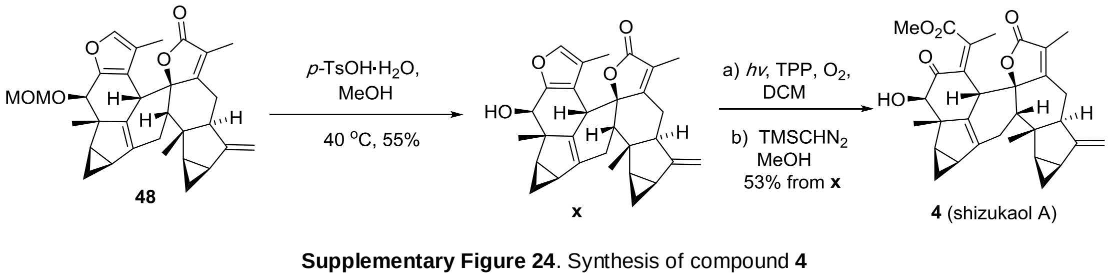

Supplementary Figure 24. Synthesis of compound **4**

Step Ⅰ:

p-TsOH•H_2O (38.0 mg, 0.200 mmol) was added to a solution of **48** (10 mg, 0.020 mmol) in MeOH (4 mL) at 0 °C. Then the reaction was allowed to warm to 40 °C and stir for 5 h, before it was quenched with saturated aqueous $NaHCO_3$ solution (4 mL) at 0 °C. The mixture was extracted with DCM (5 × 4 mL). The combined organic layers were dried over Na_2SO_4, filtered and concentrated under reduced pressure. The residue was purified by flash column chromatography on silica gel (5:1 petroleum ether-EtOAc) to furnish compound **x** (5.1 mg, 55 %) as a colorless oil.

$[\alpha]_D^{22}$ – 132.0 (*c* 0.08, $CHCl_3$);

IR (thin film) 2965, 2918, 2851, 2328, 1749, 1682, 1667, 1263, 1102, 1020, 997, 970, 813, 740 cm^{-1};

^1H NMR (400 MHz, $CDCl_3$) δ 7.12 (s, 1H), 4.83 (s, 1H), 4.57 (s, 1H), 4.41 (s, 1H), 3.57 (brs, 1H), 2.76 – 2.68 (m, 2H), 2.60 (ddd, *J* = 16.0, 5.4, 3.2 Hz, 1H), 2.14 (br. s, 1H, -*OH* exchangeable with D_2O), 2.05 (dd, *J* = 17.1, 13.0 Hz, 1H), 1.96 (ddd, *J* = 8.3, 6.0, 4.2 Hz, 1H), 1.90 – 1.86 (m, 3H), 1.80 (d, *J* = 1.5 Hz, 3H), 1.70 (d, *J* = 1.0 Hz, 3H), 1.64 – 1.54 (m, 2H), 0.92 (td, *J* = 7.8, 4.4 Hz, 1H), 0.84 – 0.77 (m, 1H), 0.80 (s, 3H), 0.76 – 0.69 (m, 1H), 0.46 (s, 3H), 0.36 (dd, *J* = 7.4, 4.1 Hz, 1H).

^{13}C NMR (200 MHz, $CDCl_3$) δ 173.9, 166.6, 152.8, 150.6, 141.5, 139.9, 136.1, 123.8, 120.2, 117.4, 106.1, 92.3, 72.9, 60.0, 56.5, 55.1, 41.7, 38.4, 25.8, 25.7, 25.3, 25.0, 24.0, 24.0, 23.0, 16.6, 16.2, 15.6, 9.6, 9.0;

HRMS (ESI^+) m/z calc'd for $C_{30}H_{33}O_4$ $[M+H]^+$: 457.2373, found 457.2379.

Step Ⅱ:

Oxygen was bubbled through a solution of compound **x** (6.0 mg, 0.0131 mmol) in DCM (6 mL) containing a catalytic amount of tetraphenylporphyrin (TPP, 1.2 mg, 1.97 μmol) at -20 °C. After the mixture was irradiated with a high voltage sodium lamp (100 W, OSRAM®) for 2 min, irradiation was discontinued. After oxygen was bubbled through the solution for additional 5 min, saturated solution of oxalic acid (5 mL) was added. The mixture was extracted with DCM (5 × 4 mL). The combined organic layers were dried over Na_2SO_4, filtered and concentrated under reduced pressure to get the crude intermediate. The intermediate was dissolved in MeOH (5.00 mL) at 0 °C, and $TMSCHN_2$ (2.0 M in hexane, 0.500 mL) was added at 0 °C. After stirring for 5 min at 0 °C, the reaction was quenched with buffer solution (pH = 7.0, 0.1 M, 5 mL). The mixture was extracted with DCM (5 × 5 mL). The combined organic layers were dried over Na_2SO_4, filtered and concentrated under reduced pressure. The residue was purified by flash column chromatography on silica gel (5:1 petroleum ether-EtOAc) to furnish **4** (shizukaol A, 3.5 mg, 53 %) as a colorless oil.

$[\alpha]_D^{22}$ – 167.2 (*c* 0.06, $CHCl_3$) {Lit. $[\alpha]_D^{23}$ – 216.0 (*c* 0.54, $CHCl_3$)}[12]

IR (thin film) 3450, 2955, 2918, 2850, 1750, 1687, 1602, 1543, 1436, 1375, 1301, 1226, 1179, 1084, 994, 951, 886, 799 cm^{-1};

^1H NMR (400 MHz, $CDCl_3$) δ 4.91 (t, *J* = 1.9 Hz, 1H), 4.58 (t, *J* = 2.2 Hz, 1H), 3.88 (d, *J* = 4.2 Hz, 1H), 3.87 (s, 1H), 3.80 (s, 3H), 3.20 (s, 1H, *-OH* exchangeable with D_2O), 2.77 (dd, *J* = 16.3, 2.0 Hz, 1H), 2.63 (ddd, *J* = 16.3, 5.6, 3.8 Hz, 1H), 2.50 – 2.41 (m, 1H), 2.30 – 2.24 (m, 1H), 2.21 dd (*J* = 18.8, 6.0 Hz, 1H), 2.02 (ddd, *J* = 8.2, 5.7,

4.2 Hz, 1H), 1.91 (dd, J = 5.8, 2.0 Hz, 1H), 1.86 (br. dd, J = 6.8, 2.0, 2H), 1.81 (s, 6H), 1.56 (ddd, J = 7.8, 7.4, 3.9 Hz, 1H), 1.01 (s, 3H), 0.99 – 0.95 (m, 1H), 0.82 (td, J = 8.6, 5.4 Hz, 1H), 0.76 (dt, J = 5.4, 3.7 Hz, 1H), 0.51 (s, 3H), 0.30 (td, J = 4.3, 2.9 Hz, 1H); ^{13}C NMR (100 MHz, $CDCl_3$) δ 200.2, 173.7, 171.1, 165.4, 149.0, 147.0, 142.4, 132.0, 132.0, 124.5, 107.4, 93.1, 79.7, 59.9, 54.5, 52.8, 51.2, 41.9, 41.0, 25.9, 25.8, 25.1, 25.0, 23.8, 23.2, 23.0, 20.3, 16.7, 16.0, 15.5, 8.8;

HRMS (ESI$^+$) m/z calc'd for $C_{31}H_{34}O_6Na$ [M+Na]$^+$: 525.2248, found 525.2247.

Procedure for the Preparation of 48 from 49

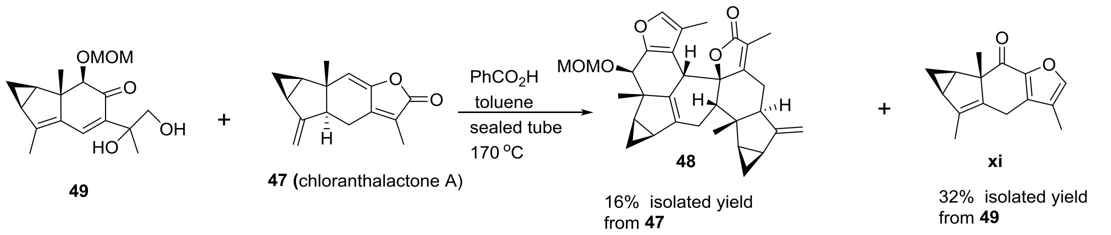

Supplementary Figure 25. Synthesis of compound **48** from **49**

Known compound **49** was prepared according to the known procedure [2].

A solution of compound **49** (405.7 mg, 1.316 mmol), compound **47** (100 mg, 0.438 mmol) and benzoic acid (535.6 mg, 4.386 mmol) in toluene (5.0 mL) was heated at 170 °C in a sealed tube for 7 h. Then the reaction was cooled to room temperature, before it was quenched with saturated aqueous $NaHCO_3$ (20.0 mL). The layers were separated, and then the aqueous layer was extracted with EtOAc (4 × 10.0 mL). The combined organic layers were dried over Na_2SO_4, filtered and concentrated under

reduced pressure. The residue was purified by flash column chromatography on silica gel (20:1 to 10:1 to 3:1, petroleum ether-EtOAc) to furnish compound **48** (35.6 mg, 16% isolated yield from **47**) as a yellow gum and known compound **xi** (95.5 mg, 32% isolated yield from **49**) as a pale yellow oil. Compound **49** (15.1 mg) was recovered in 3.7 % yield and compound **47** (23.9 mg) can be recovered in 24 % yield. The analytical data of **xi** are in agreement with our reported data[2].

Supplementary Table 1. Comparison of ^{1}H NMR data of natural Sarglabolide I[5] (500 MHz) with those of synthetic Sarglabolide I (400 MHz)

Position	Natural, 500 MHz, CD_3OD, δ_H (mult. *J* in Hz)	Synthetic, 400 MHz, CD_3OD, δ_H (mult. *J* in Hz)	$\Delta\delta^*$ (ppm)
1	2.00, m	1.99, m	-0.01
2a	0.32, dd (7.3 , 4.1)	0.32, dd (7.2, 3.6)	0.00
2b	0.98, td (7.8, 4.3)	0.98, td (7.6, 4.1)	0.00
3	1.93, m	1.93, m	0.00
4	--	--	--
5	--	--	--
6	3.94, d (3.3)	3.94, d (3.9)	0.00
7	--	--	--
8	--	--	--
9	4.04, s	4.04, s	0.00
10	--	--	--
11	--	--	--
12	--	--	--
13	1.85, s	1.85, s	0.00
14	1.03, s	1.01, s	-0.02
15a	2.53, ddd (16.4, 6.0, 4.2)	2.53, ddd (16.4, 6.2, 4.0)	0.00
15b	2.83, br. d (16.4)	2.84, br. d (16.3)	0.01
1′	1.61, td (8.2, 4.1)	1.60, td (8.3, 3.9)	-0.01

2´a	0.68, td (8.3, 5.5)	0.68, td (8.7, 5.4)	0.00
2´b	1.22, m	1.22, dt (5.6, 3.9)	0.00
3´	1.53, td (8.8, 3.6)	1.53, m	0.00
4´	--	--	--
5´	1.71, dd (13.8, 6.0)	1.70, dd (13.8, 5.9)	-0.01
6´a	2.24, dd (18.3, 6.0)	2.24, dd (18.2, 6.0)	0.00
6´b	2.78, dd (18.3, 13.9)	2.77, dd (18.4, 14.0)	-0.01
7´	--	--	--
8´	--	--	--
9´	1.82, dd (6.1, 1.2)	1.81, dd (6.1, 1.8)	-0.01
10´	--	--	--
11´	--	--	--
12´	--	--	--
13´a	4.24, d (13.2)	4.23, d (13.2)	-0.01
13´b	4.26, d (13.2)	4.26, d (13.2)	0.00
14´	0.91, s	0.91, s	0.00
15´a	3.31, d (10.9)	3.30, d (10.9)	-0.01
15´b	3.35, d (10.9)	3.35, d (10.9)	0.00
12-O*Me*	3.79, s	3.77, s	-0.02

*The chemical shift of synthetic product minus the chemical shift of Natural product.

Supplementary Table 2. Comparison of ^{13}C NMR data of natural Sarglabolide I[5] (125 MHz) with those of synthetic Sarglabolide I (100 MHz)

Position	Natural, 150 MHz, CD_3OD, δ_C (ppm)	Synthetic, 100 MHz, CD_3OD, δ_C (ppm)	$\Delta\delta^*$ (ppm)
1	26.3	26.3	0.0
2	16.1	16.1	0.0
3	25.4	25.4	0.0
4	143.2	143.2	0.0
5	133.8	133.7	-0.1
6	42.0	41.9	-0.1
7	134.4	134.4	0.0
8	202.2	202.1	-0.1
9	81.2	81.2	0.0
10	52.5	52.6	0.1
11	147.1	147.1	0.0
12	173.1	173.1	0.0
13	20.4	20.5	0.1
14	15.8	15.8	0.0
15	26.0	26.0	0.0
1′	26.6	26.5	-0.1
2′	12.7	12.7	0.0
3′	29.1	29.1	0.0

4′	79.5	79.4	-0.1
5′	60.5	60.6	0.1
6′	23.1	23.0	-0.1
7′	174.2	174.2	0.0
8′	94.7	94.7	0.0
9′	57.3	57.3	0.0
10′	45.8	45.8	0.1
11′	128.6	128.5	-0.1
12′	171.5	171.6	0.1
13′	54.4	54.3	-0.1
14′	27.1	27.1	0.0
15′	69.3	69.2	-0.1
12-O*Me*	53.0	53.0	0.0

*The chemical shift of synthetic product minus the chemical shift of Natural product.

Supplementary Table 3. Comparison of ^{1}H NMR data of natural Multistalide B[6] (400 MHz) with those of synthetic Multistalide B (800 MHz)

Position	Natural, 400 MHz, $CDCl_3$, δ_H (mult. J in Hz)	Synthetic, 800 MHz, $CDCl_3$, δ_H (mult. J in Hz)	$\Delta\delta^*$ (ppm)
1	2.08, m	2.07, dt (9.1, 4.7)	-0.01
2a	0.99, m	0.99, m	0.00
2b	0.32, m	0.32, q (4.0)	0.00
3	1.83, m	1.84, m	0.01
4	--	--	--
5	--	--	--
6	3.92, s	3.93, d (3.9)	0.01
7	--	--	--
8	--	--	--
9	3.94, s	3.94, s	0.00
10	--	--	--
11	--	--	--
12	--	--	--
13	1.94, s	1.94, s	0.00
14	1.01, s	1.01, s	0.00
15a	2.81, dd (16.4, 1.3)	2.81, d (8.0)	0.00
15b	2.58, m	2.58, dt (16.3, 5.2)	0.00
1′	1.62, m	1.62, td (8.1, 4.1)	0.00

2´a	0.72, m	0.72, td (8.8, 5.8)	0.00
2´b	1.27, m	1.27, d (4.4)	0.00
3´	1.47, m	1.46, td (8.4, 3.6)	-0.01
4´	--	--	--
5´	1.86, m	1.86, m	0.00
6´a	2.32, dd (18.3, 6.1)	2.32, dd (18.3, 6.0)	0.00
6´b	2.70, dd (18.3, 13.5)	2.69, dd (18.3, 13.7)	-0.01
7´	--	--	--
8´	--	--	--
9´	1.92, m	1.92, d (1.8)	0.00
10´	--	--	--
11´	--	--	--
12´	--	--	--
13´a	4.42, d (13.5)	4.42, d (12.7)	0.00
13´b	4.34, d (13.5)	4.35, d (14.2)	0.01
14´	0.88, s	0.88, s	0.00
15´a	4.08, d (11.6)	4.08, d (11.6)	0.00
15´b	3.82, d (11.6)	3.82, d (11.7)	0.00
a	--	--	--
b	2.13, s	2.13, s	0.00
12-O*Me*	3.80, s	3.80, s	0.00

*The chemical shift of synthetic product minus the chemical shift of Natural product.

Supplementary Table 4. Comparison of ^{13}C NMR data of natural Multistalide B[6] (100 MHz) with those of synthetic Multistalide B (200 MHz)

Position	Natural, 100 MHz, $CDCl_3$, δ_C (ppm)	Synthetic, 200 MHz, $CDCl_3$, δ_C (ppm)	$\Delta\delta^*$ (ppm)
1	25.9	25.7	-0.2
2	16.0	16.1	0.1
3	24.8	25.0	0.2
4	142.5	142.7	0.2
5	132.0	132.2	0.2
6	40.9	41.1	0.2
7	130.9	131.0	0.1
8	200.1	200.2	0.1
9	80.0	80.2	0.2
10	51.1	51.2	0.1
11	147.6	147.7	0.1
12	171.0	171.1	0.1
13	20.5	20.7	0.2
14	15.4	15.6	0.2
15	25.2	25.4	0.2
1′	25.5	25.7	0.2
2′	11.7	11.9	0.2
3′	28.2	28.4	0.2

4′	77.3	77.3	0.0
5′	60.0	60.2	0.2
6′	22.3	22.5	0.2
7′	168.2	168.3	0.1
8′	93.4	93.6	0.2
9′	55.0	55.2	0.2
10′	44.8	45.0	0.2
11′	127.2	127.4	0.2
12′	172.4	172.5	0.1
13′	54.9	55.1	0.2
14′	26.2	26.4	0.2
15′	71.5	71.6	0.1
a	171.3	171.4	0.1
b	20.8	21.0	0.2
12-O*Me*	53.0	53.0	0.0

*The chemical shift of synthetic product minus the chemical shift of Natural product.

Supplementary Table 5. Comparison of ^{1}H NMR data of natural Shizulaol C[7] (500 MHz) with those of synthetic Shizulaol C (400 MHz)

Position	Natural, 500 MHz, pyridine-d_5, δ_H (mult. J in Hz)	Synthetic, 400 MHz, pyridine-d_5, δ_H (mult. J in Hz)	$\Delta\delta^*$ (ppm)
1	2.35, ddd (8.0, 7.7, 4.3)	2.36, ddd (8.2, 5.8, 4.1)	0.01
2a	0.99, ddd (7.7, 7.6, 4.3)	0.99, td (7.8, 4.2)	0.00
2b	0.40, ddd (4.3, 4.3, 3.1)	0.40, q (3.8)	0.00
3	1.97, m	1.97, ddd (13.1, 6.3, 2.3)	0.00
4	--	--	--
5	--	--	--
6	4.24, d (3.5)	4.25, d (3.7)	0.01
7	--	--	--
8	--	--	--
9	4.44, s	4.45, s	0.01
10	--	--	--
11	--	--	--
12	--	--	--
13	2.17, s	2.18, s	0.01
14	1.35, s	1.35, s	0.00
15a	2.83, dd (16.3, 1.5)	2.84, dd (16.2, 1.8)	0.01
15b	2.69, ddd (16.3, 6.1, 3.5)	2.69, dt (16.3, 4.6)	0.00
1′	1.72, ddd (8.5, 7.6, 4.1)	1.73, td (8.0, 4.2)	0.01

2′a	0.77, ddd (8.8, 8.5, 5.8)	0.77, ddd (9.2, 9.0, 6.4)	0.00
2′b	1.55, complex	1.55, m	0.00
3′	1.55, ddd (8.8, 7.6, 3.5)	1.55, m	0.00
4′	--	--	--
5′	2.15, dd (13.7, 6.1)	2.15, m	0.00
6′a	2.98, dd (18.3, 6.1)	2.99, dd (18.1, 6.0)	0.01
6′b	3.47, dd (18.3, 13.7)	2.45, d (10.1)	-0.01
7′	--	--	--
8′	--	--	--
9′	1.98, dd (6.1, 1.5)	1.98, td (6.0, 2.2)	0.00
10′	--	--	--
11′	--	--	--
12′	--	--	--
13′a	4.76, d (13.7)	4.76, d (12.9)	0.00
13′b	4.80, d (13.7)	4.81, d (13.0)	0.01
14′	1.20, s	1.21, s	0.01
15′a	4.29, d (11.6)	4.30, d (11.4)	0.01
15′b	4.56, d (11.6)	4.57, d (11.3)	0.01
a	--	--	--
b	--	--	--
c	6.92, qq (7.1, 1.0)	6.92 qd (6.8, 3.4)	0.00
d	1.58, d (7.1)	1.58, d (6.0)	0.00

e	1.81, br. s	1.81, d (1.4)	0.00
12-O*Me*	3.69, s	3.70, s	0.01

*The chemical shift of synthetic product minus the chemical shift of Natural product.

Supplementary Table 6. Comparison of ^{13}C NMR data of natural Shizulaol C [7] (125 MHz) with those of synthetic Shizulaol C (200 MHz)

Position	Natural, 125 MHz, $CDCl_3$, δ_C (ppm)	Synthetic, 200 MHz, $CDCl_3$, δ_C (ppm)	$\Delta\delta^*$ (ppm)
1	26.1	26.3	0.2
2	16.0	16.2	0.2
3	24.9	25.0	0.1
4	142.7	142.8	0.1
5	132.3	132.4	0.1
6	41.2	41.3	0.1
7	130.8	130.9	0.1
8	200.0	200.1	0.1
9	80.0	80.1	0.1
10	51.1	51.2	0.1
11	147.5	147.6	0.1
12	171.0	171.2	0.2
13	20.4	20.6	0.2
14	15.6	15.7	0.1
15	25.3	25.4	0.1
1′	25.5	25.7	0.2
2′	11.8	11.9	0.1
3′	28.4	28.5	0.1

4′	77.6	77.7	0.1
5′	59.9	60.0	0.1
6′	22.2	22.4	0.2
7′	168.3	168.4	0.1
8′	93.6	93.7	0.1
9′	54.8	55.0	0.2
10′	44.9	45.0	0.1
11′	127.4	127.6	0.2
12′	172.3	172.4	0.1
13′	54.9	55.1	0.2
14′	26.1	26.3	0.2
15′	71.0	71.2	0.2
a	168.2	168.4	0.2
b	128.0	128.1	0.1
c	138.9	139.1	0.2
d	14.2	14.3	0.1
e	12.1	12.3	0.2
12-O*Me*	52.7	52.9	0.2

*The chemical shift of synthetic product minus the chemical shift of Natural product.

Supplementary Table 7. Comparison of ^{1}H NMR data of natural Shizulaol I[9] (500 MHz) with those of synthetic Shizulaol I (800 MHz)

Position	Natural, 500 MHz, $CDCl_3$, δ_H (mult. J in Hz)	Synthetic, 800 MHz, $CDCl_3$, δ_H (mult. J in Hz)	$\Delta\delta^*$ (ppm)
1	2.08, ddd (8.2, 5.9, 4.2)	2.08, dd (8.8, 4.8)	0.00
2a	1.01, ddd (8.2, 7.4, 4.1)	1.01, m	0.00
2b	0.33, ddd (4.2, 4.1, 3.2)	0.33, dd (7.3, 4.0)	0.00
3	1.87, ddd (7.4, 5.9, 3.2)	1.87, ddd (5.6, 4.8, 3.2)	0.00
4	--	--	--
5	--	--	--
6	3.93, br. d (4.0)	3.93, br. d (3.3)	0.00
7	--	--	--
8	--	--	--
9	4.00, s	4.01, s	0.01
10	--	--	--
11	--	--	--
12	--	--	--
13	1.92, s	1.93, s	0.01
14	1.00, s	0.99, s	-0.01
15a	2.82, dd (16.3, 1.5)	2.83, d (16.4)	0.01
15b	2.57, ddd (16.3, 6.2, 4.0)	2.57, m	0.00
1′	1.63, ddd (8.4, 7.8, 3.9)	1.64, ddd (8.0, 7.2, 4.0)	0.01

2′a	0.71, ddd (9.1, 8.4, 5.7)	0.71, td (8.7, 5.9)	0.00
2′b	1.28, ddd (5.7, 3.9, 3.6)	1.28, m	0.00
3′	1.47, ddd (9.1, 7.8, 3.5)	1.47, ddd (8.8, 7.2, 3.2)	0.00
4′	--	--	--
5′	1.92, dd (13.6, 6.0)	1.94, dd (14.4, 6.4)	0.02
6′a	2.32, br. dd (18.3, 6.0)	2.30, dd, (18.2, 5.9)	-0.02
6′b	2.72, dd (18.3, 13.6)	2.68, dd (18.2, 13.6)	-0.04
7′	--	--	--
8′	--	--	--
9′	1.92, dd (6.2, 1.5)	1.94, d (6.4)	0.02
10′	--	--	--
11′	--	--	--
12′	--	--	--
13′a	4.33, d (13.1)	4.33, d (13.5)	0.00
13′b	4.40, d (13.1)	4.40, d (13.5)	0.00
14′	0.87, s	0.87, s	0.00
15′a	3.81, d (11.8)	3.81, d (11.9)	0.00
15′b	4.26, d (11.8)	4.30, d (11.8)	0.04
a	--	--	--
b	6.01, q (1.0)	6.00 br. s	-0.01
c	--	--	--
d	4.18, s	4.18, d (9.3)	0.00

e	2.11, d (1.0)	2.12, s	0.01
12-O*Me*	3.76, s	3.77, s	0.01

*The chemical shift of synthetic product minus the chemical shift of Natural product.

Supplementary Table 8. Comparison of ^{13}C NMR data of natural Shizulaol I [9] (125 MHz) with those of synthetic Shizulaol I (100 MHz)

Position	Natural, 125 MHz, $CDCl_3$, δ_C (ppm)	Synthetic, 100 MHz, $CDCl_3$, δ_C (ppm)	$\Delta\delta^*$ (ppm)
1	26.0	26.1	0.1
2	16.0	16.1	0.1
3	24.8	25.0	0.2
4	142.5	142.6	0.1
5	132.1	132.2	0.1
6	41.0	41.1	0.1
7	131.2	131.0	-0.2
8	200.0	199.8	-0.2
9	79.7	80.0	0.3
10	51.0	51.2	0.2
11	147.2	147.7	0.5
12	171.6	171.7	0.1
13	20.4	20.6	0.2
14	15.5	15.7	0.2
15	25.2	25.4	0.2
1′	25.6	25.7	0.1
2′	11.7	11.7	0.0
3′	28.3	28.5	0.2

4′	77.4	77.6	0.2
5′	60.1	60.2	0.2
6′	22.6	22.7	0.1
7′	168.6	168.5	-0.1
8′	93.4	93.6	0.2
9′	55.0	55.0	0.0
10′	44.8	45.0	0.2
11′	127.2	127.2	0.0
12′	172.4	172.6	0.2
13′	54.8	55.0	0.2
14′	26.3	26.4	0.1
15′	71.4	71.8	0.4
a	166.8	166.9	0.1
b	112.4	112.4	0.0
c	159.9	160.2	0.3
d	67.1	67.3	0.2
e	15.7	15.9	0.2
12-O*Me*	52.9	53.1	0.2

*The chemical shift of synthetic product minus the chemical shift of Natural product.

Supplementary Table 9. Comparison of ^{1}H NMR data of natural Chlorajaponilide C[10] (500 MHz) with those of synthetic Chlorajaponilide C (400 MHz)

Position	Natural, 500 MHz, $CDCl_3$, δ_H (mult. *J* in Hz)	Synthetic, 400 MHz, $CDCl_3$, δ_H (mult. *J* in Hz)	$\Delta\delta^*$ (ppm)
1	2.06, m	2.06, m	0.00
2a	0.99, m	0.99, m	0.00
2b	0.28, m	0.29, q (4.0)	0.01
3	1.84, m	1.85, br. s	0.01
4	--	--	--
5	--	--	--
6	3.92, d	3.93, d (3.5)	0.01
7	--	--	--
8	--	--	--
9	3.97, s	3.99, s	0.02
10	--	--	--
11	--	--	--
12	--	--	--
13	1.88, s	1.88, s	0.00
14	0.99, s	0.99, s	0.00
15a	2.78, m	2.79, m	0.01
15b	2.60, m	2.60, d (7.8)	0.00
1′	1.59, m	1.60, m	0.01

2′a	0.71, m	0.72, td (8.8, 5.8)	0.01
2′b	1.32, m	1.32, d (5.1)	0.00
3′	1.41, m	1.42, m	0.01
4′	--	--	--
5′	1.86, m	1.87, m	0.01
6′a	2.71, m	2.73, m	0.02
6′b	2.50, dd (18.5, 6.0)	2.51, m	0.01
7′	--	--	--
8′	--	--	--
9′	1.84, m	1.87, br. d	0.01
10′	--	--	--
11′	--	--	--
12′	--	--	--
13′a	4.99, d (13.5)	5.00, d (13.5)	0.01
13′b	4.78, d (13.5)	4.77, d (13.5)	-0.01
14′	0.86, s	0.86, s	0.00
15′a	4.30, d (12.0)	4.32, d (11.8)	0.02
15′b	3.68, d (12.0)	3.69, d (3.6)	0.01
1″	--	--	--
2″	5.97, d (1.5)	5.97, d (1.9)	0.00
3″	--	--	--
4″	4.16, s	4.17, d (6.3)	0.01

5″	2.12, s	2.12,s	0.00
6″	--	--	--
7″	2.66, m	2.66, m	0.00
8″	2.75, m	2.75, m	0.00
12-*COMe*	3.72, s	3.73, s	0.01
6″-COMe	3.66, s	3.66, s	0.00

*The chemical shift of synthetic product minus the chemical shift of Natural product.

Supplementary Table 10. Comparison of ^{13}C NMR data of natural Chlorajaponilide C [10] (125 MHz) with those of synthetic Chlorajaponilide C (200 MHz)

Position	Natural, 125 MHz, $CDCl_3$, δ_C (ppm)	Synthetic, 200 MHz, $CDCl_3$, δ_C (ppm)	$\Delta\delta^*$ (ppm)
1	25.7	25.8	0.1
2	15.8	16.0	0.2
3	24.7	24.9	0.2
4	142.2	142.4	0.2
5	131.7	131.8	0.1
6	40.8	40.9	0.1
7	131.5	131.5	0.0
8	199.8	199.9	0.1
9	80.1	80.2	0.1
10	51.1	51.2	0.0
11	147.3	147.6	0.3
12	171.0	171.2	0.2
13	20.4	20.6	0.2
14	15.2	15.4	0.2
15	25.4	25.6	0.2
1′	25.3	25.4	0.1
2′	11.6	11.7	0.1
3′	27.8	27.9	0.1

4′	77.2	77.4	0.2
5′	60.9	61.1	0.2
6′	23.4	23.7	0.3
7′	171.3	171.5	0.2
8′	93.3	93.4	0.1
9′	55.7	55.9	0.2
10′	44.8	44.9	0.1
11′	123.4	123.5	0.1
12′	172.1	172.3	0.2
13′	55.7	55.8	0.1
14′	26.5	26.6	0.1
15′	71.8	71.9	0.1
1″	166.6	166.7	0.1
2″	112.5	112.6	0.1
3″	159.5	159.7	0.2
4″	67.2	67.3	0.1
5″	15.7	15.8	0.1
6″	173.1	173.3	0.2
7″	28.6	28.7	0.1
8″	28.7	28.8	0.1
9″	172.0	172.1	0.1
12- CO*Me*	52.7	52.9	0.2

6″-CO*Me*	52.0	52.2	0.2

*The chemical shift of synthetic product minus the chemical shift of Natural product.

Supplementary Table 11. Comparison of ^{1}H NMR data of natural Shizukaol A[12] (500 MHz) with those of synthetic Shizukaol A (400 MHz)

Position	Natural, 500 MHz, $CDCl_3$, δ_H (mult. J in Hz)	Synthetic, 400 MHz, $CDCl_3$, δ_H (mult. J in Hz)	$\Delta\delta^*$ (ppm)
1	2.03, ddd (7.7, 5.6, 4.3)	2.02, ddd (8.2, 5.7, 4.2)	-0.01
2a	0.99, ddd (7.8, 7.7, 4.3)	0.97, m	-0.02
2b	0.30, ddd (4.3, 4.3, 3.4)	0.30, td (4.3, 2.9)	0.00
3	1.86, ddd (7.8, 5.6, 3.4)	1.86, br. dd (6.8, 2.0)	0.00
4	--	--	--
5	--	--	--
6	3.88, d (3.4)	3.88, d (4.2)	0.00
7	--	--	--
8	--	--	--
9	3.87, s	3.87, s	0.00
10	--	--	--
11	--	--	--
12	--	--	--
13	1.82, s	1.81, s	-0.01
14	1.01, s	1.01, s	0.00
15a	2.77, dd (16.2, 1.7)	2.77, dd (16.2, 1.7)	0.00
15b	2.63, ddd (16.2, 5.6, 3.4)	2.63, ddd (16.3, 5.6, 3.8)	0.00
1′	1.57, ddd (7.8, 7.3, 3.4)	1.56, ddd (7.8, 7.4, 3.9)	-0.01

2´a	0.82, ddd (7.8, 7.8, 4.6)	0.82, td (8.6, 5.4)	0.00
2´b	0.76, ddd (4.6, 4.6, 3.4)	0.76, dt (5.4, 3.7)	0.00
3´	1.86, ddd (7.8, 7.3, 4.6)	1.86, br. dd (6.8, 2.0)	0.00
4´	--	--	--
5´	2.46, dd (13.3, 6.0)	2.46, m	0.00
6´a	2.27, dd (17.5, 6.0)	2.27, m	0.00
6´b	2.21, ddq (17.5, 13.3, 1.7)	2.21, dd (18.8, 6.0)	0.00
7´	--	--	--
8´	--	--	--
9´	1.91, dd (5.6, 1.7)	1.91, dd (5.8, 2.0)	0.00
10´	--	--	--
11´	--	--	--
12´	--	--	--
13´	1.82, s	1.81, s	-0.01
14´	0.51, s	0.51, s	0.00
15´a	4.58, m	4.58, t (2.2)	0.00
15´b	4.91, m	4.91, t (1.9)	0.00
12-*COMe*	3.80, s	3.80, s	0.00

*The chemical shift of synthetic product minus the chemical shift of Natural product.

Supplementary Table 12. Comparison of ^{13}C NMR data of natural Shizukaol A[12] (125 MHz) with those of synthetic Shizukaol A (100 MHz)

Position	Natural, 125 MHz, $CDCl_3$, δ_C (ppm)	Synthetic, 100 MHz, $CDCl_3$, δ_C (ppm)	$\Delta\delta^*$ (ppm)
1	25.7	25.9	0.2
2	15.8	16.0	0.2
3	24.9	25.0	0.1
4	142.2	142.4	0.2
5	132.1	132.0	-0.1
6	41.0	41.0	0.0
7	132.0	132.0	0.0
8	200.1	200.2	0.1
9	79.7	79.7	0.0
10	51.2	51.2	0.0
11	146.8	147.0	0.2
12	170.9	171.1	0.2
13	20.1	20.3	0.2
14	15.3	15.5	0.2
15	25.6	25.7	0.1
1′	23.7	23.8	0.1
2′	16.5	16.7	0.2
3′	22.9	23.0	0.1

4′	149.0	149.0	0.0
5′	59.8	59.9	0.1
6′	23.1	23.2	0.1
7′	165.2	165.4	0.2
8′	93.0	93.1	0.1
9′	54.5	54.5	0.0
10′	41.9	41.9	0.0
11′	124.4	124.5	0.1
12′	173.5	173.7	0.2
13′	8.6	8.8	0.2
14′	25.0	25.1	0.1
15′	107.2	107..4	0.2
12-CO*Me*	52.6	52.8	0.2

*The chemical shift of synthetic product minus the chemical shift of Natural product.

Spectra for Compounds

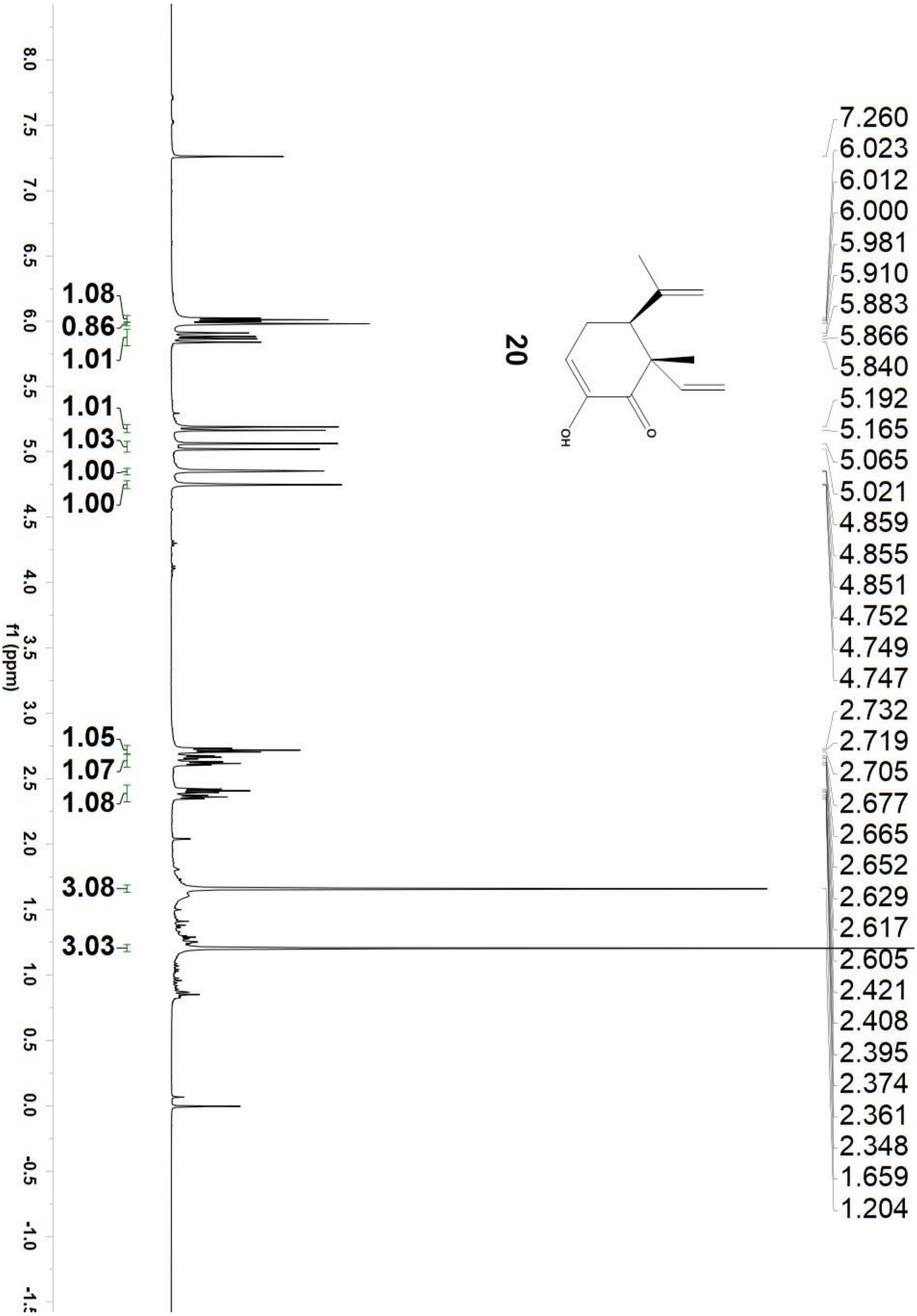

Supplementary Figure 26. ^{1}H NMR spectra of compound **20** in $CDCl_3$ (400 MHz)

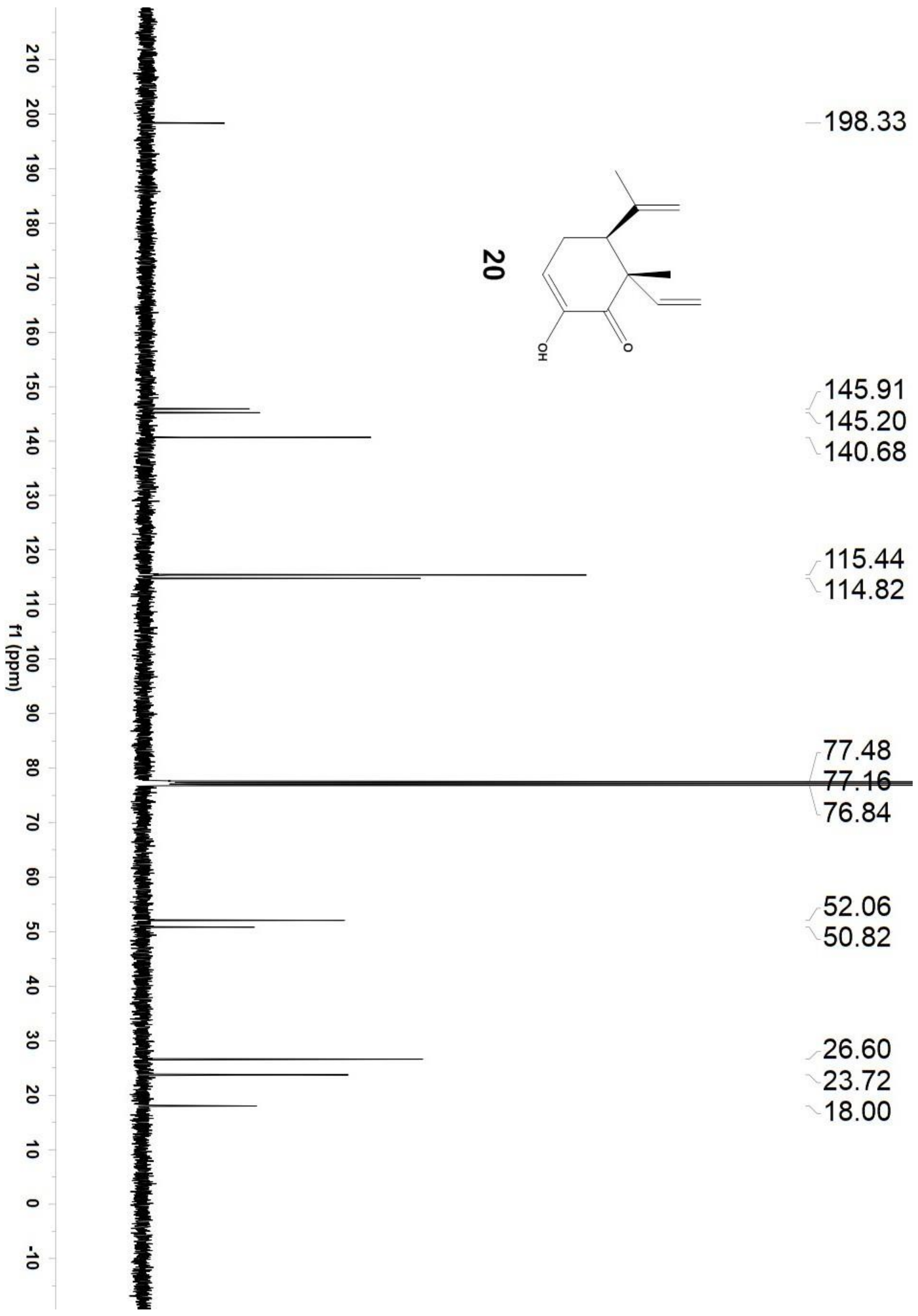

Supplementary Figure 27. ^{13}C NMR spectra of compound **20** in $CDCl_3$ (100 MHz)

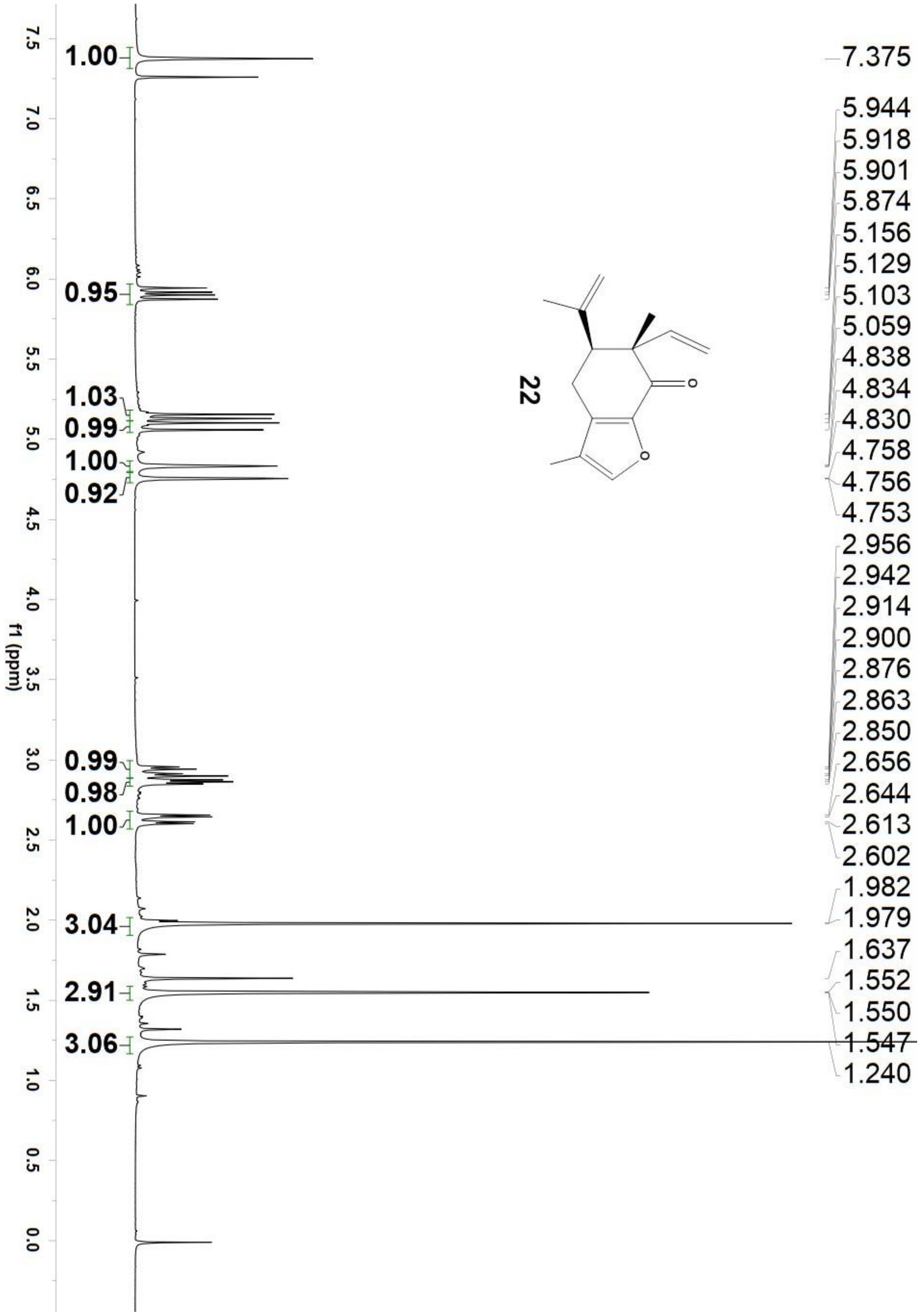

Supplementary Figure 28. ^{1}H NMR spectra of compound **22** in $CDCl_3$ (400 MHz)

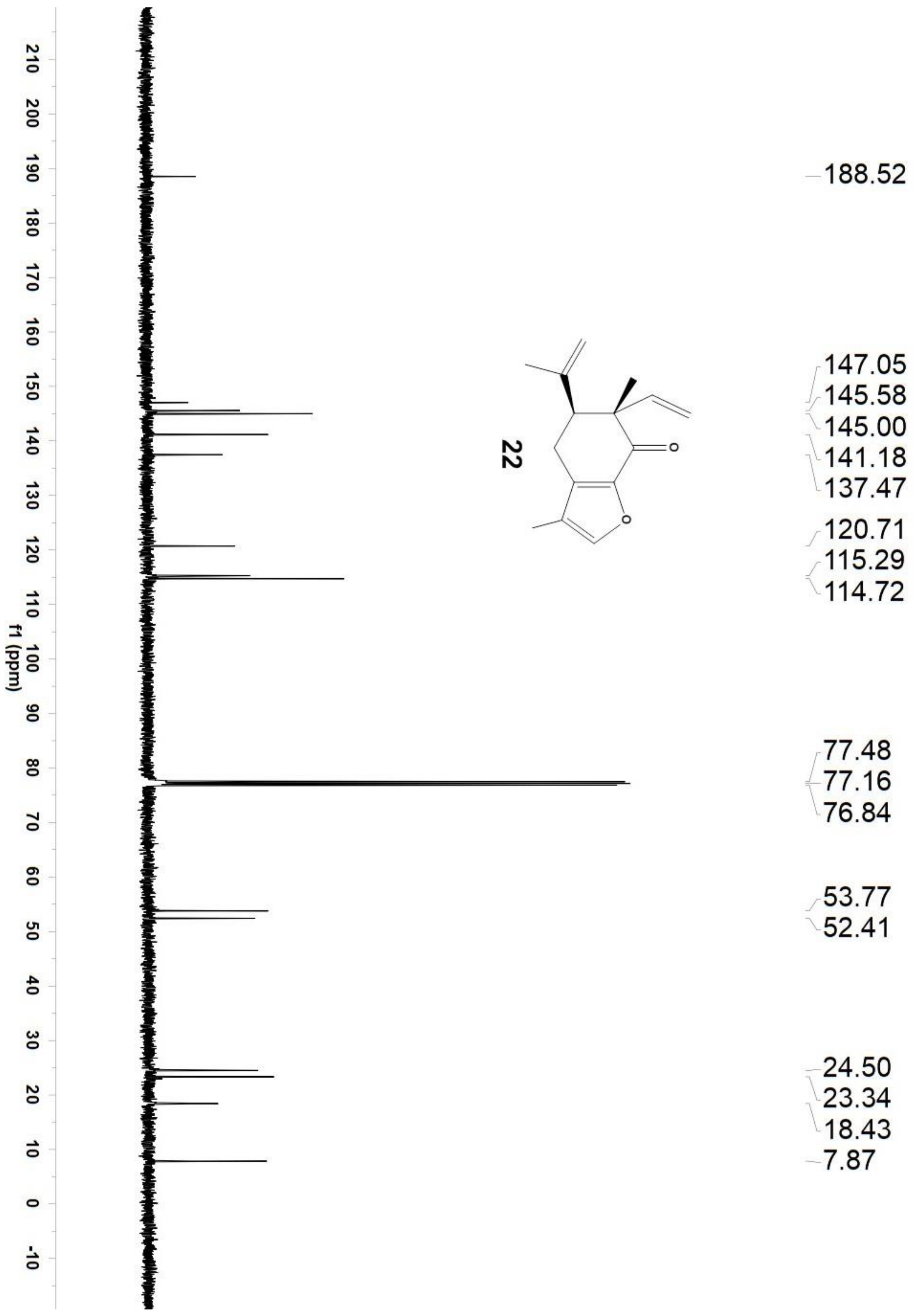

Supplementary Figure 29. ^{13}C NMR spectra of compound 22 in $CDCl_3$ (100 MHz)

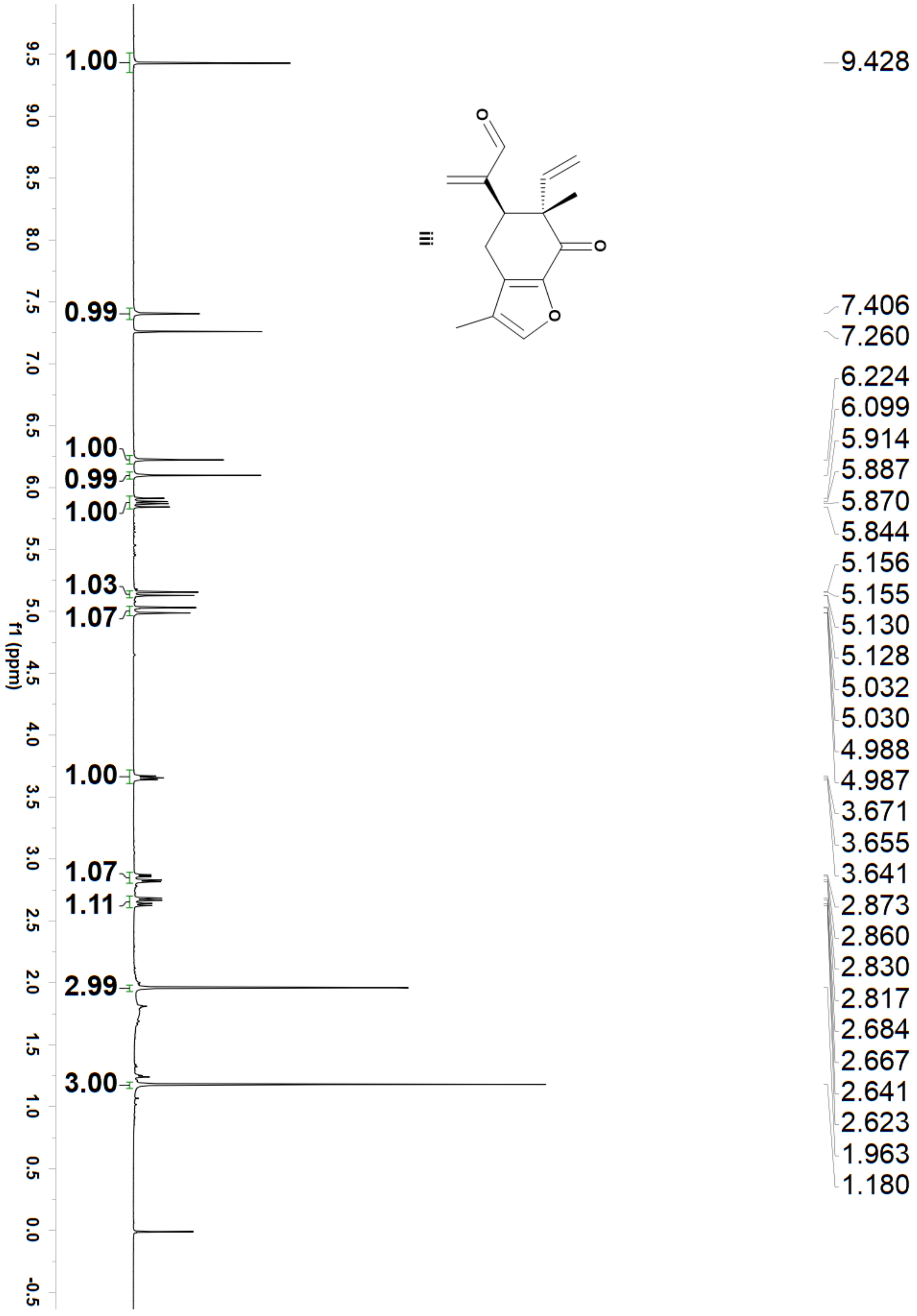

Supplementary Figure 30. ^{1}H NMR spectra of compound **iii** in $CDCl_3$ (400 MHz)

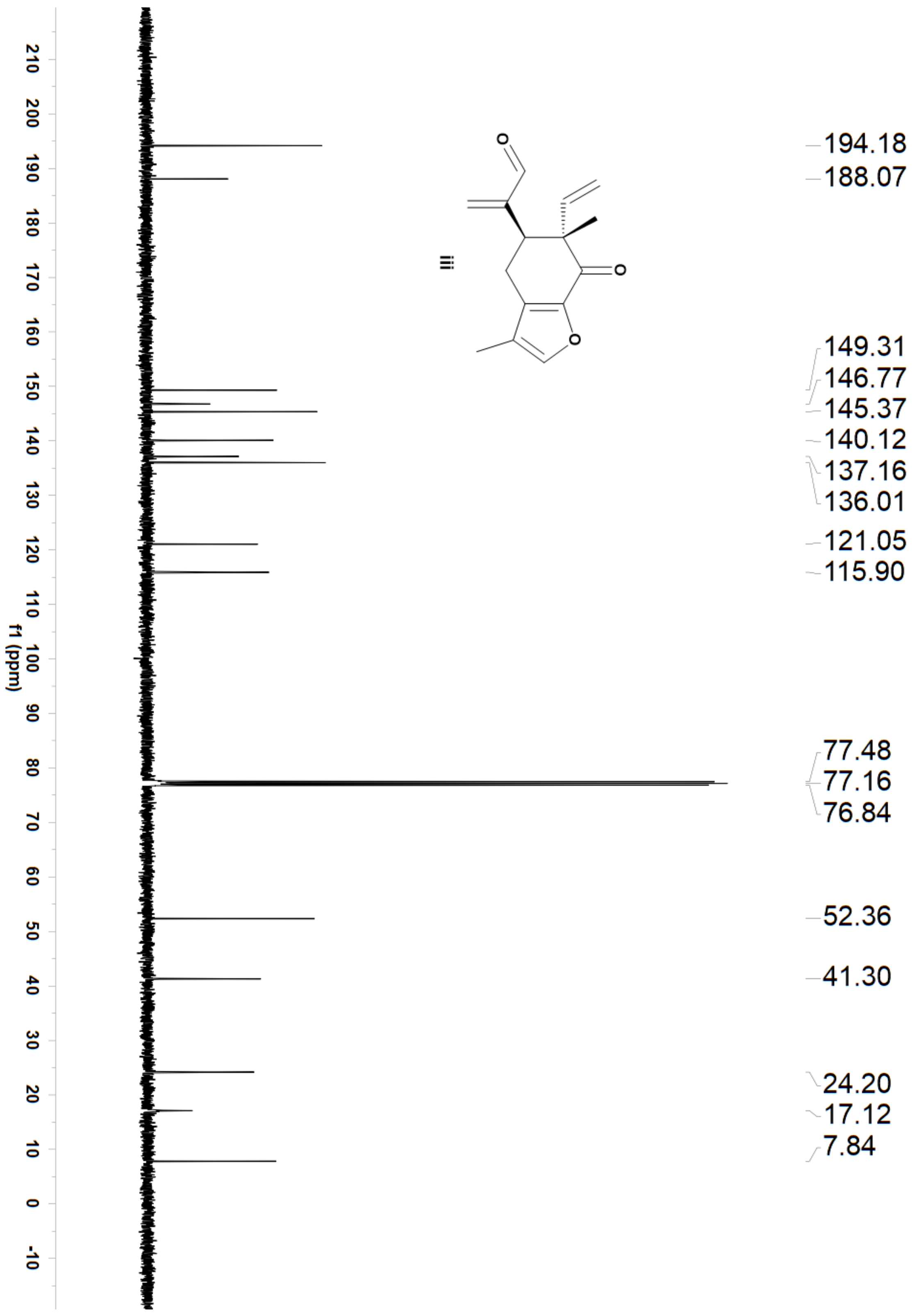

Supplementary Figure 31. ^{13}C NMR spectra of compound **iii** in $CDCl_3$ (100 MHz)

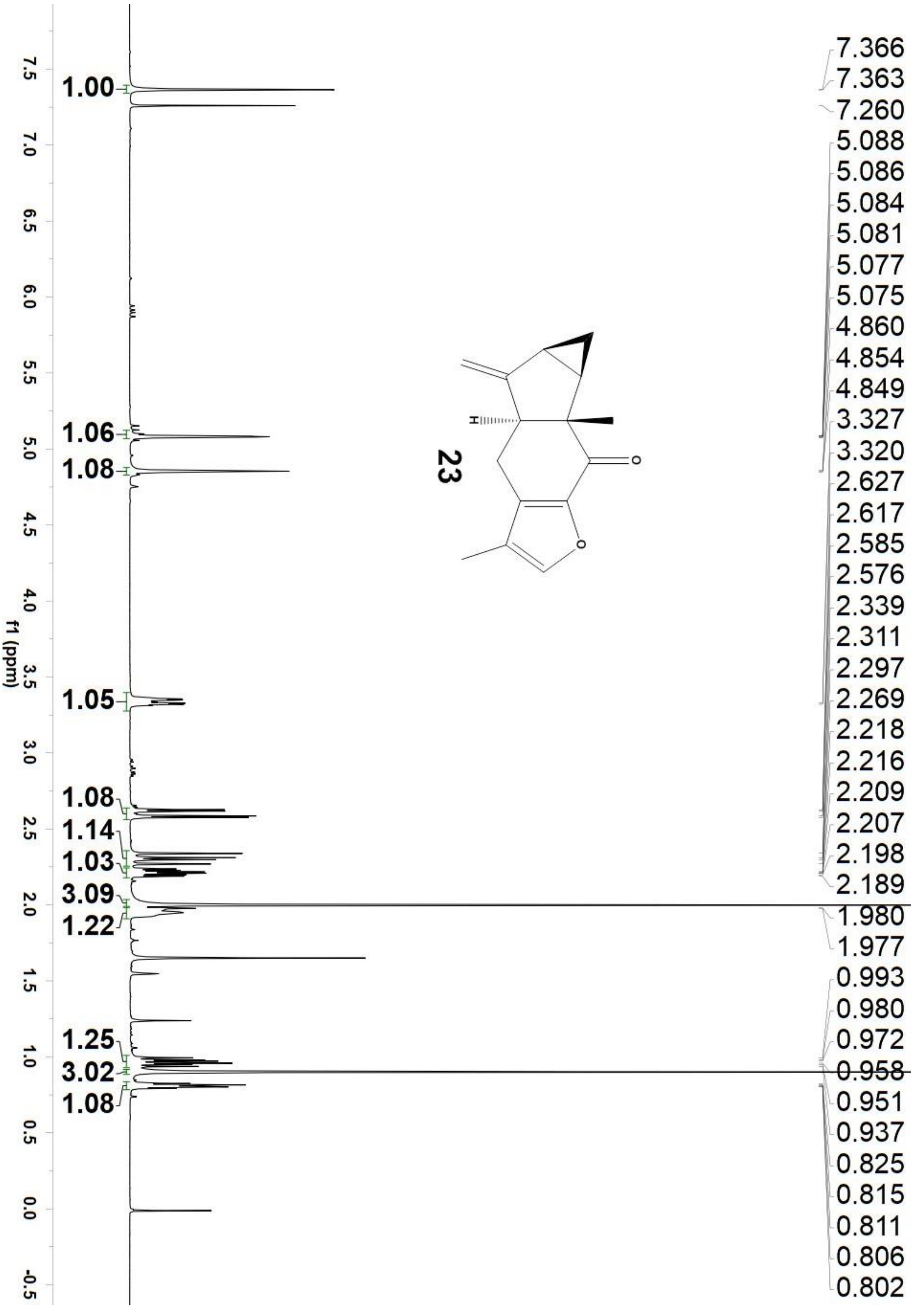

Supplementary Figure 32. ^{1}H NMR spectra of compound **23** in $CDCl_3$ (400 MHz)

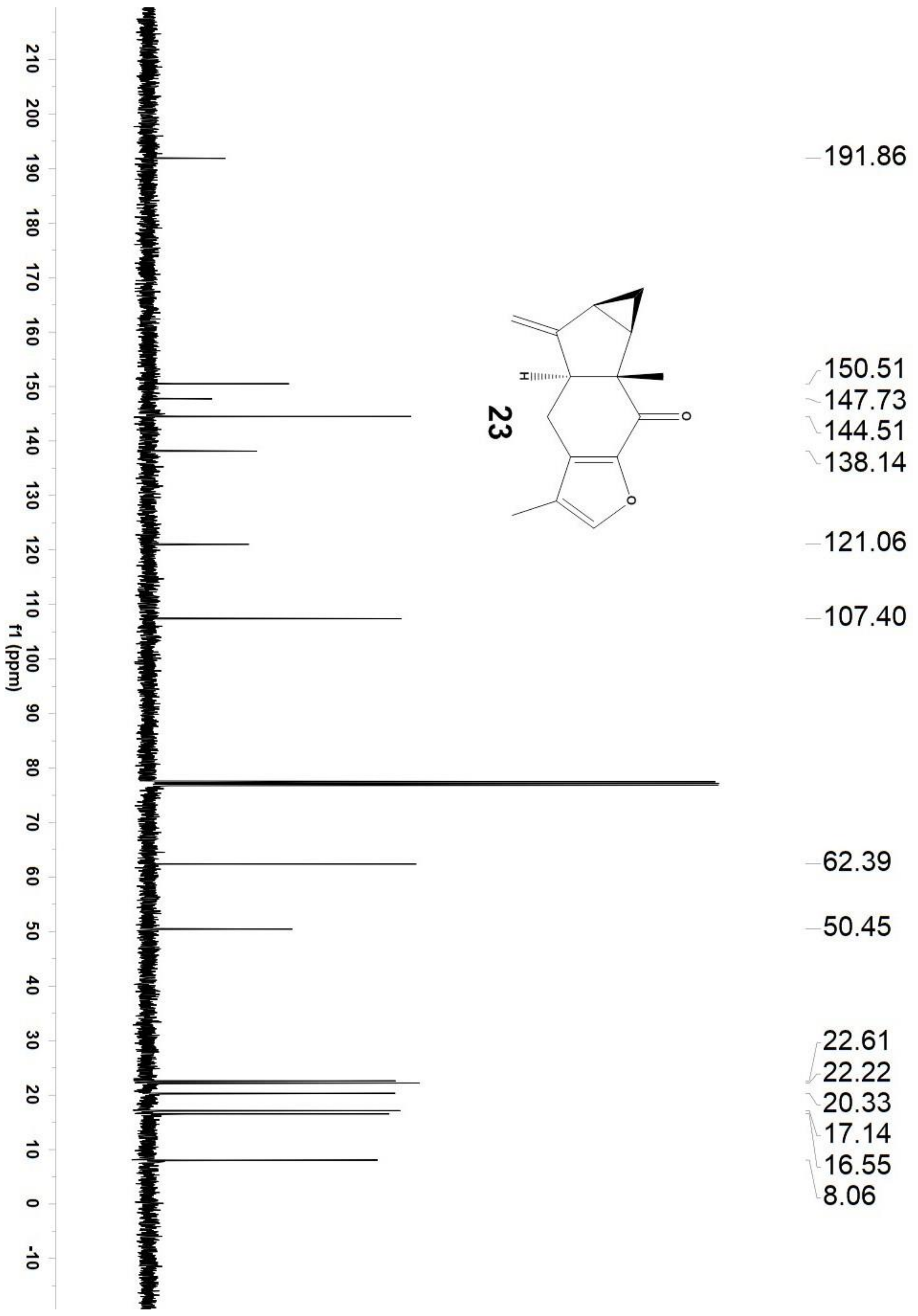

Supplementary Figure 33. ^{13}C NMR spectra of compound **23** in $CDCl_3$ (100 MHz)

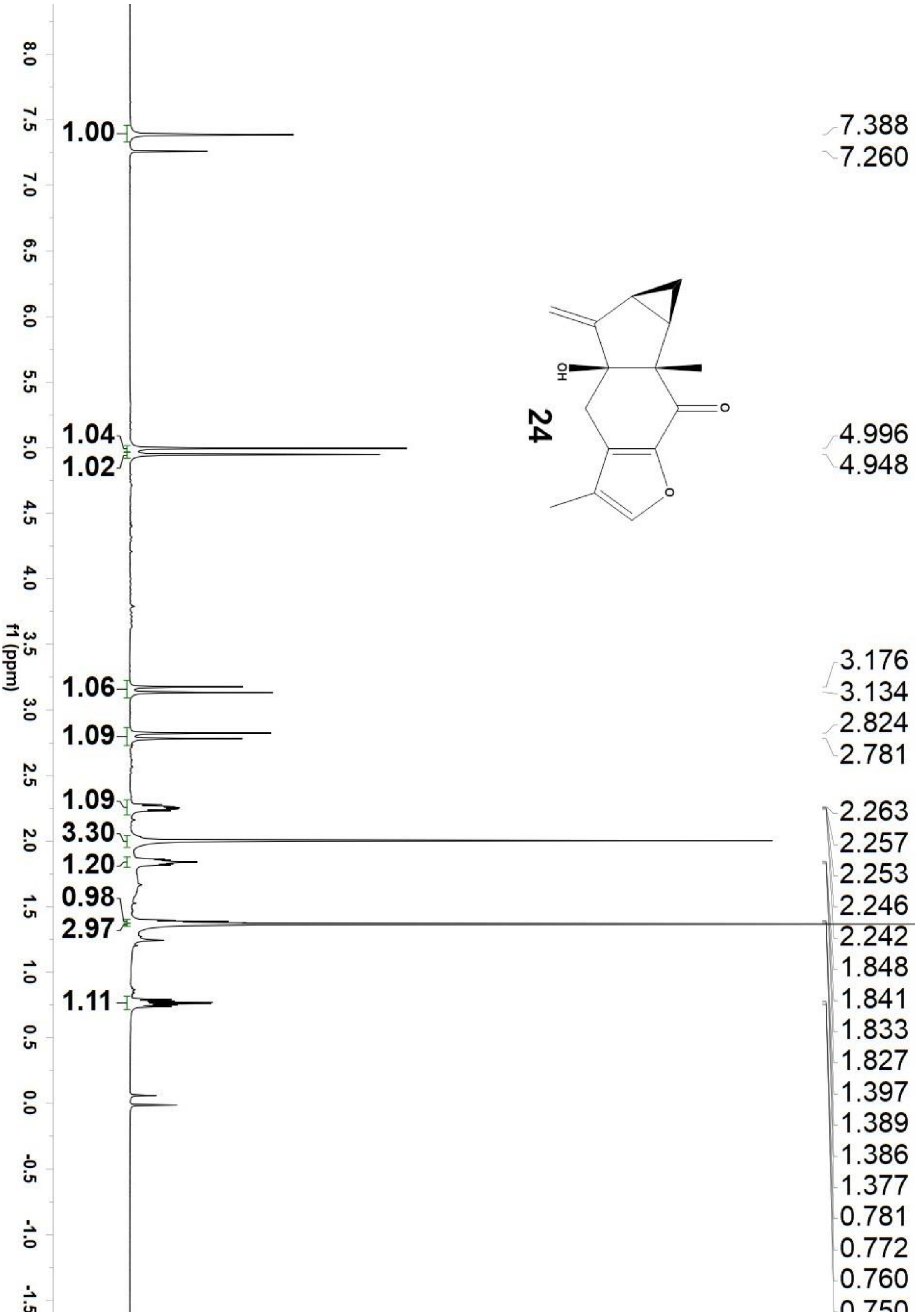

Supplementary Figure 34. ^{1}H NMR spectra of compound **24** in $CDCl_3$ (400 MHz)

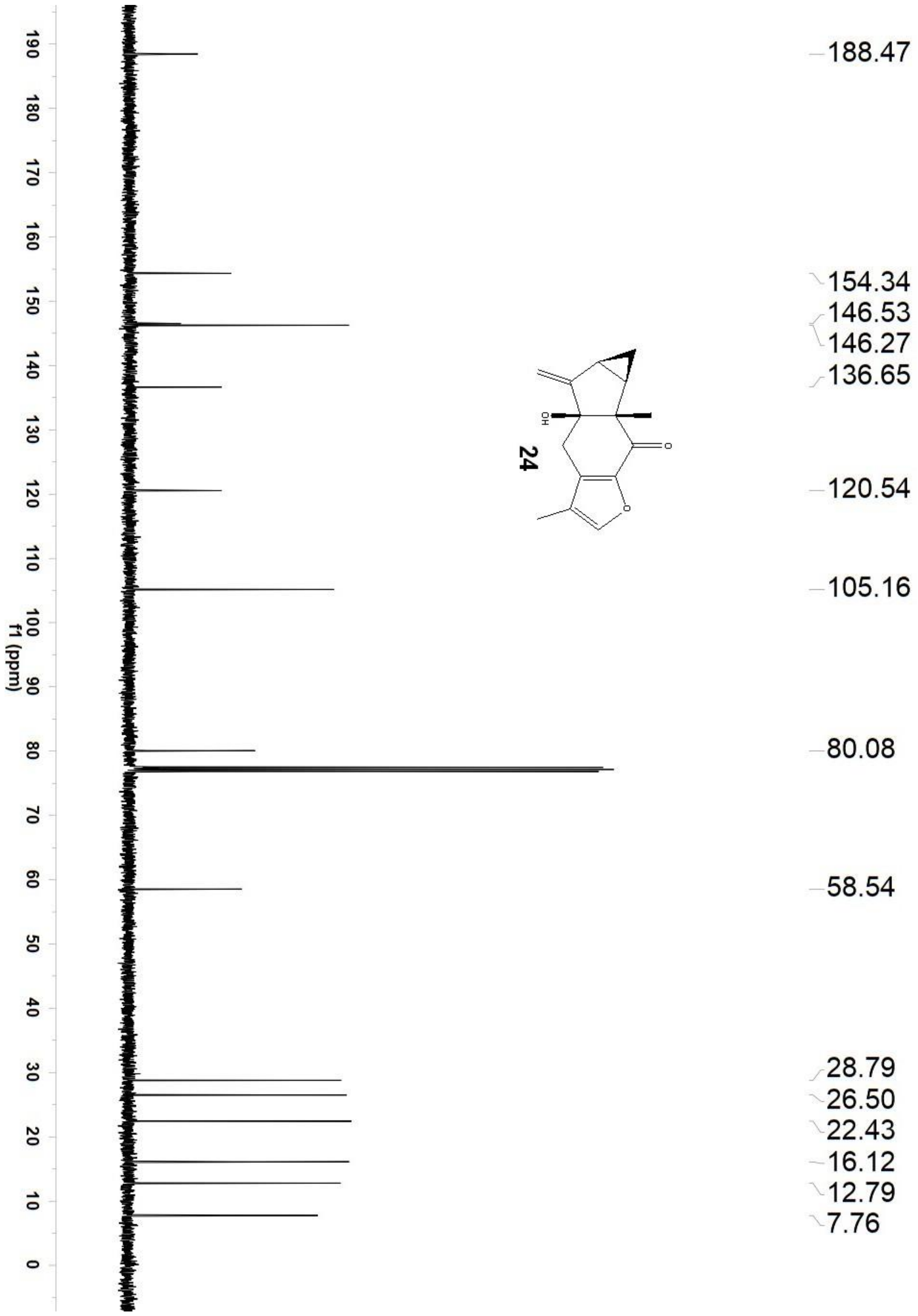

Supplementary Figure 35. ^{13}C NMR spectra of compound **24** in $CDCl_3$ (100 MHz)

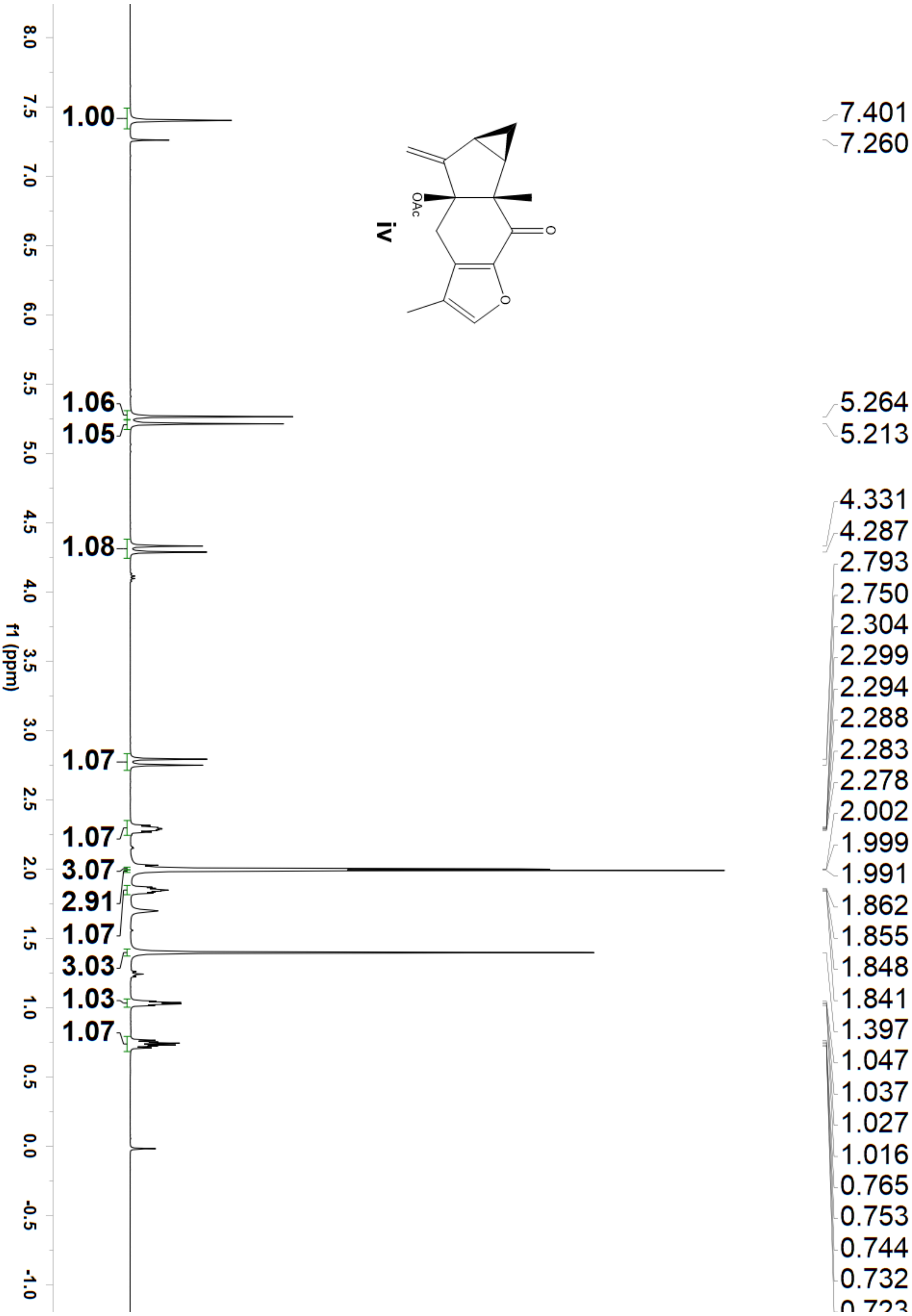

Supplementary Figure 36. ^{1}H NMR spectra of compound **iv** in $CDCl_3$ (400 MHz)

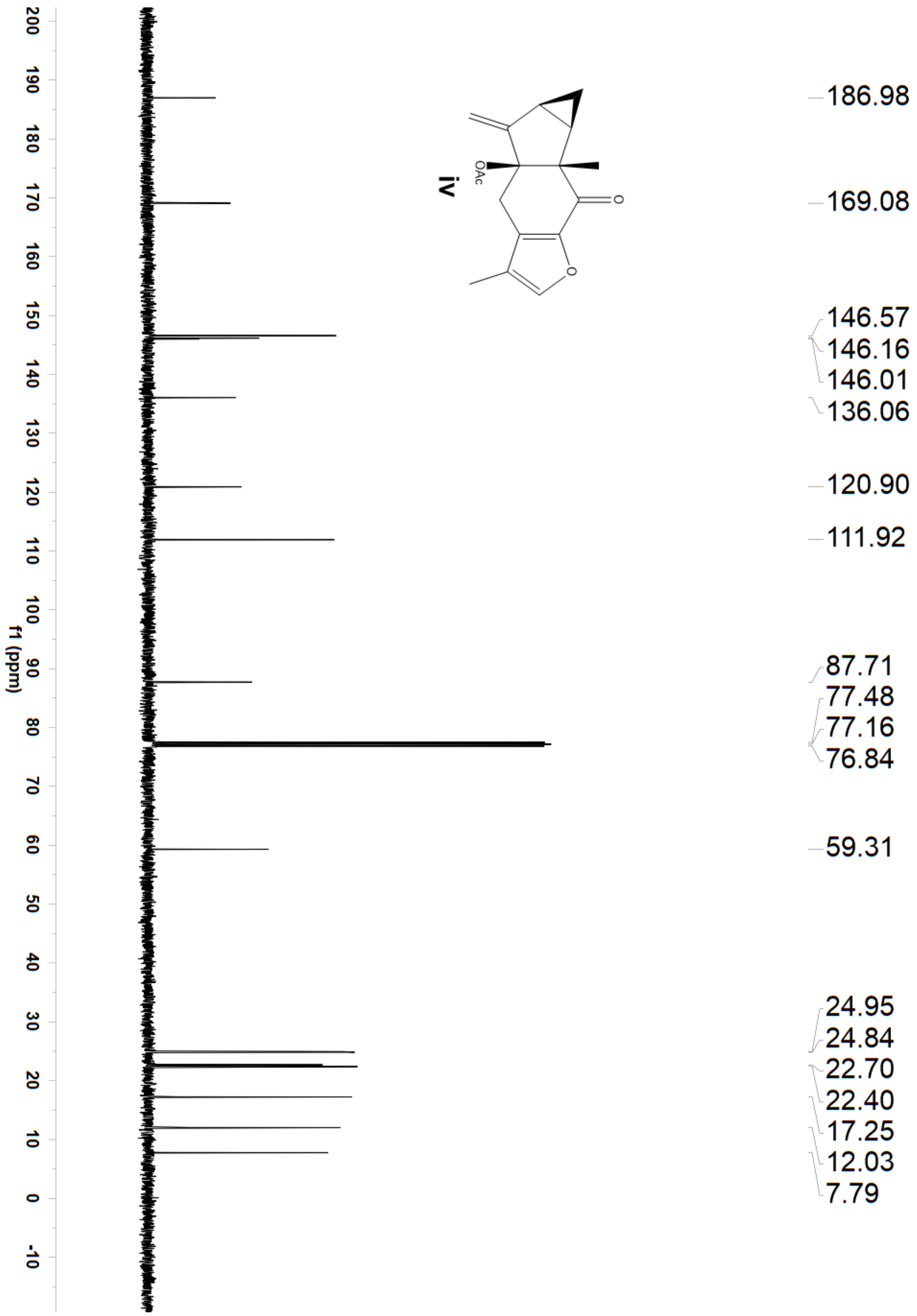

Supplementary Figure 37. ^{13}C NMR spectra of compound **iv** in $CDCl_3$ (100 MHz)

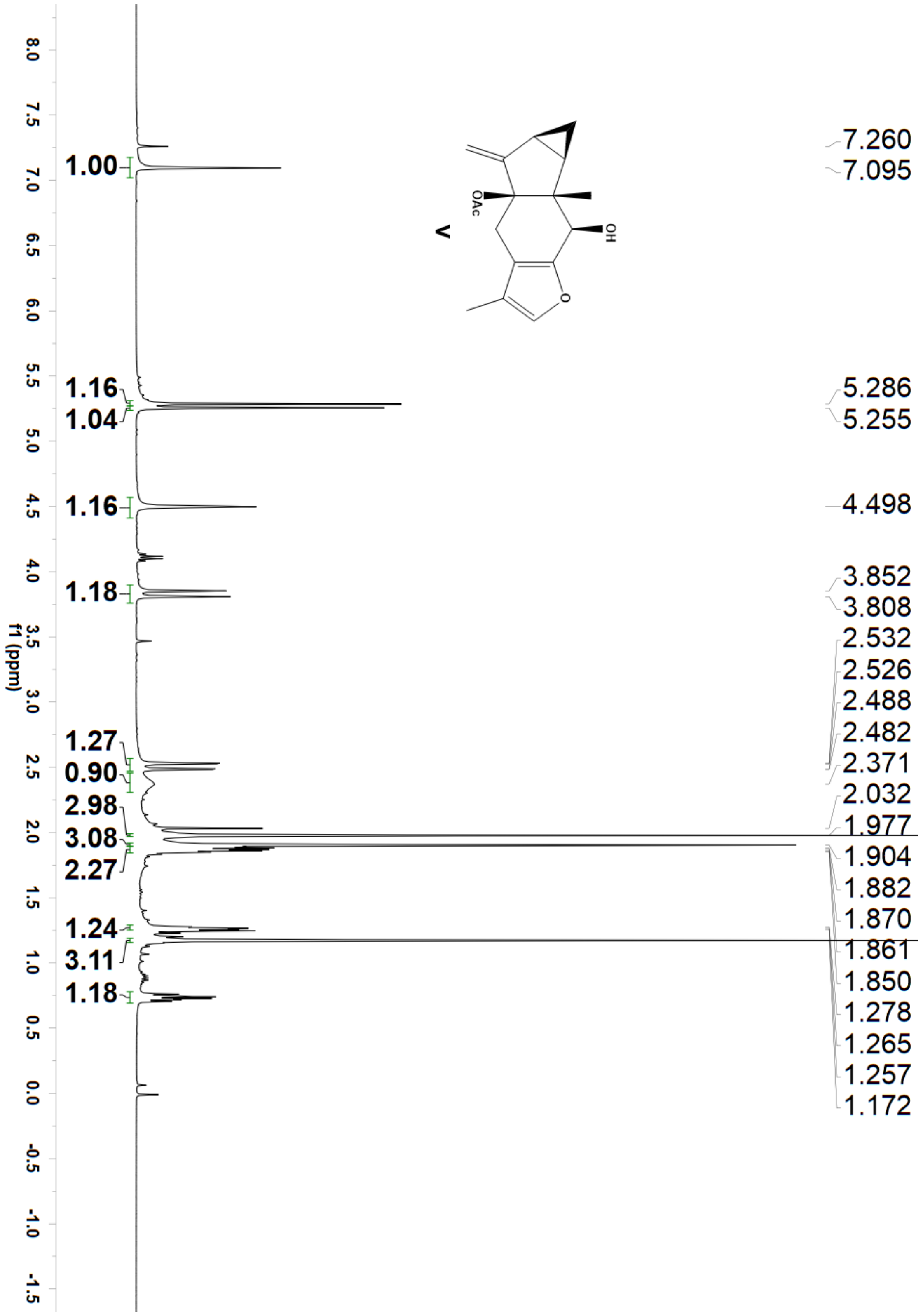

Supplementary Figure 38. ^{1}H NMR spectra of compound **v** in $CDCl_3$ (400 MHz)

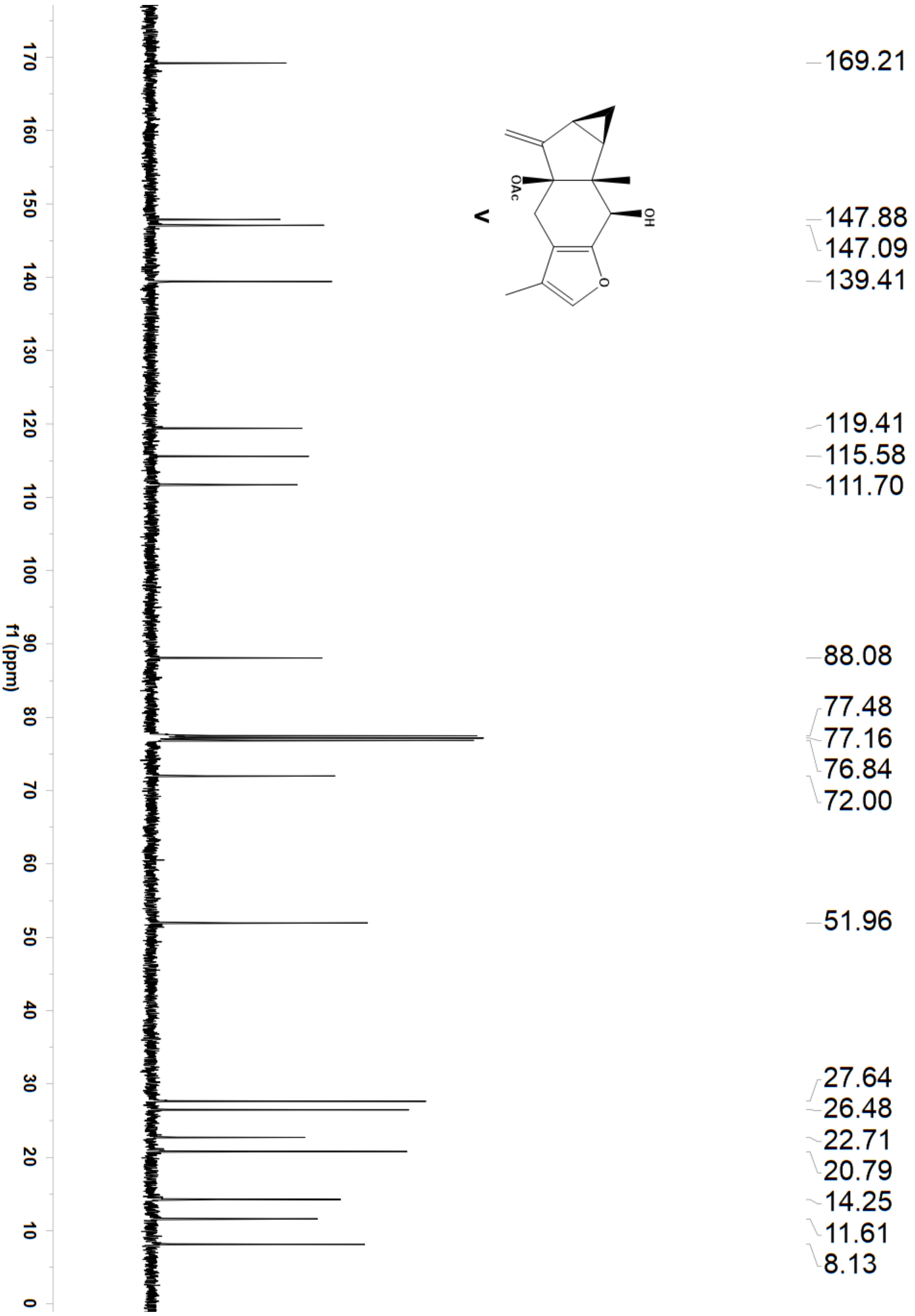

Supplementary Figure 39. ^{13}C NMR spectra of compound **v** in $CDCl_3$ (100 MHz)

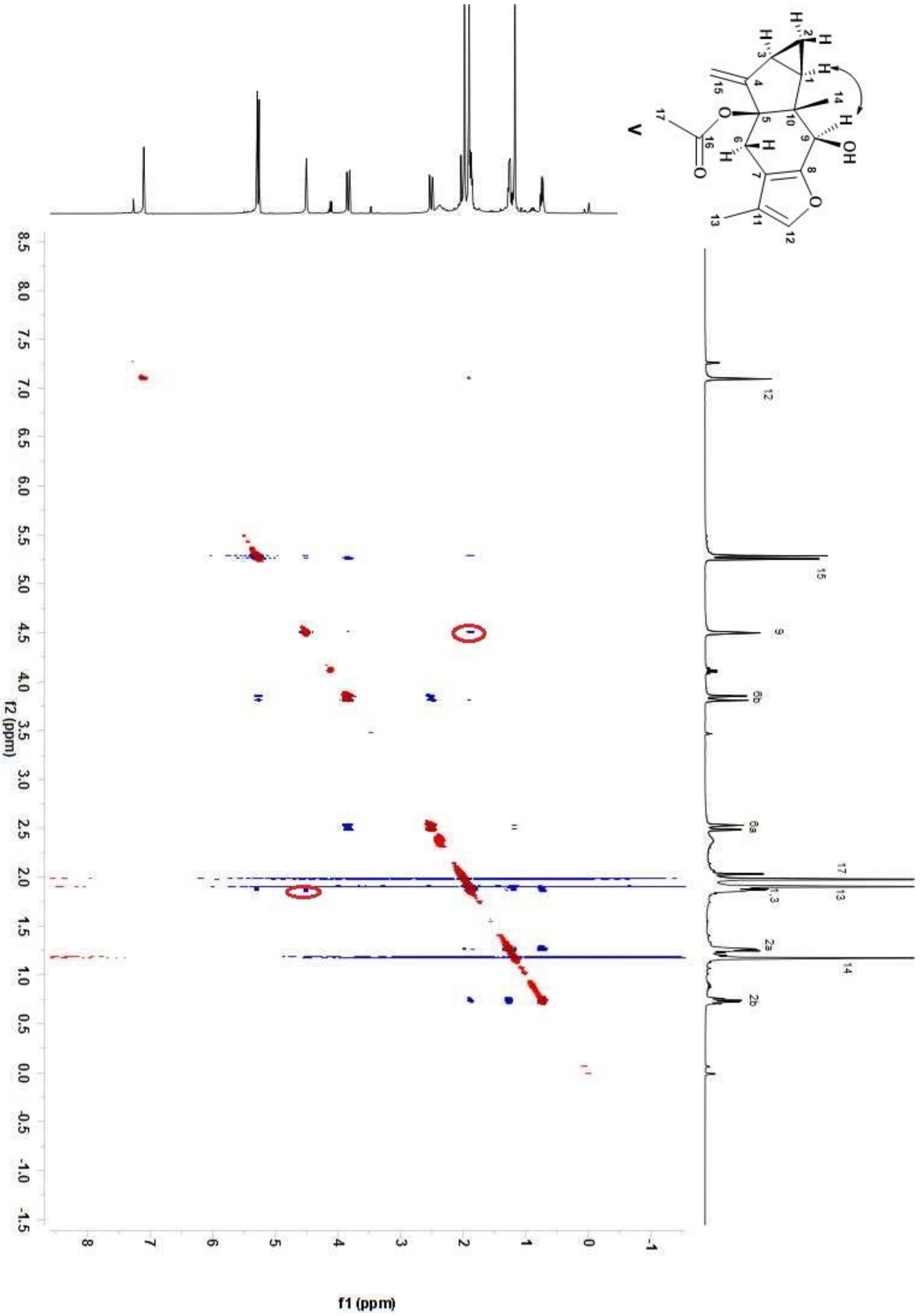

Supplementary Figure 40. NOESY NMR spectra of compound **v** in $CDCl_3$ (400 MHz)

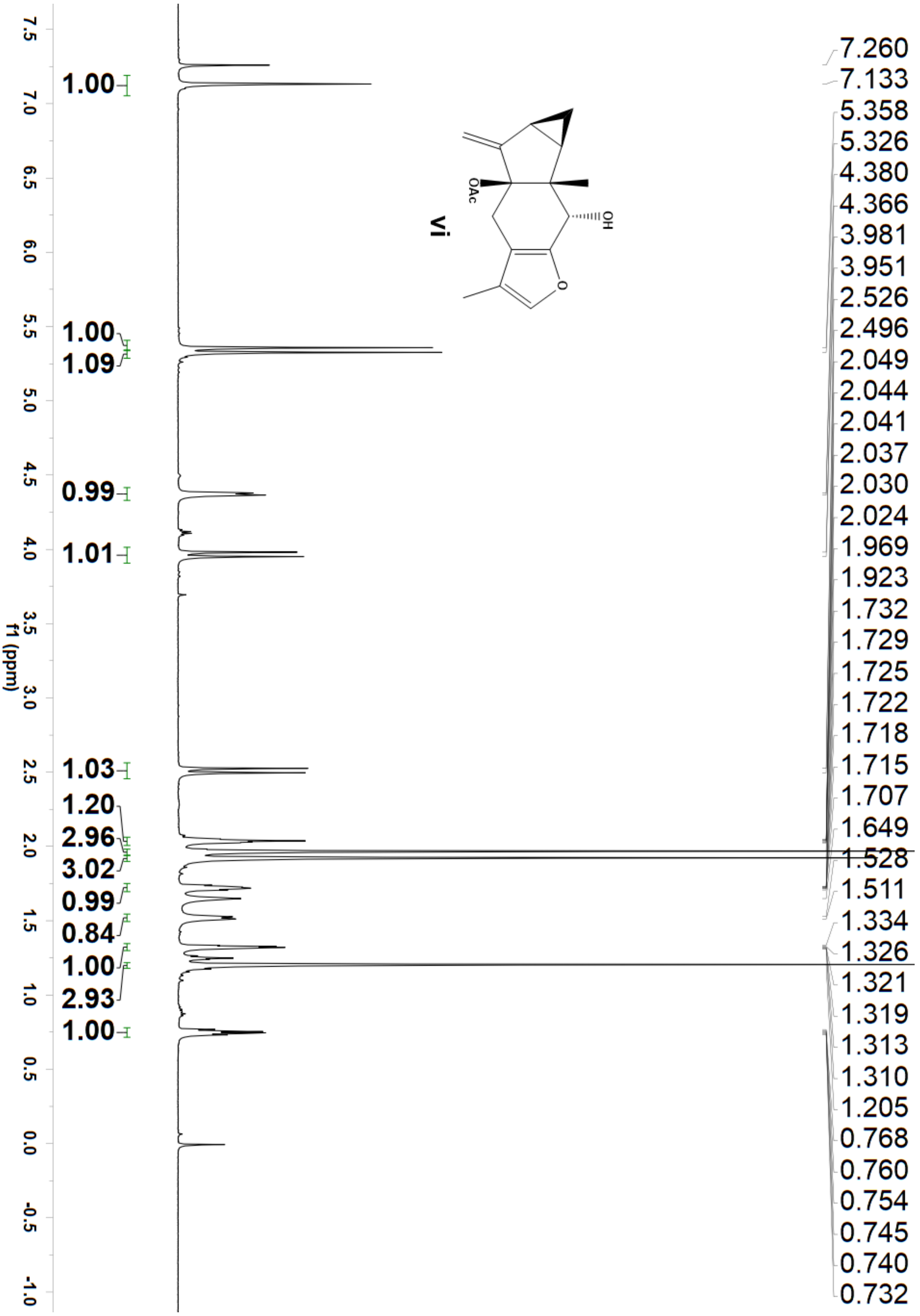

Supplementary Figure 41. ^{1}H NMR spectra of compound **vi** in $CDCl_3$ (400 MHz)

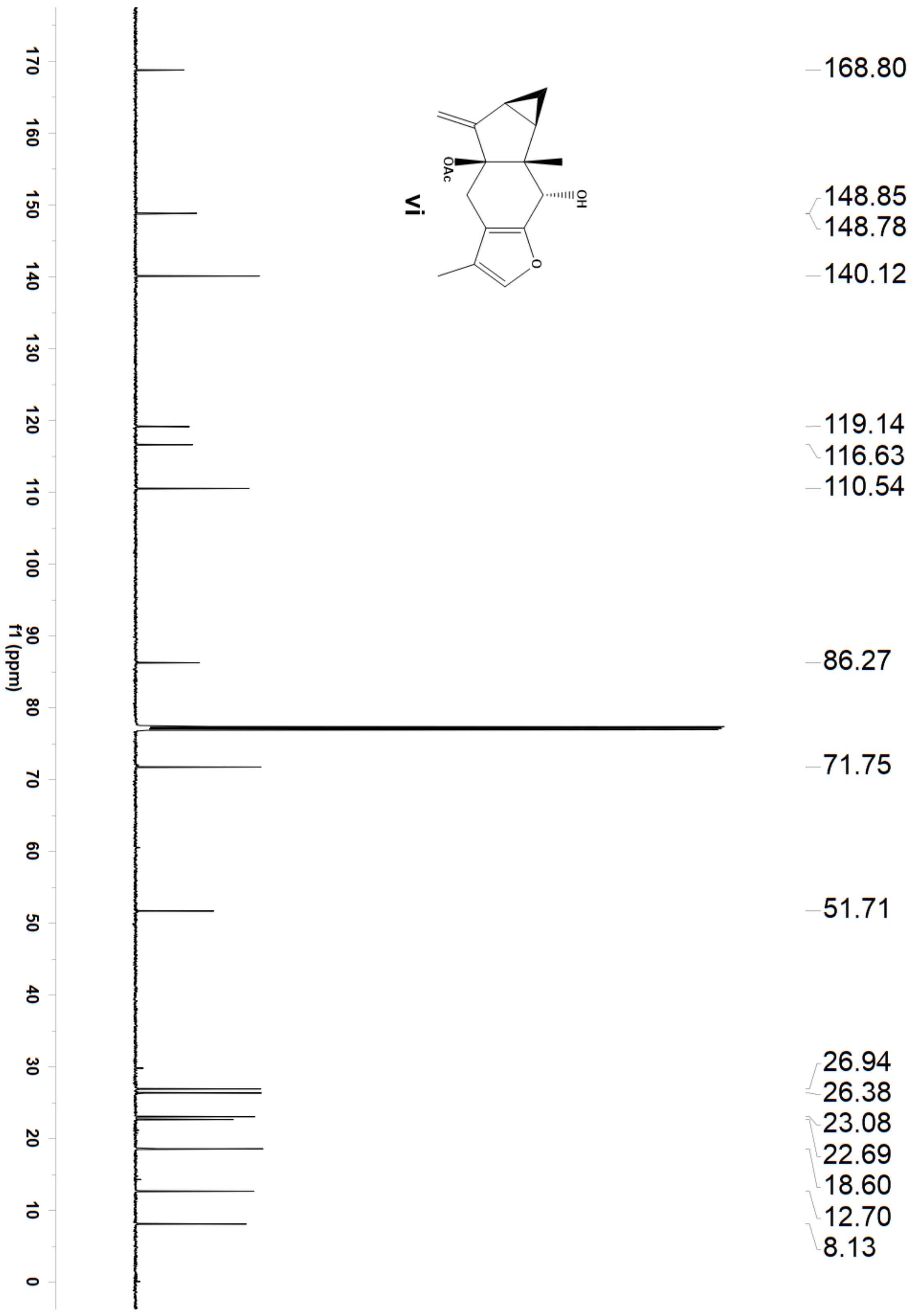

Supplementary Figure 42. ^{13}C NMR spectra of compound **vi** in $CDCl_3$ (100 MHz)

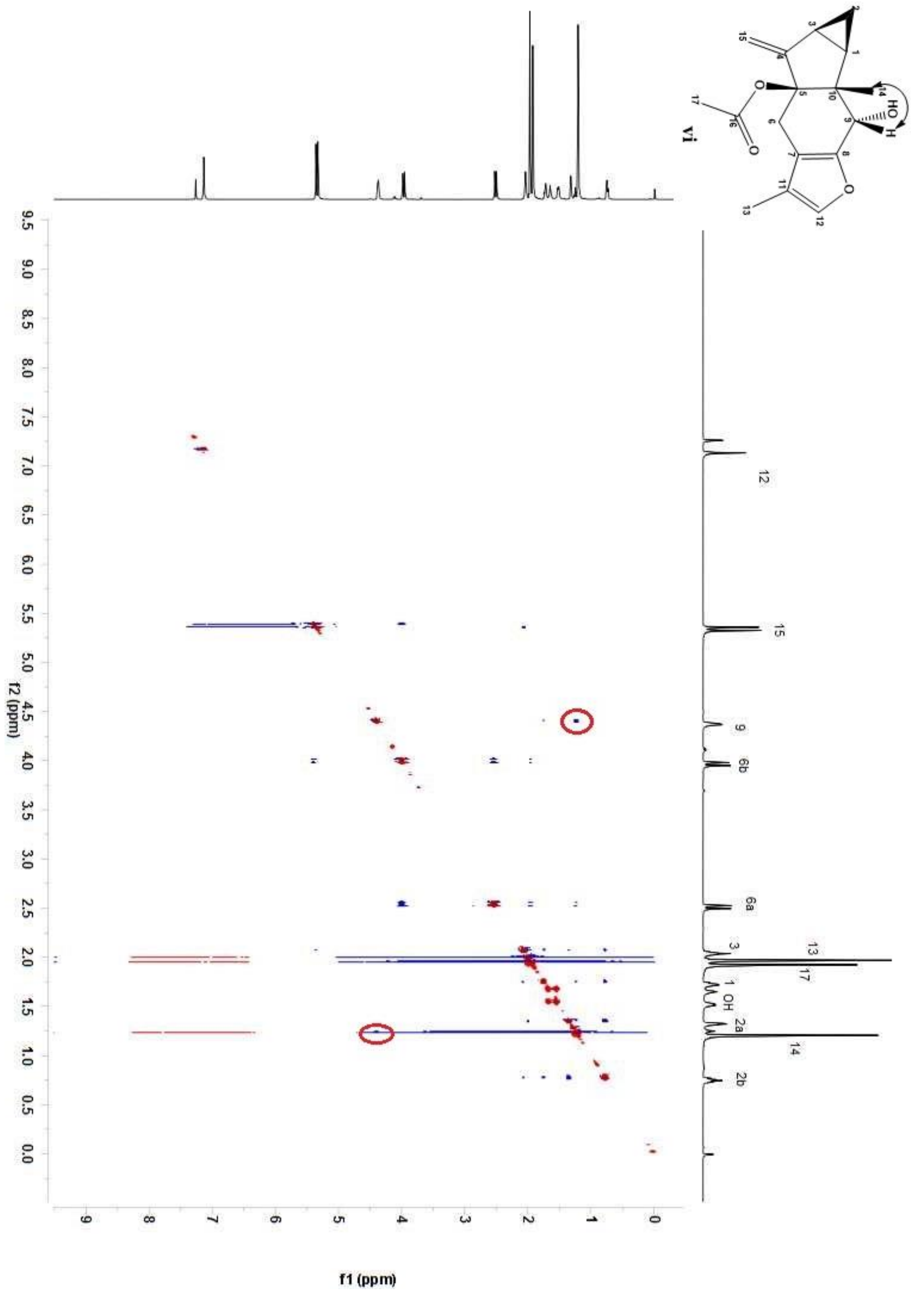

Supplementary Figure 43. NOESY NMR spectra of compound **vi** in $CDCl_3$ (400 MHz)

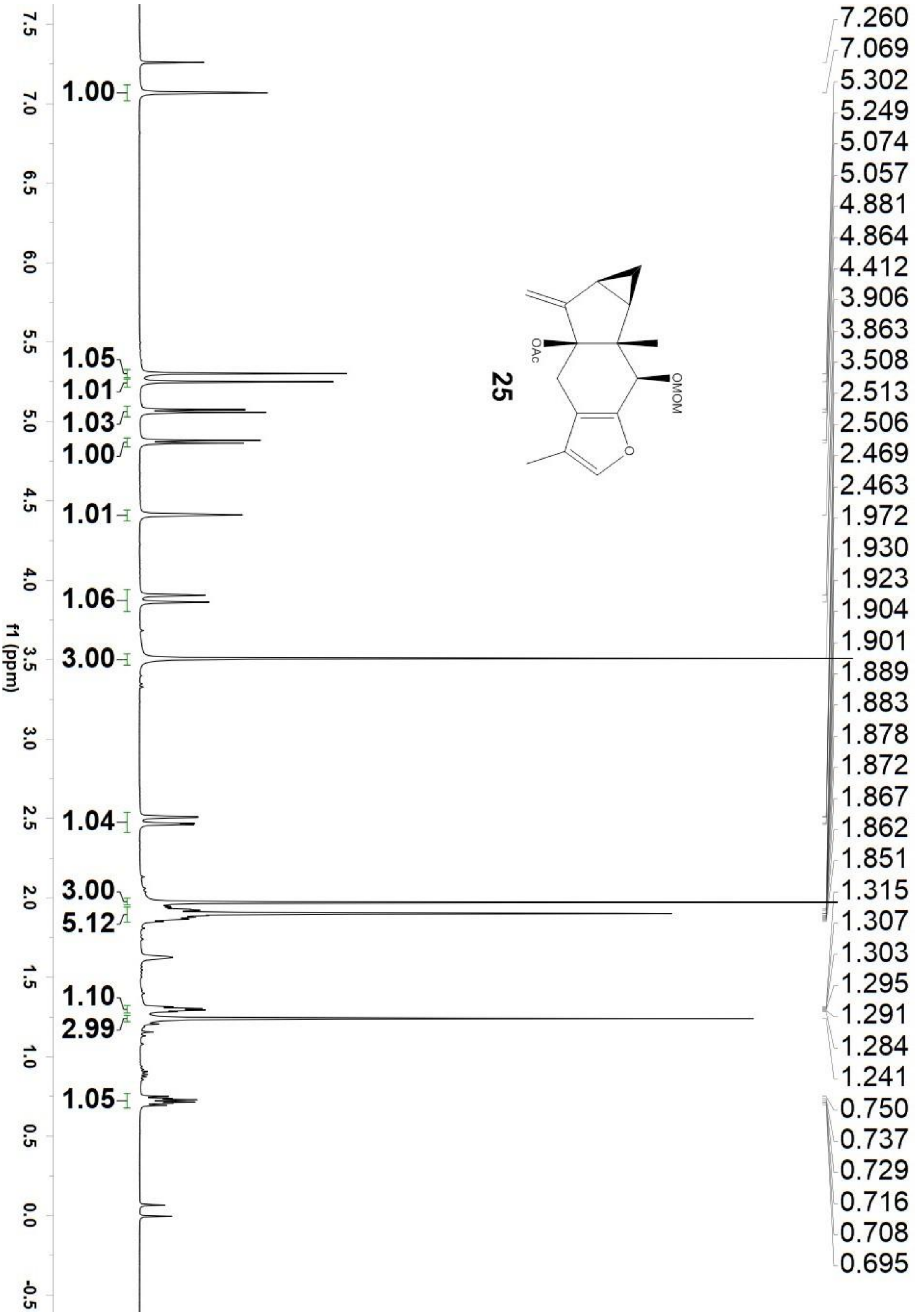

Supplementary Figure 44. ^{1}H NMR spectra of compound **25** in $CDCl_3$ (400 MHz)

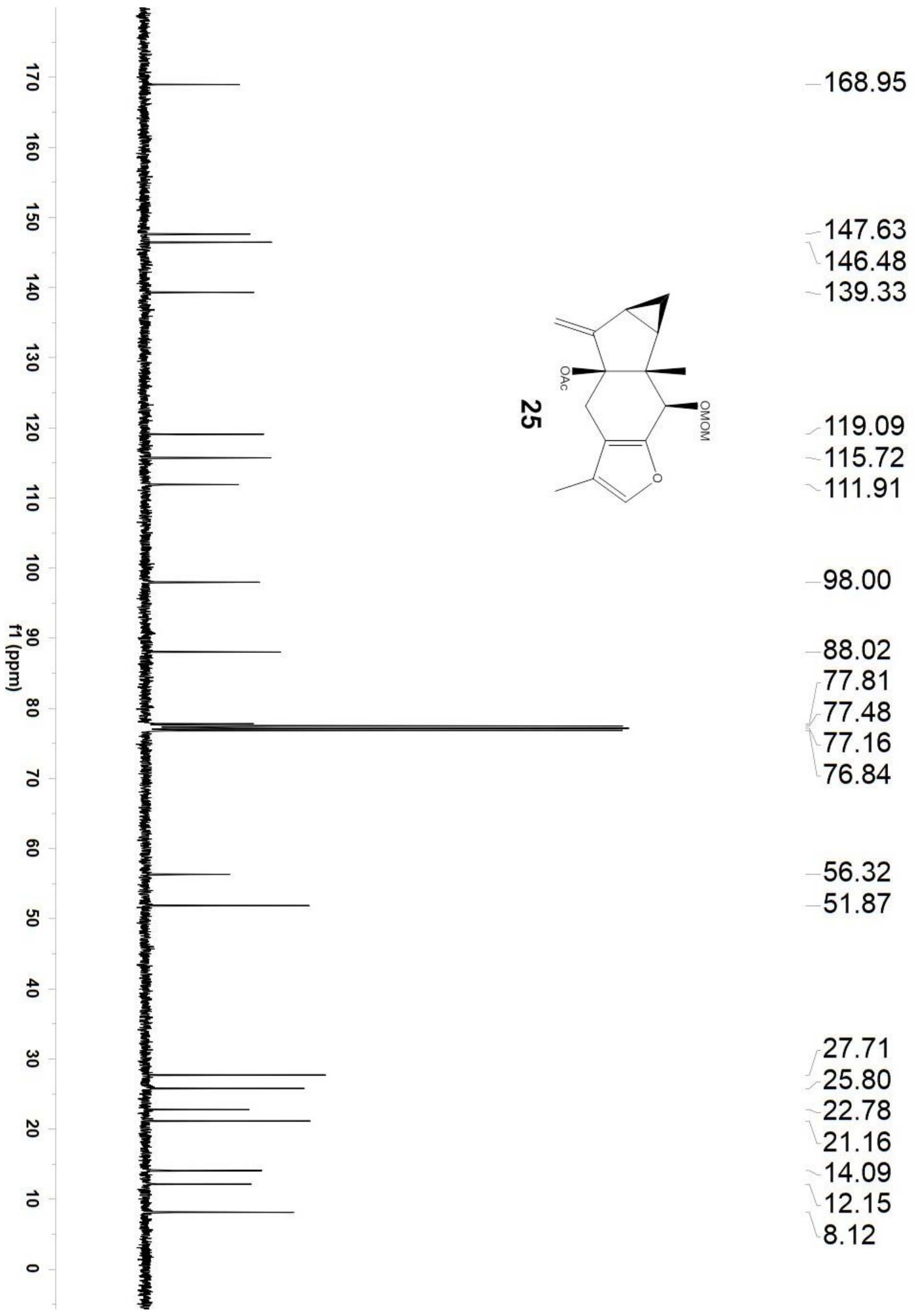

Supplementary Figure 45. ^{13}C NMR spectra of compound **25** in $CDCl_3$ (100 MHz)

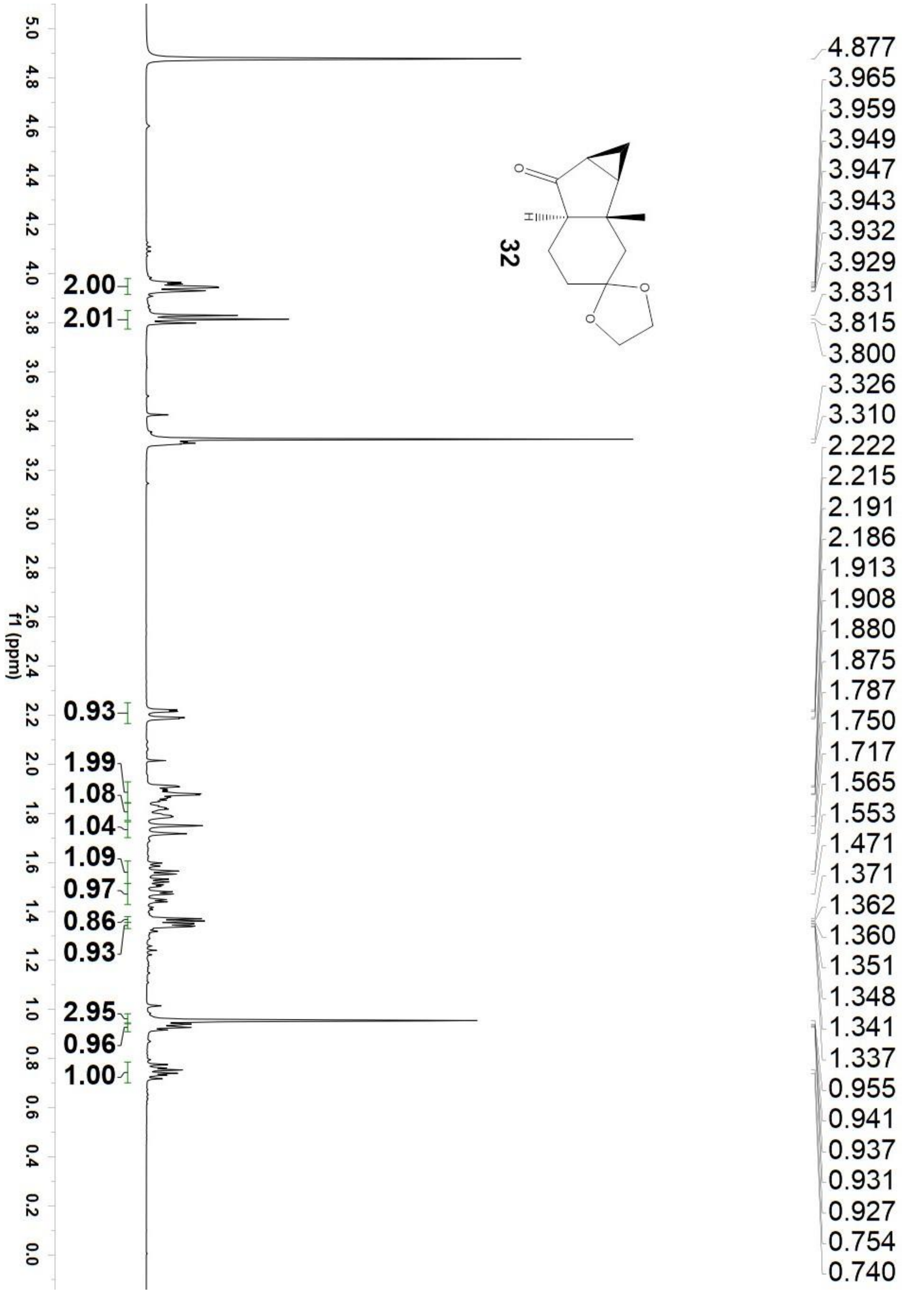

Supplementary Figure 46. ^{1}H NMR spectra of compound **32** in CD_3OD (400 MHz)

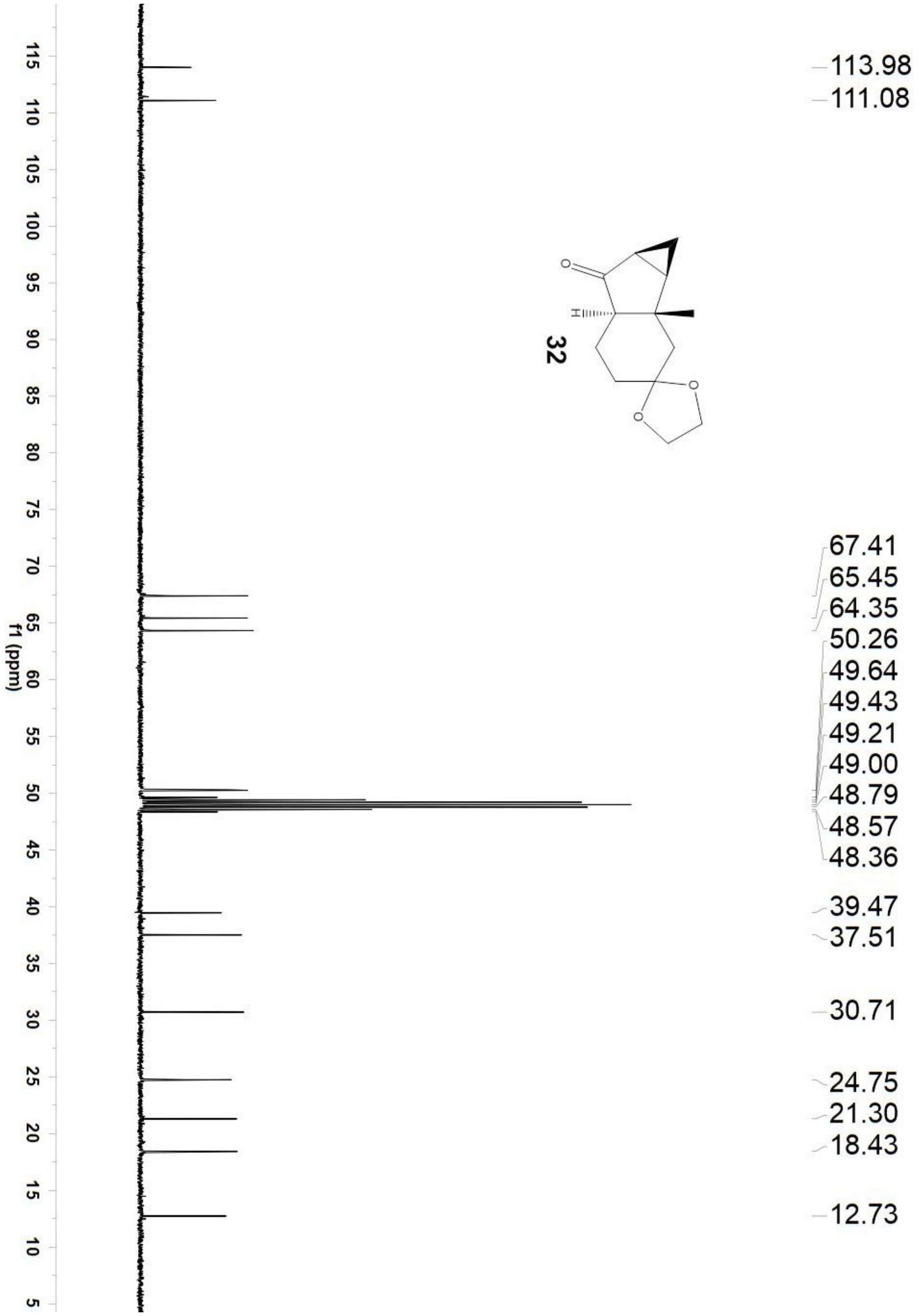

Supplementary Figure 47. ^{13}C NMR spectra of compound **32** in CD_3OD (100 MHz)

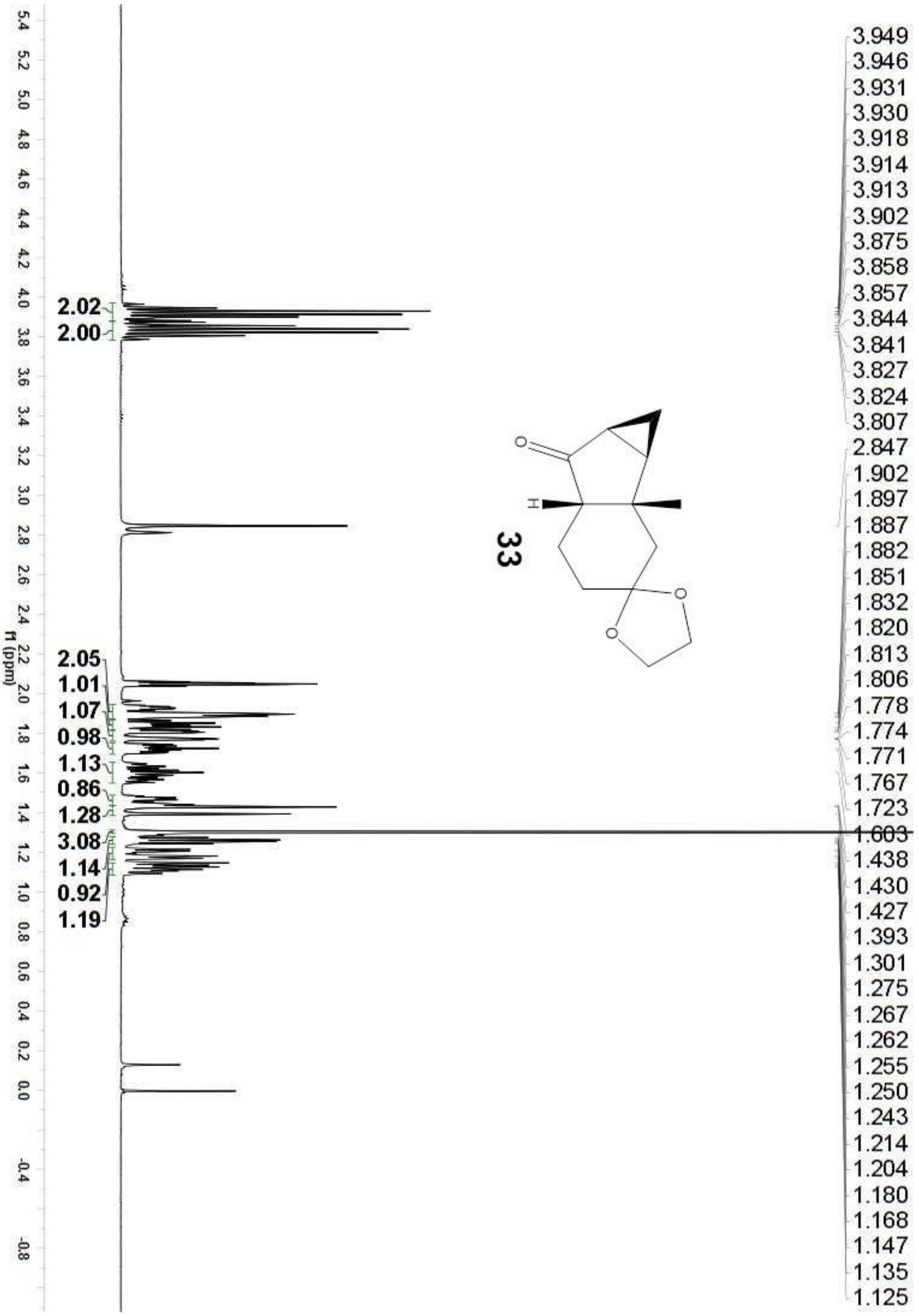

Supplementary Figure 48. ^{1}H NMR spectra of compound **33** in CD_3COCD_3 (400 MHz)

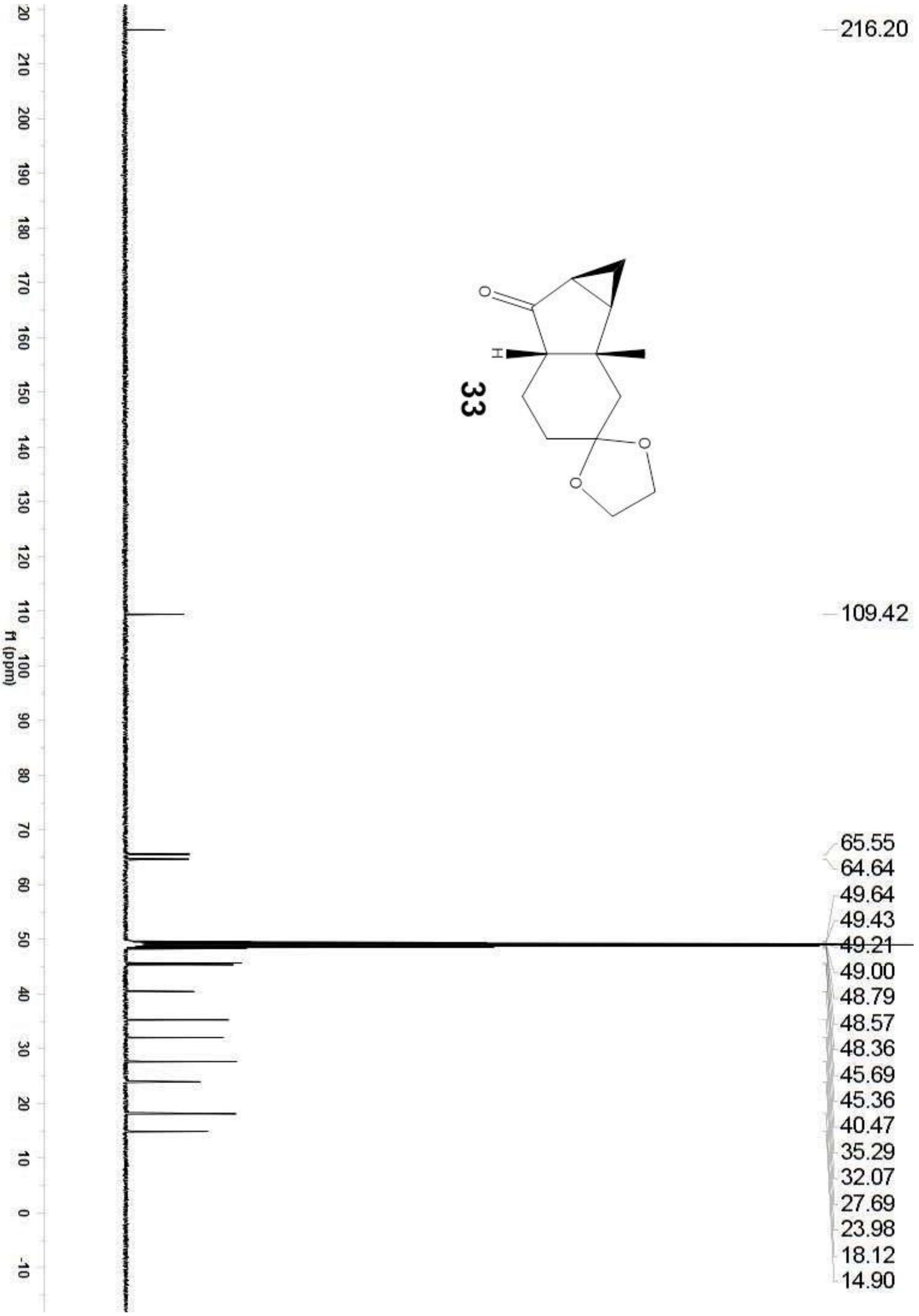

Supplementary Figure 49. ^{13}C NMR spectra of compound **33** in CD_3COCD_3 (100 MHz)

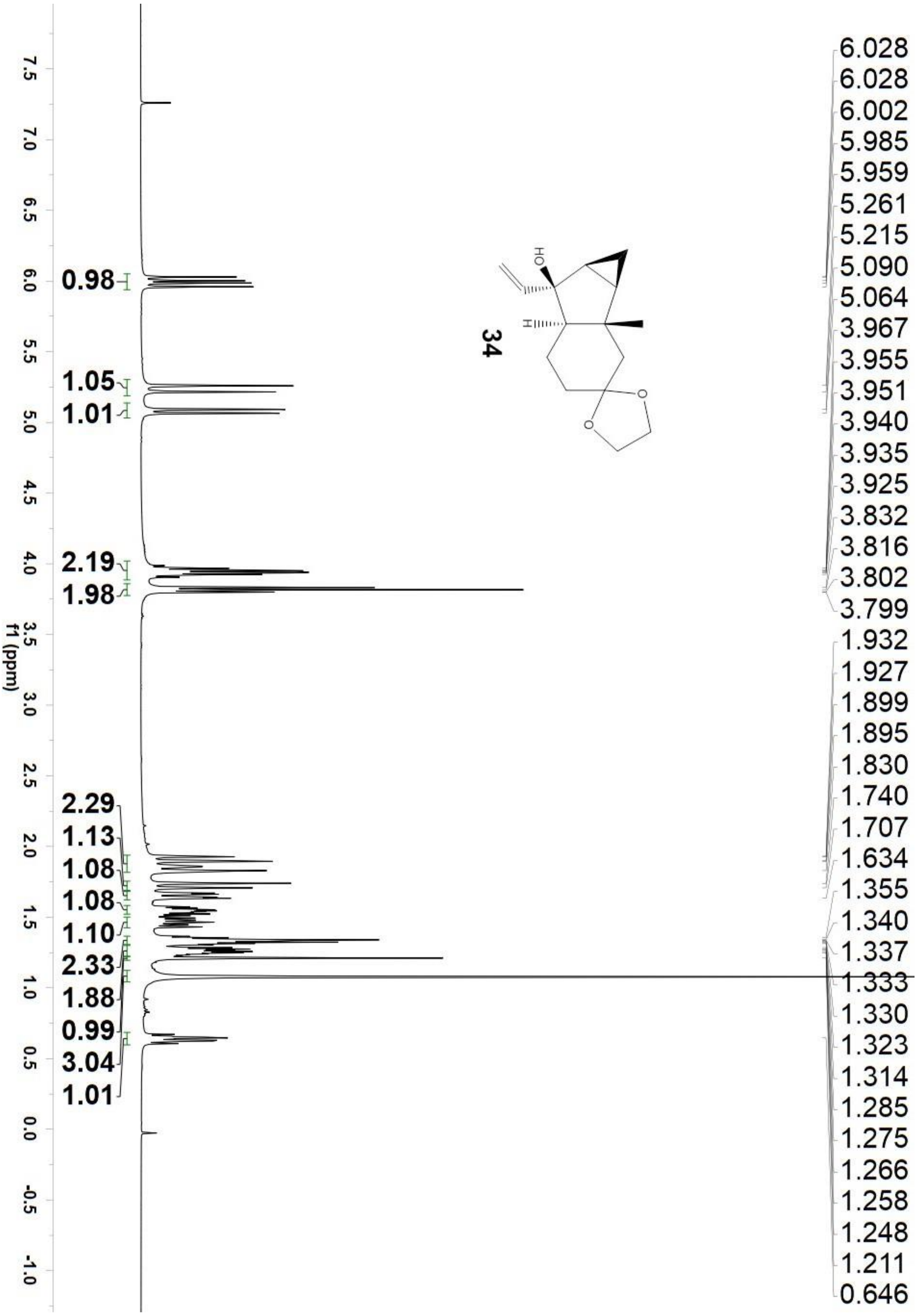

Supplementary Figure 50. ^{1}H NMR spectra of compound **34** in $CDCl_3$ (400 MHz)

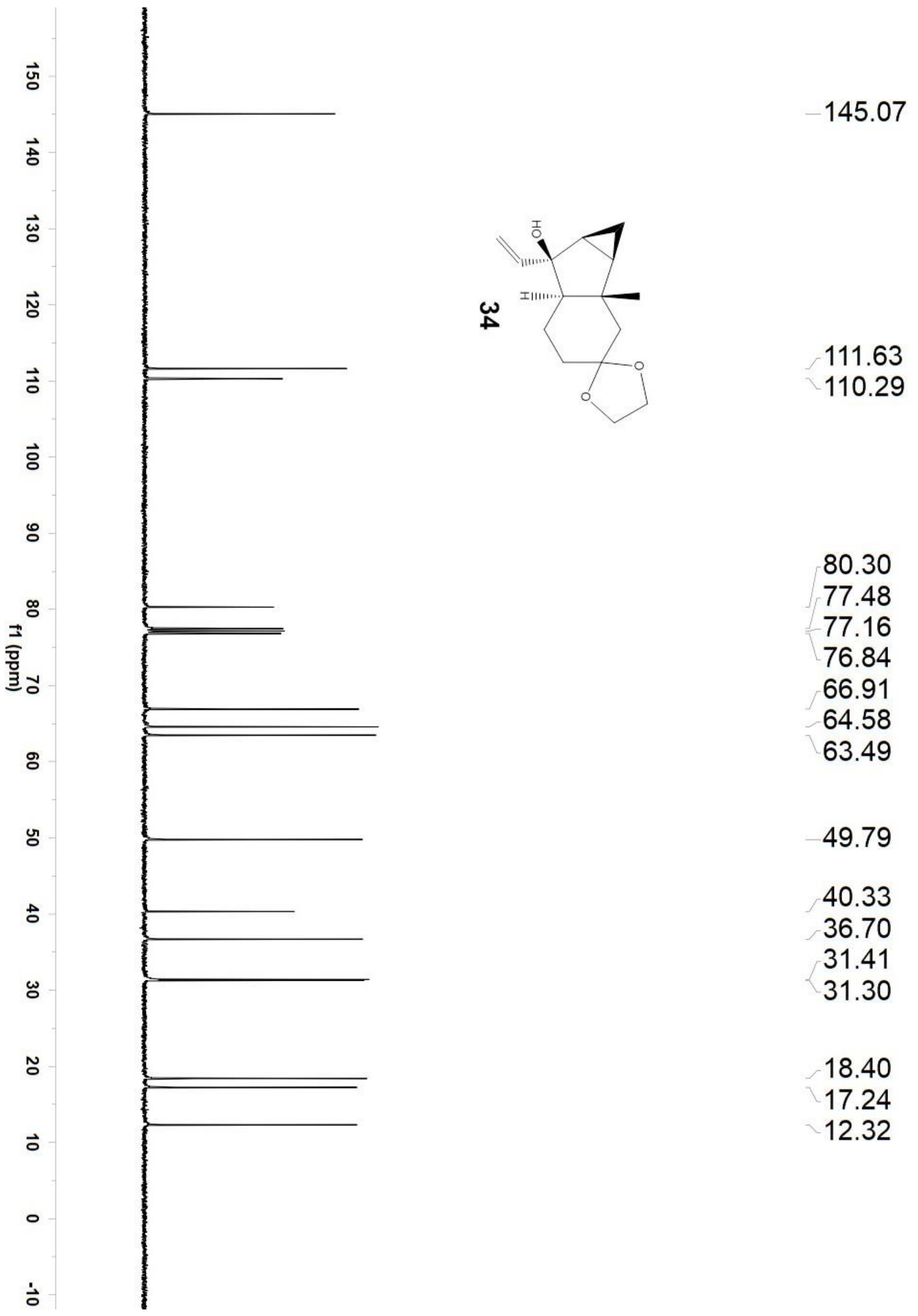

Supplementary Figure 51. ^{13}C NMR spectra of compound **34** in $CDCl_3$ (100 MHz)

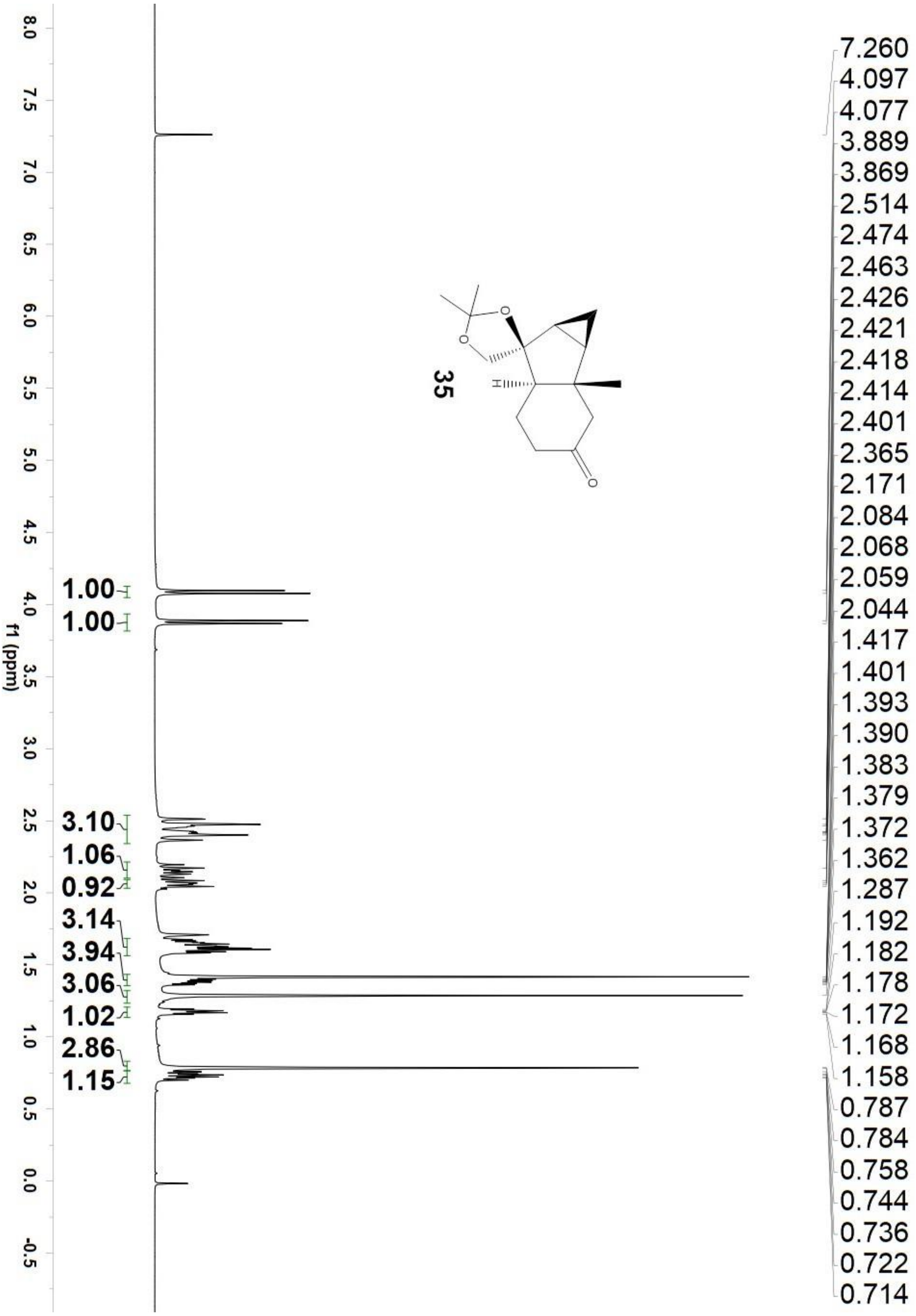

Supplementary Figure 52. ^{1}H NMR spectra of compound **35** in $CDCl_3$ (400 MHz)

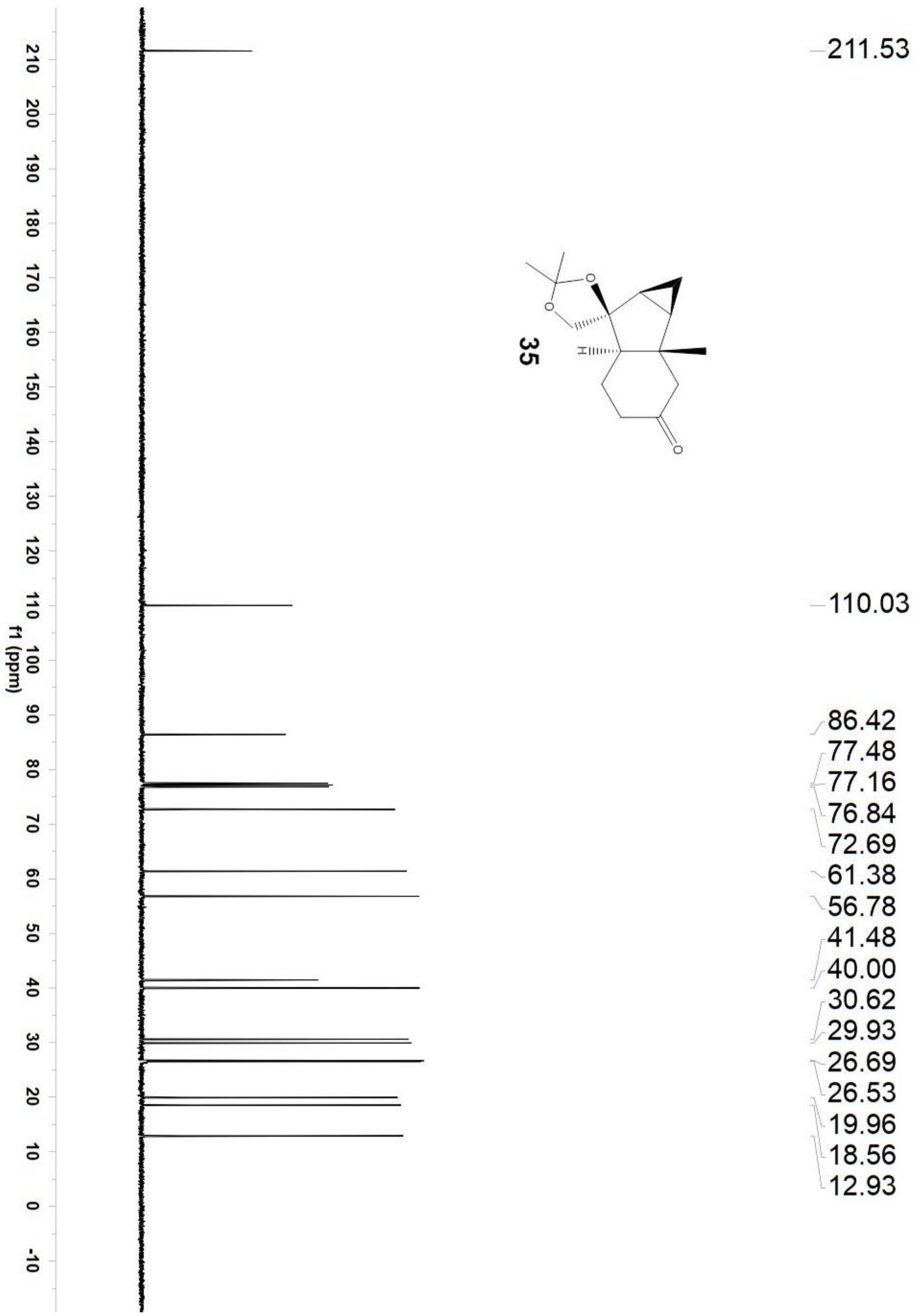

Supplementary Figure 53. ^{13}C NMR spectra of compound **35** in $CDCl_3$ (100 MHz)

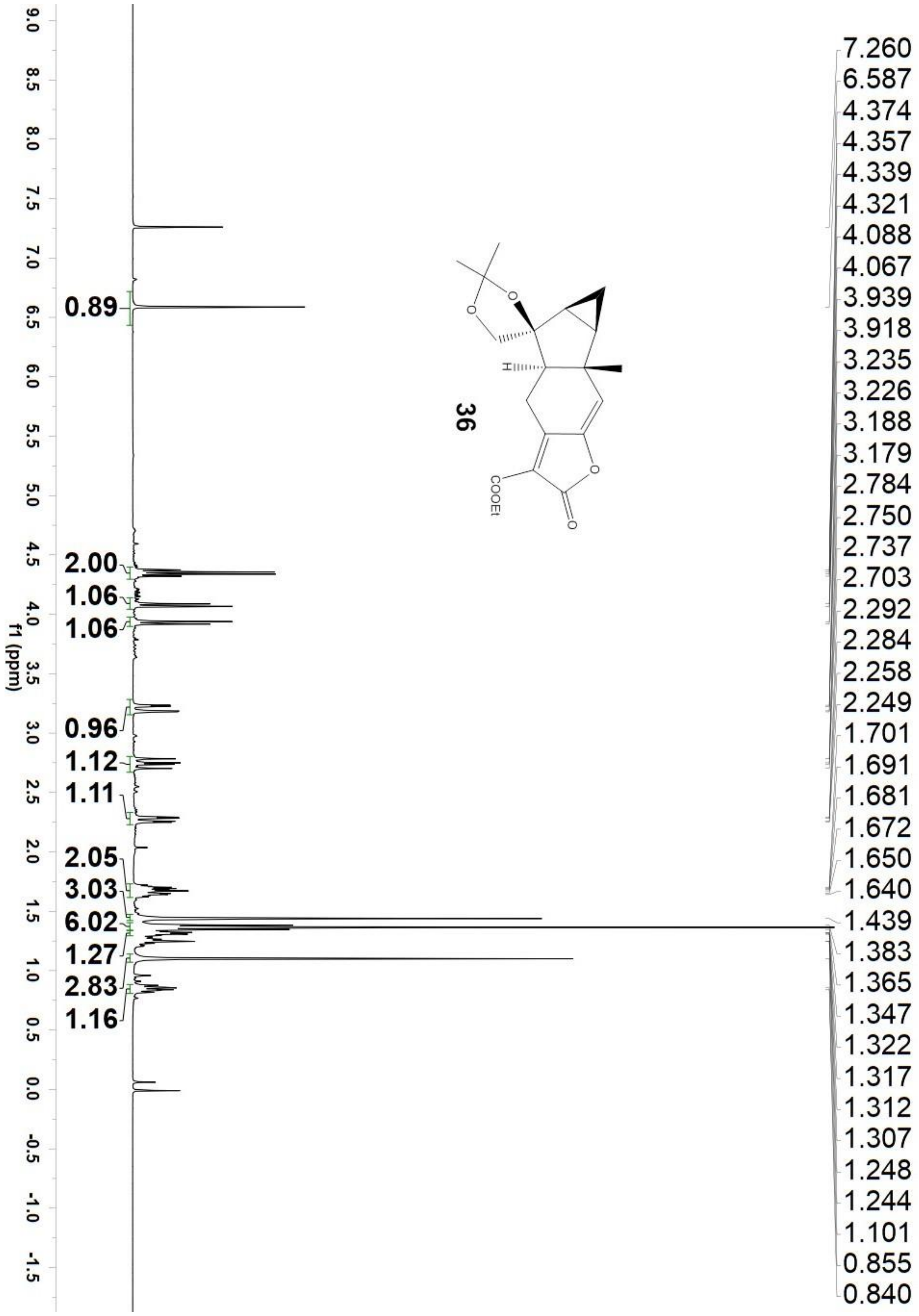

Supplementary Figure 54. ^{1}H NMR spectra of compound **36** in $CDCl_3$ (400 MHz)

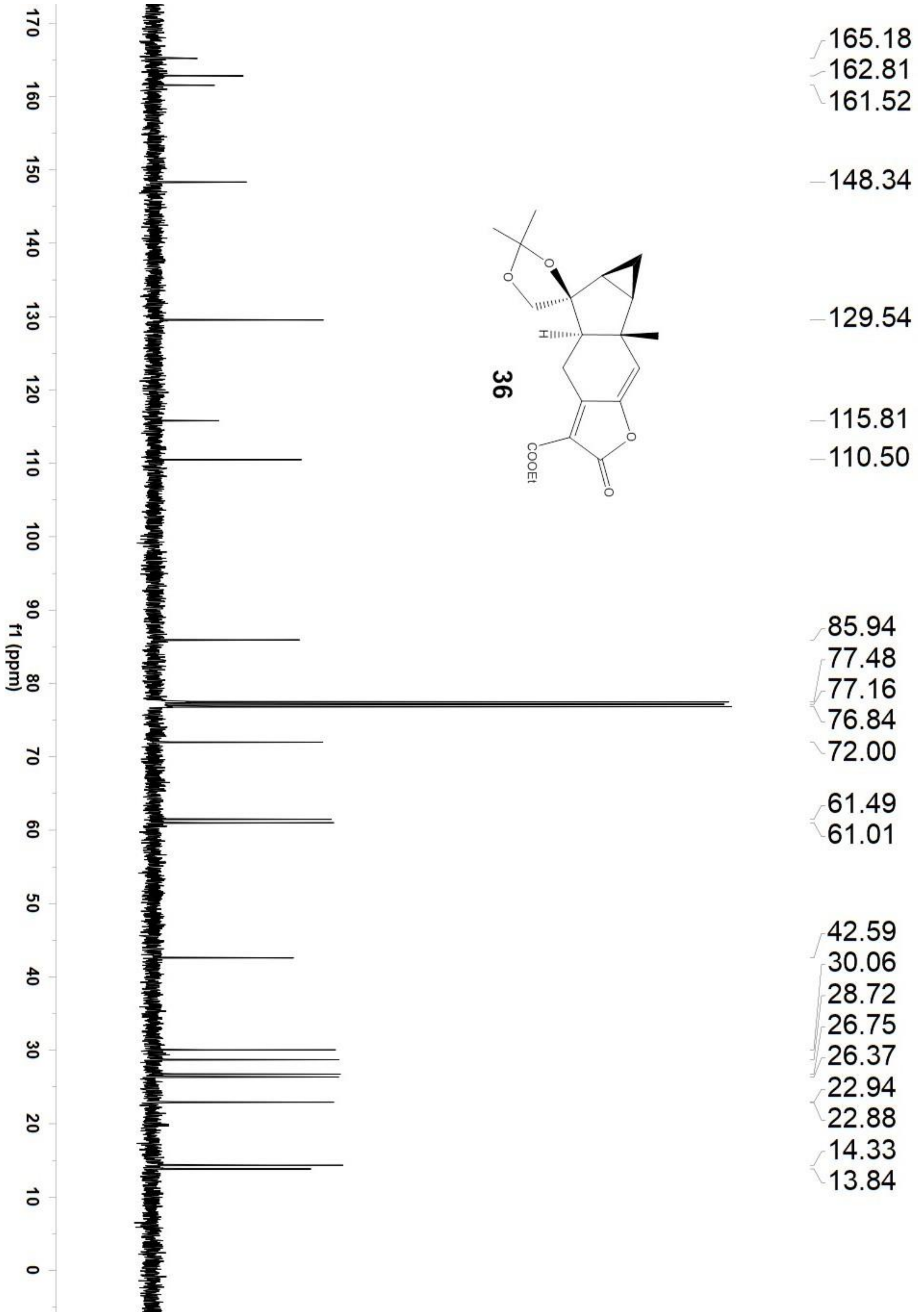

Supplementary Figure 55. ^{13}C NMR spectra of compound **36** in $CDCl_3$ (100 MHz)

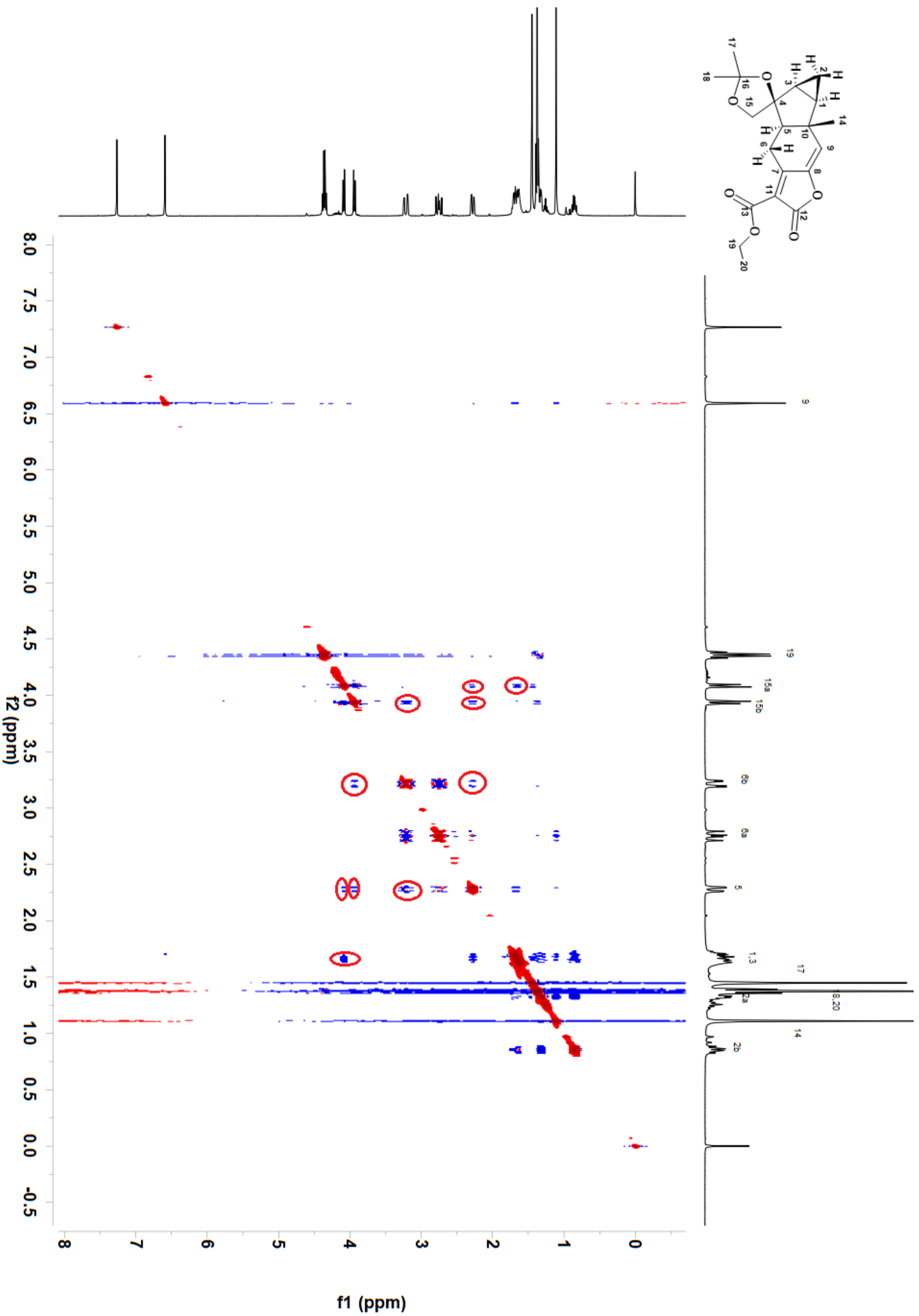

Supplementary Figure 56. NOESY NMR spectra of compound **36** in $CDCl_3$ (400 MHz)

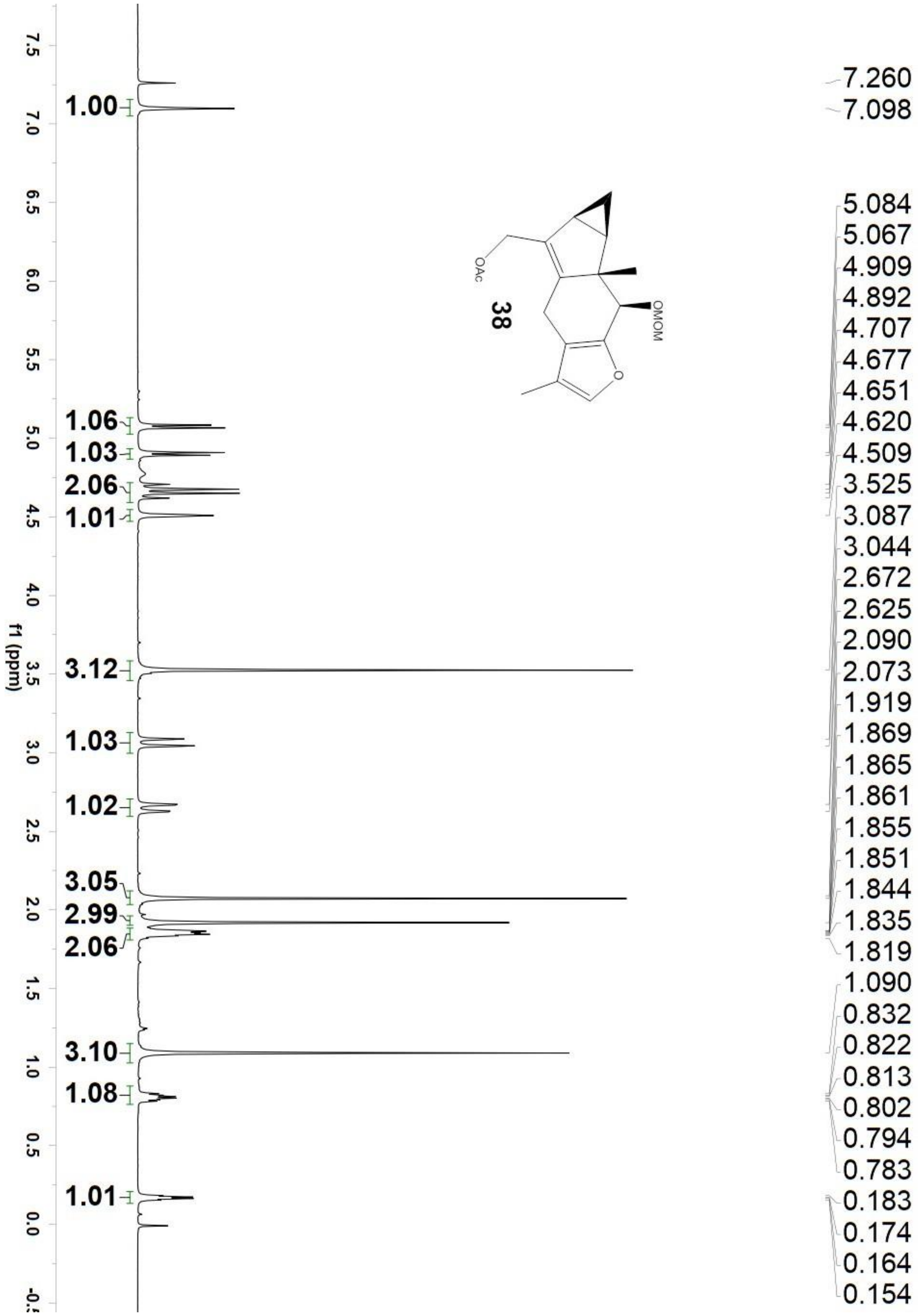

Supplementary Figure 57. ^{1}H NMR spectra of compound **38** in $CDCl_3$ (400 MHz)

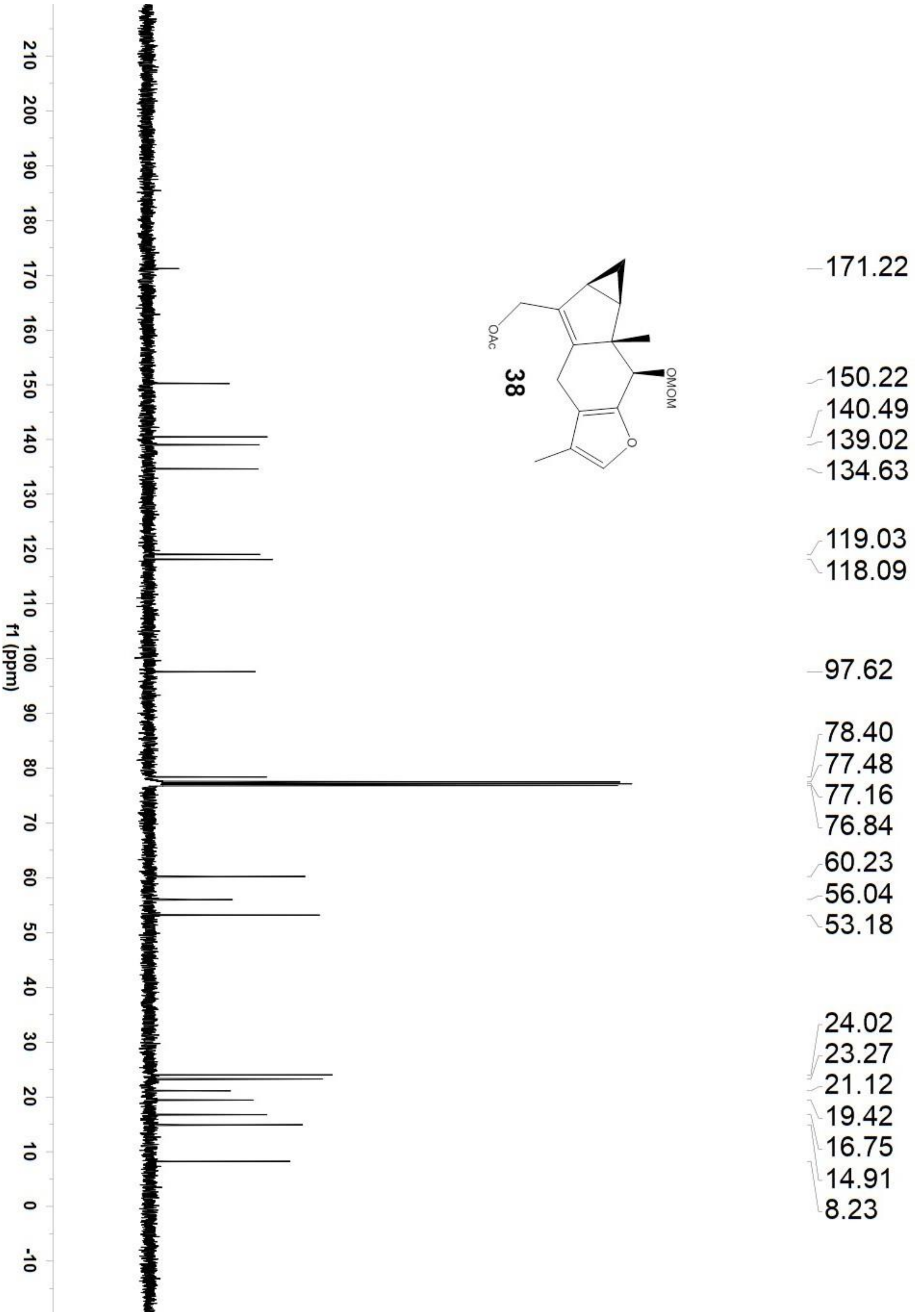

Supplementary Figure 58. ^{13}C NMR spectra of compound **38** in $CDCl_3$ (100 MHz)

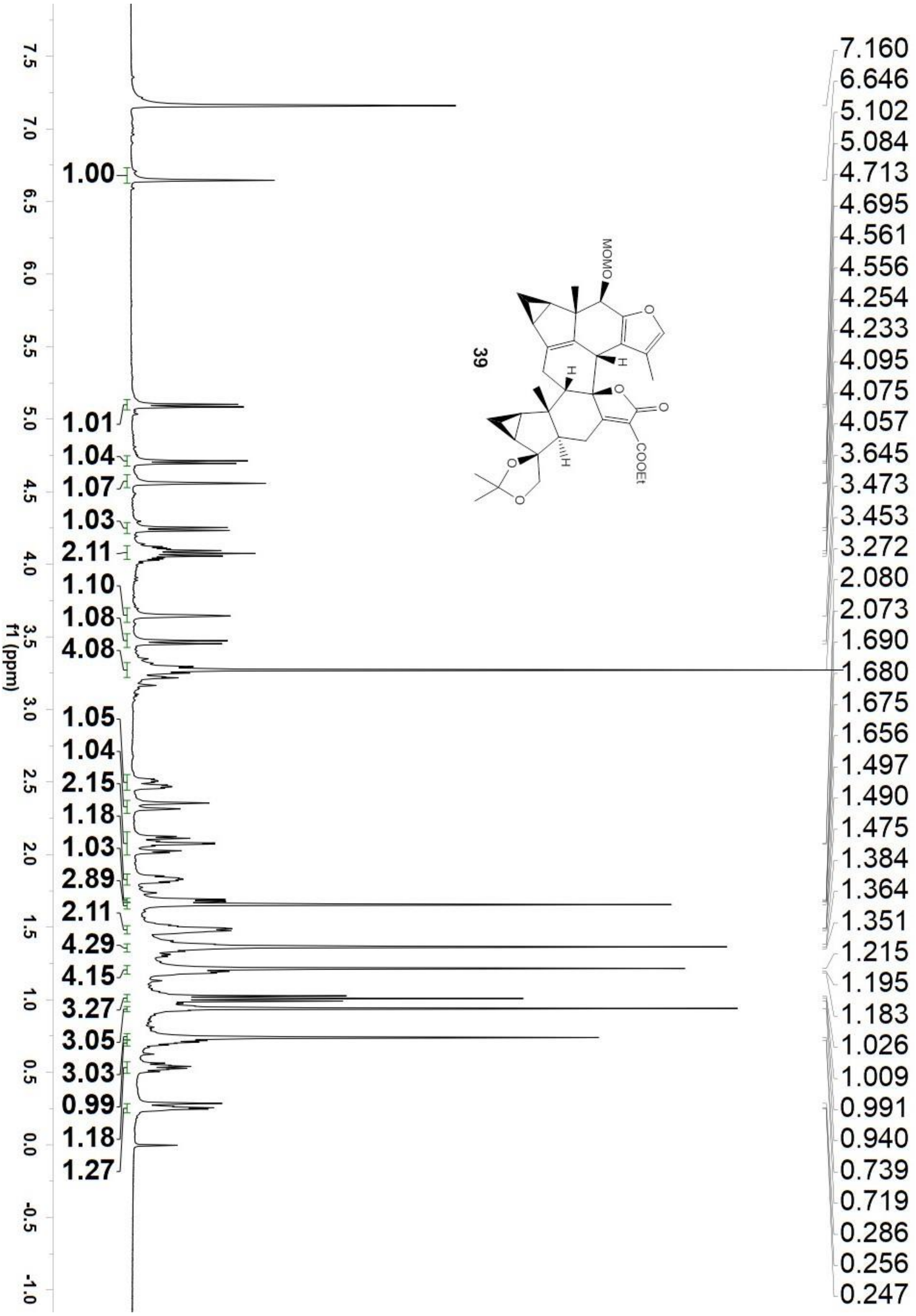

Supplementary Figure 59. ^{1}H NMR spectra of compound **39** in C_6D_6 (400 MHz)

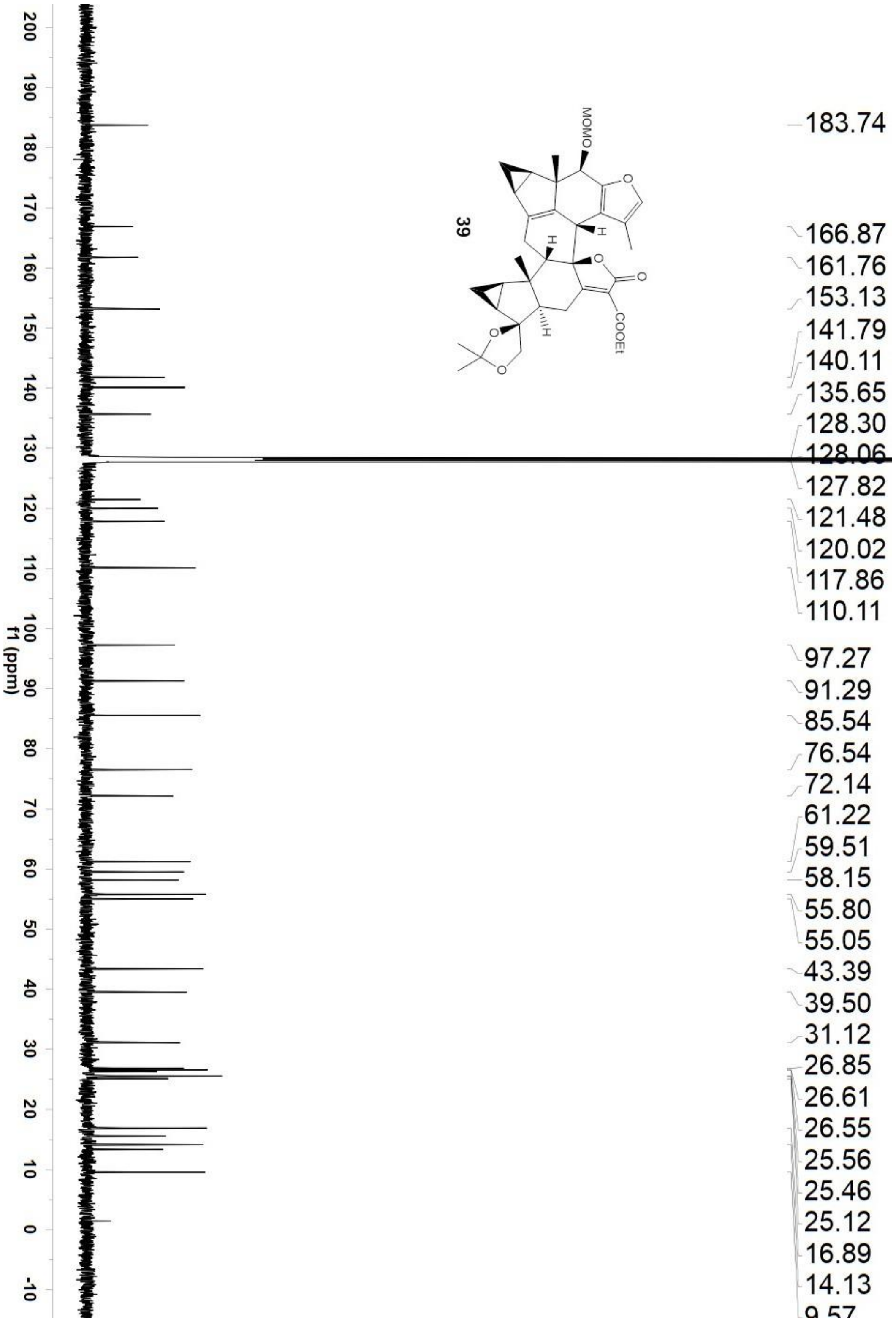

Supplementary Figure 60. ^{13}C NMR spectra of compound **39** in C_6D_6 (100 MHz)

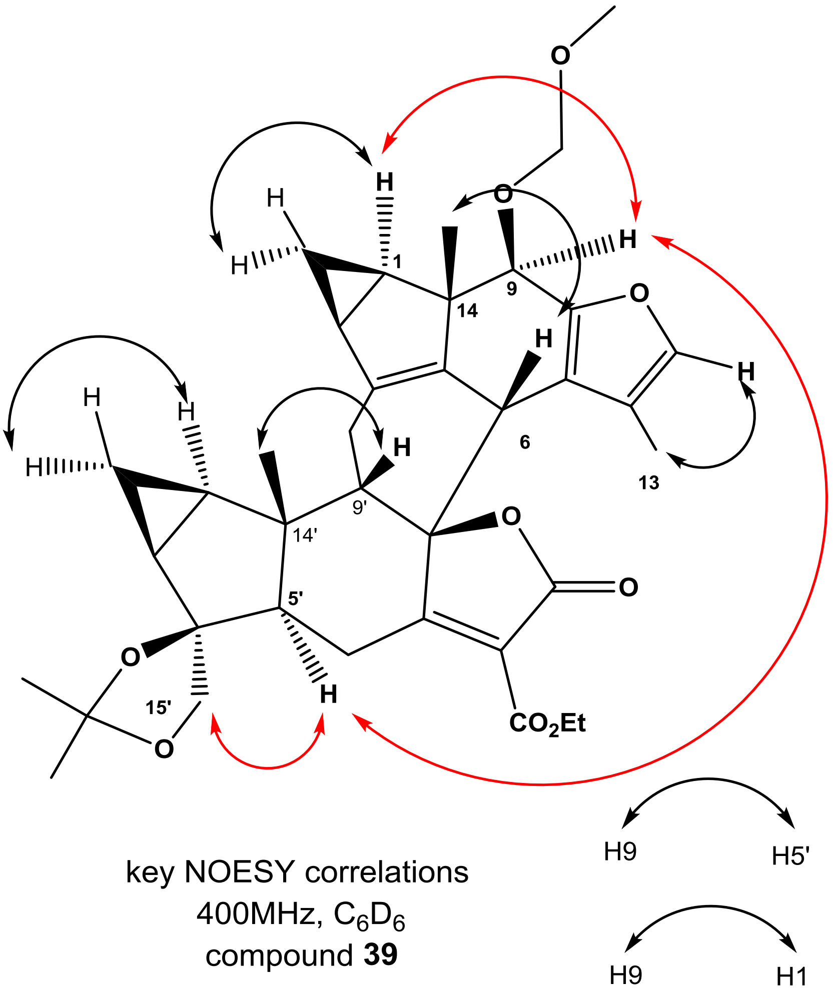

key NOESY correlations
400MHz, C_6D_6
compound **39**

Supplementary Figure 61. Key NOESY correlations of compound **39**

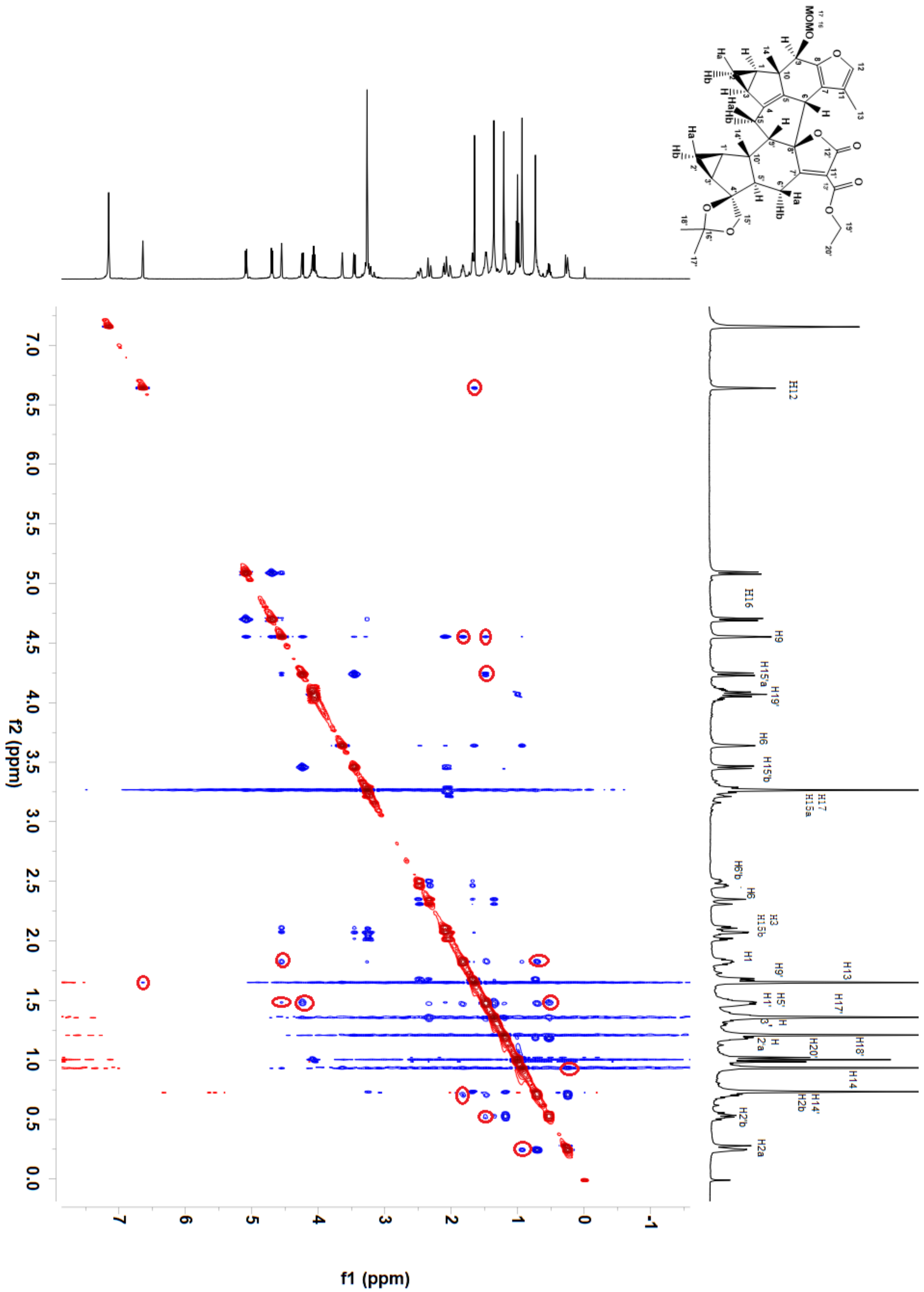

Supplementary Figure 62. NOESY NMR spectra of compound **39** in C_6D_6 (400 MHz)

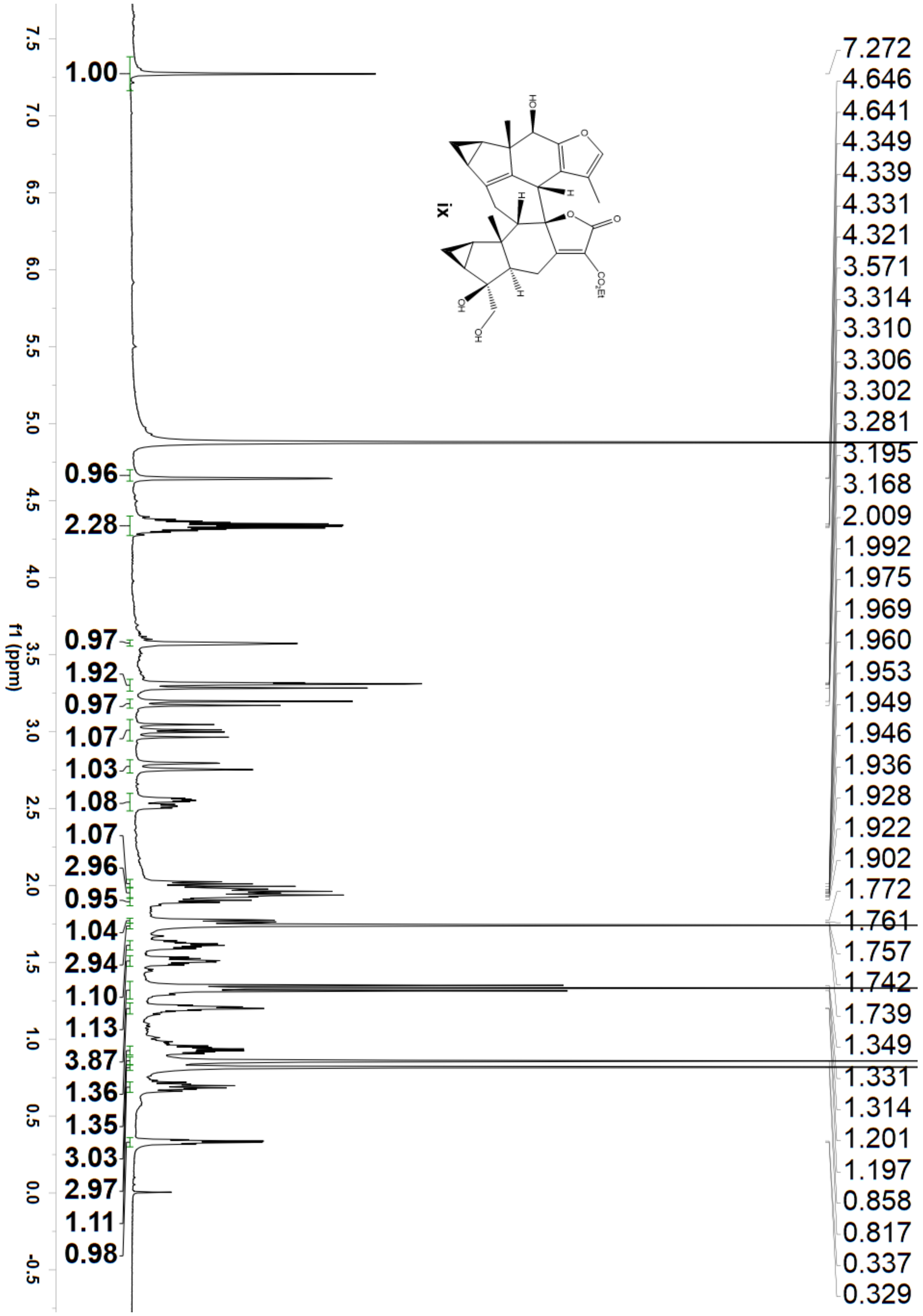

Supplementary Figure 63. ^{1}H NMR spectra of compound **ix** in CD_3OD (400 MHz)

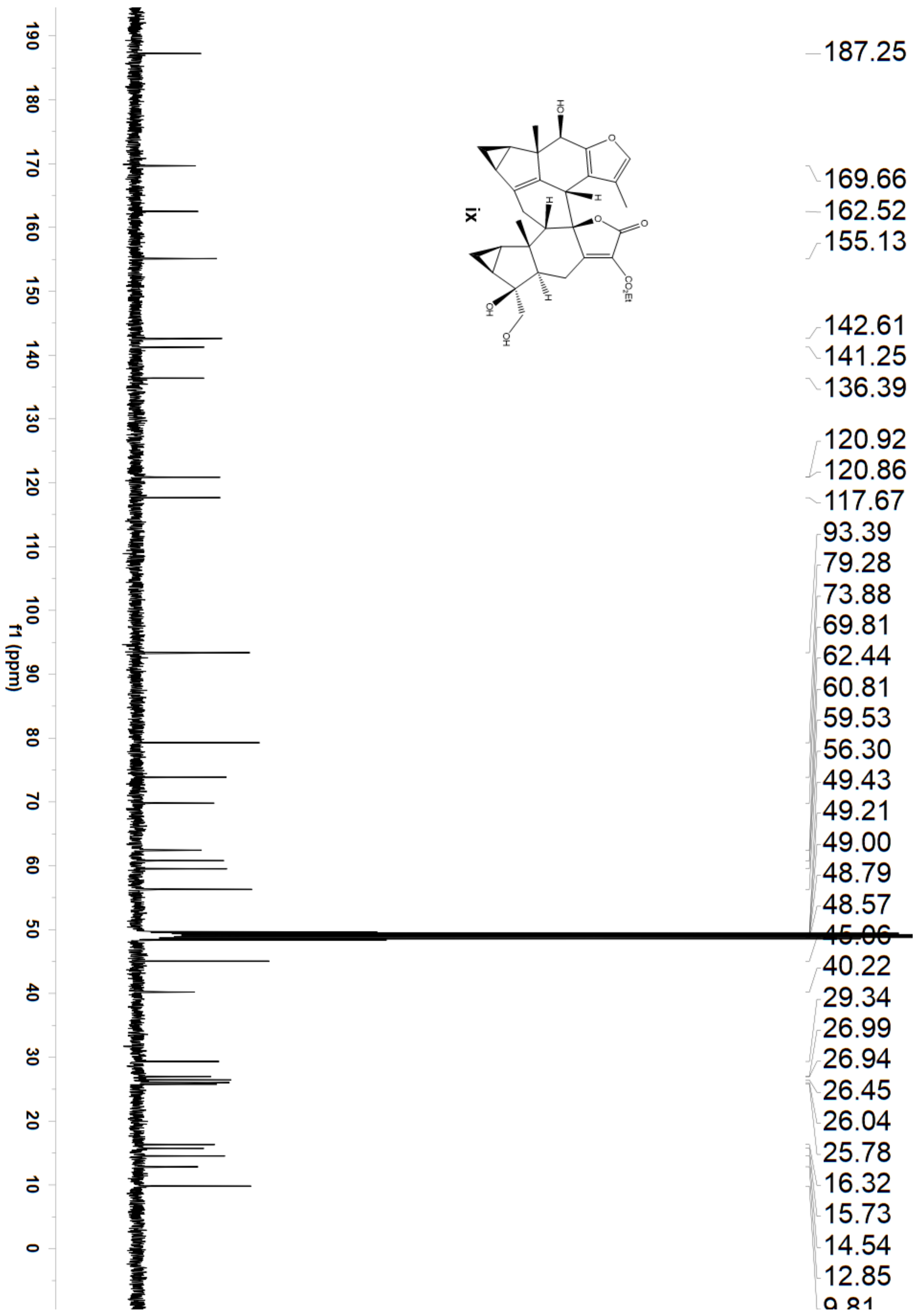

Supplementary Figure 64. ^{13}C NMR spectra of compound **ix** in CD_3OD (100 MHz)

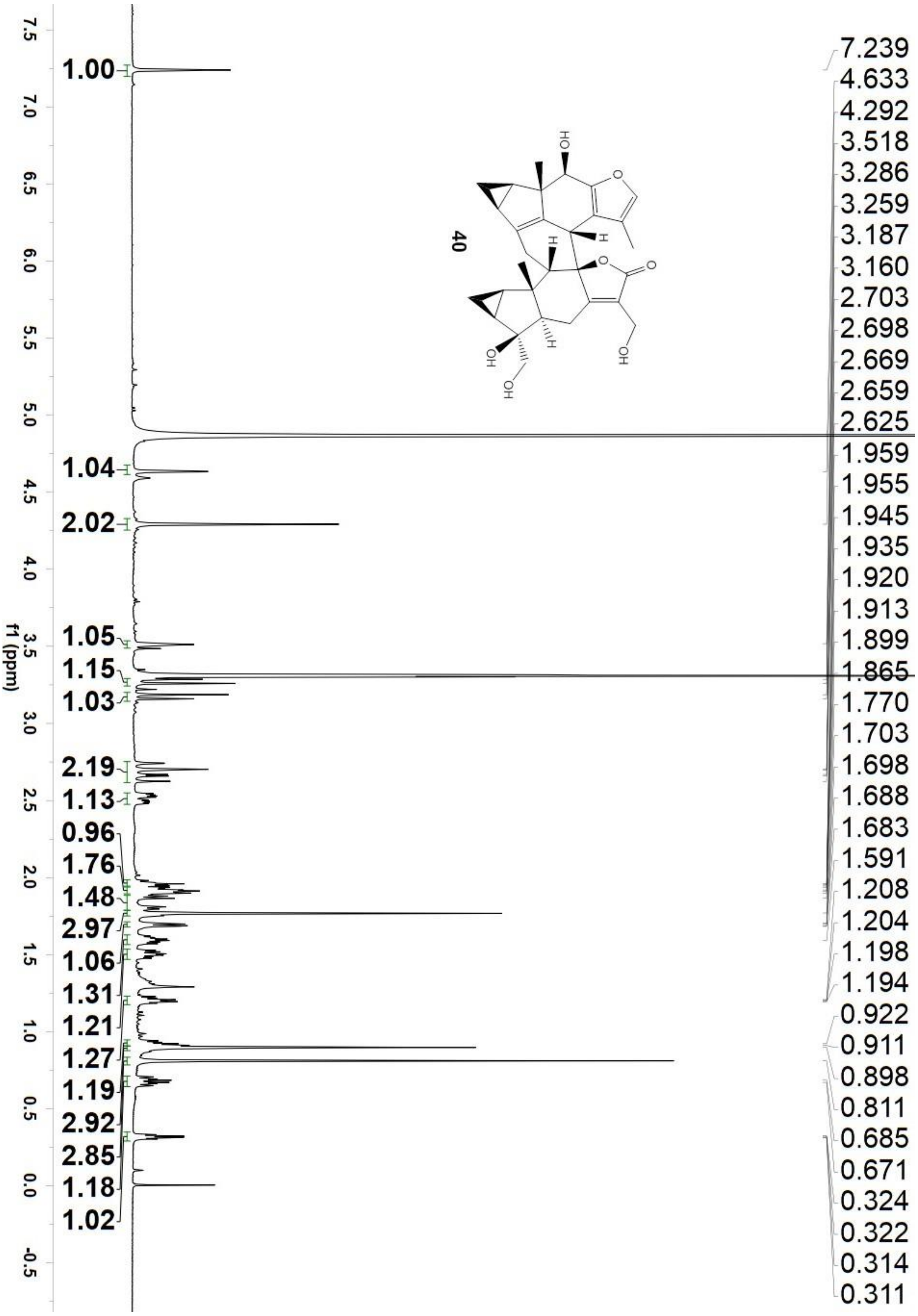

Supplementary Figure 65. ^{1}H NMR spectra of compound **40** in CD_3OD (400 MHz)

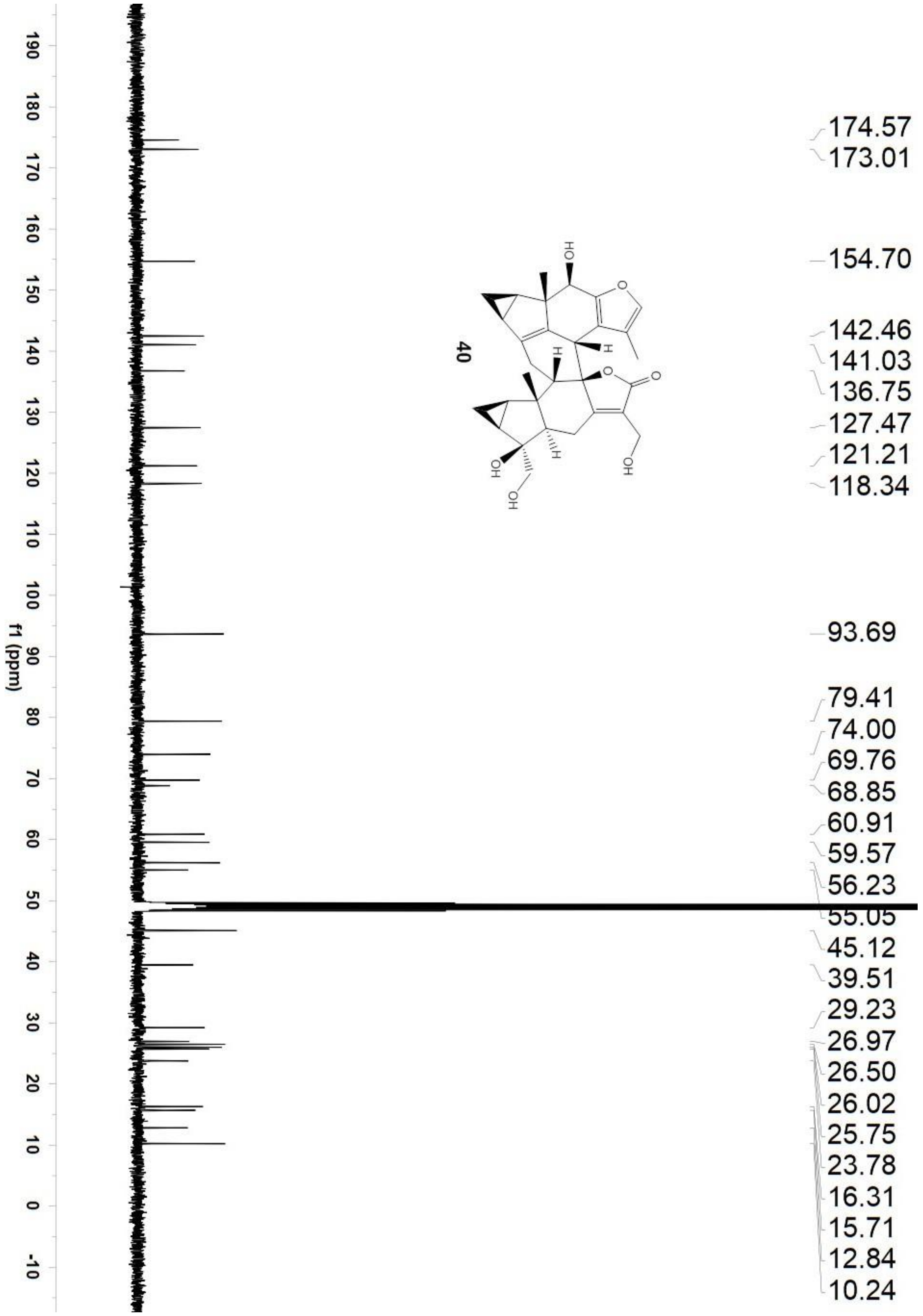

Supplementary Figure 66. ^{13}C NMR spectra of compound **40** in CD_3OD (100 MHz)

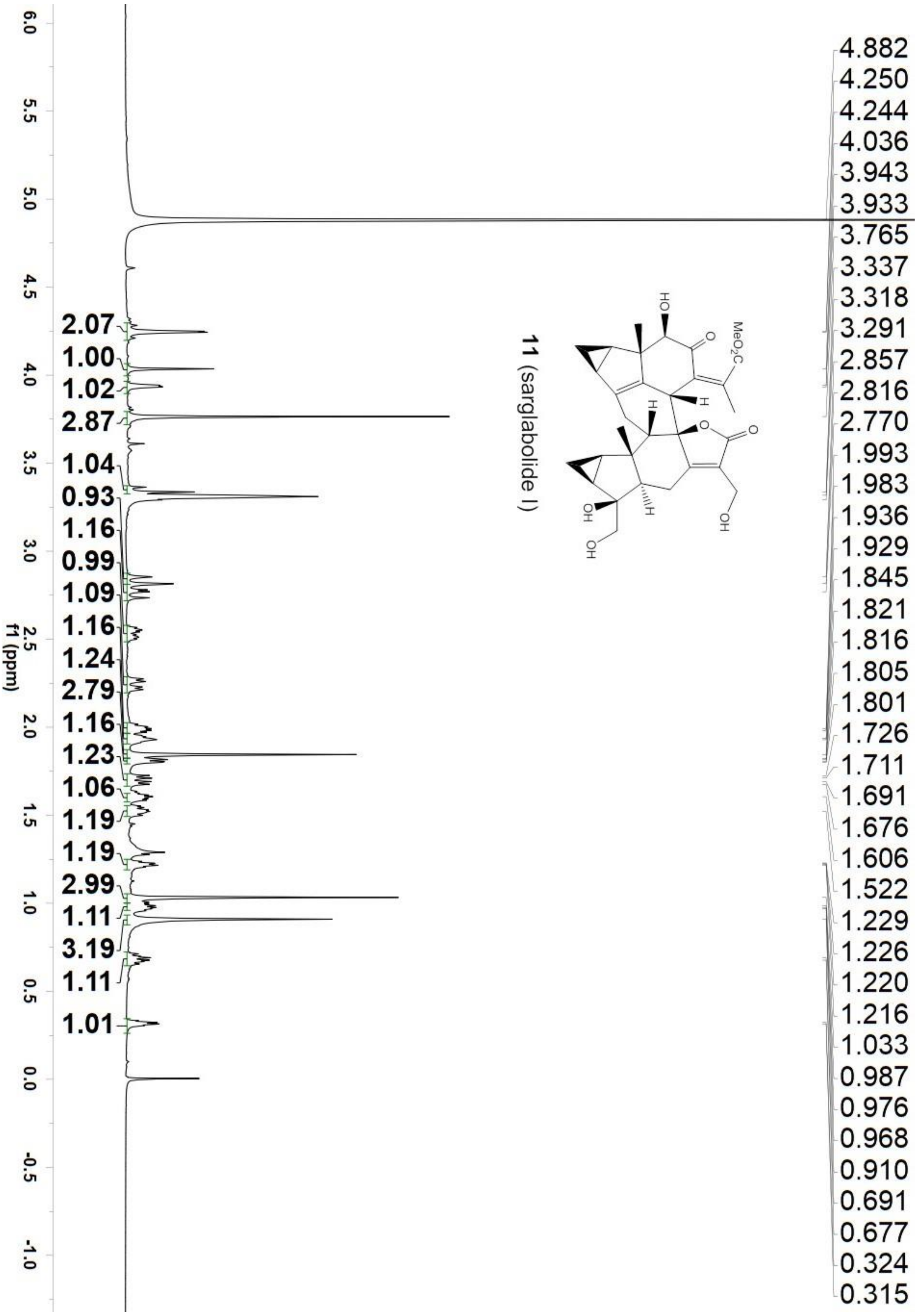

Supplementary Figure 67. ^{1}H NMR spectra of compound 11 in CD_3OD (400 MHz)

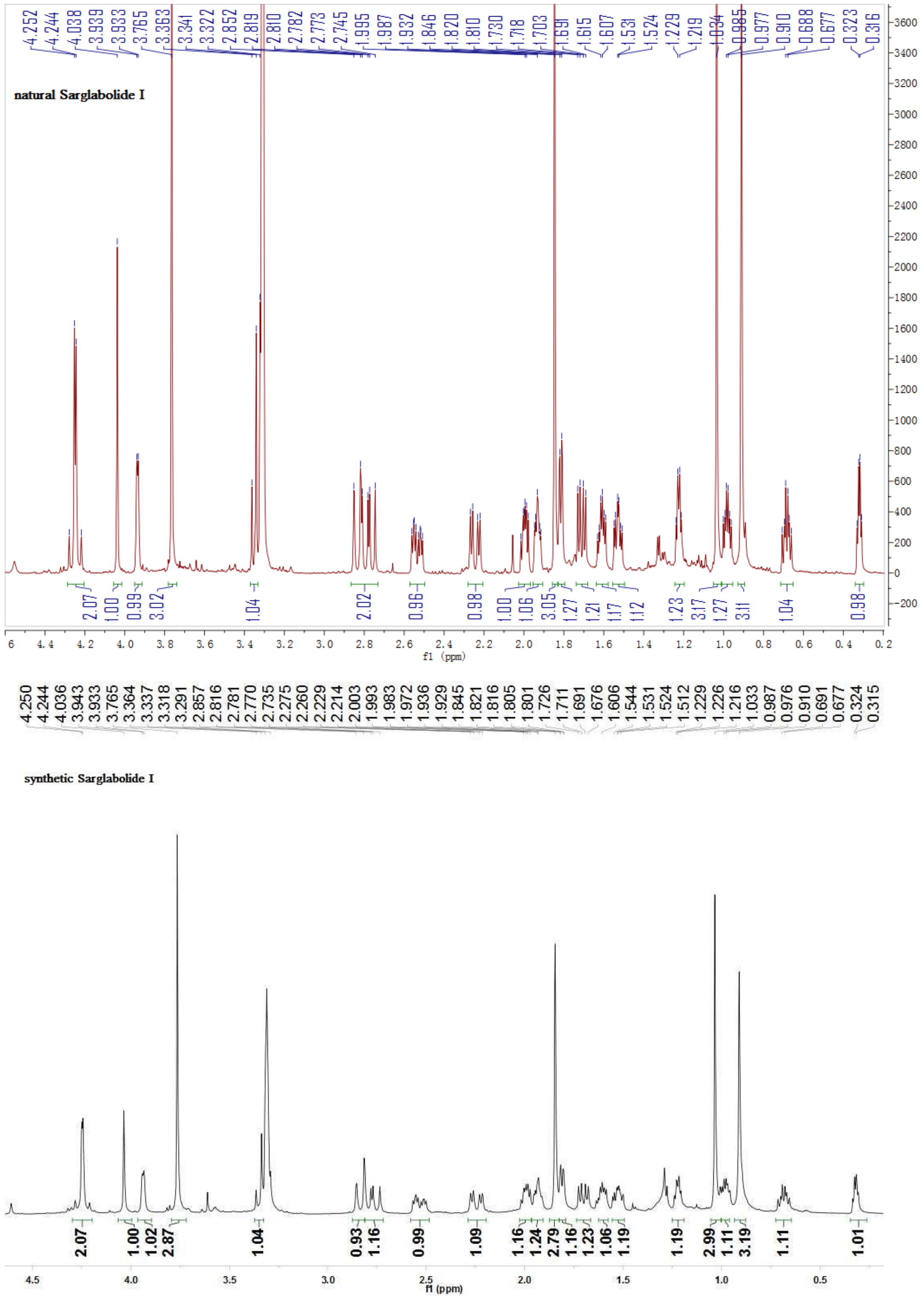

Supplementary Figure 68. Comparison of ^{1}H NMR data of natural Sarglabolide I[5](500 MHz) with those of synthetic Sarglabolide I (400 MHz)

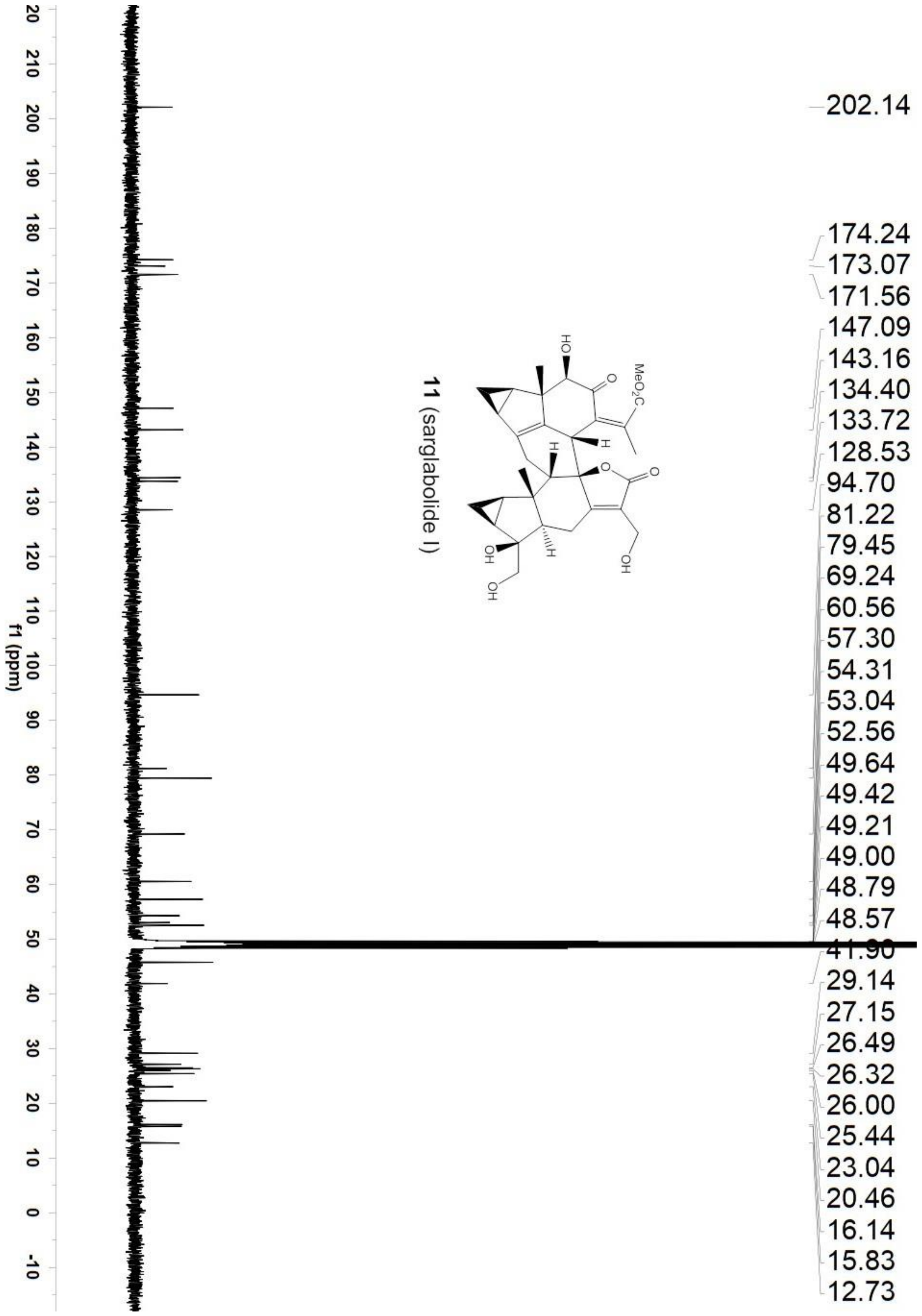

Supplementary Figure 69. ^{13}C NMR spectra of compound **11** in CD_3OD (100 MHz)

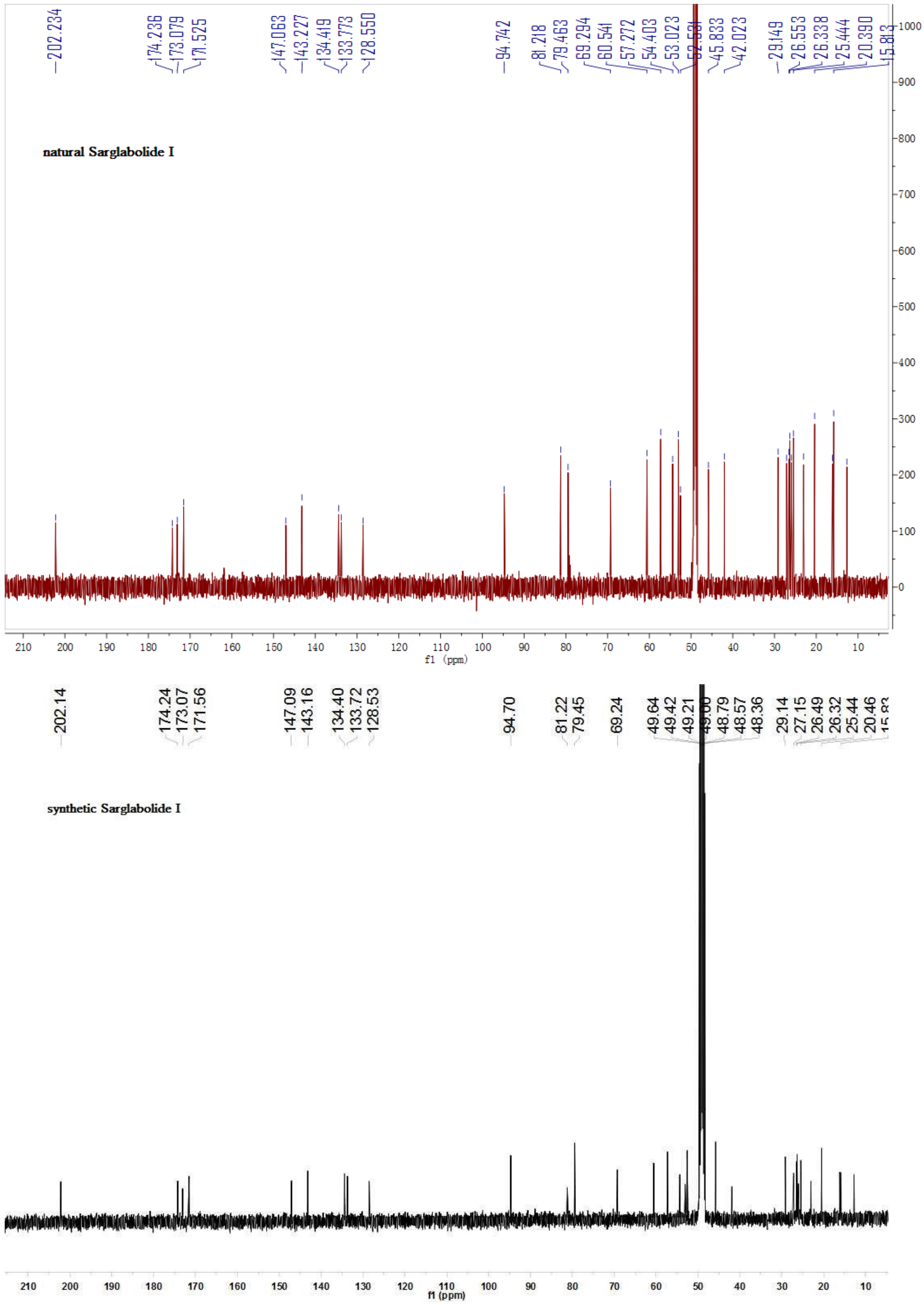

Supplementary Figure 70. Comparison of ^{13}C NMR data of natural Sarglabolide I[5] (125 MHz) with those of synthetic Sarglabolide I (100 MHz)

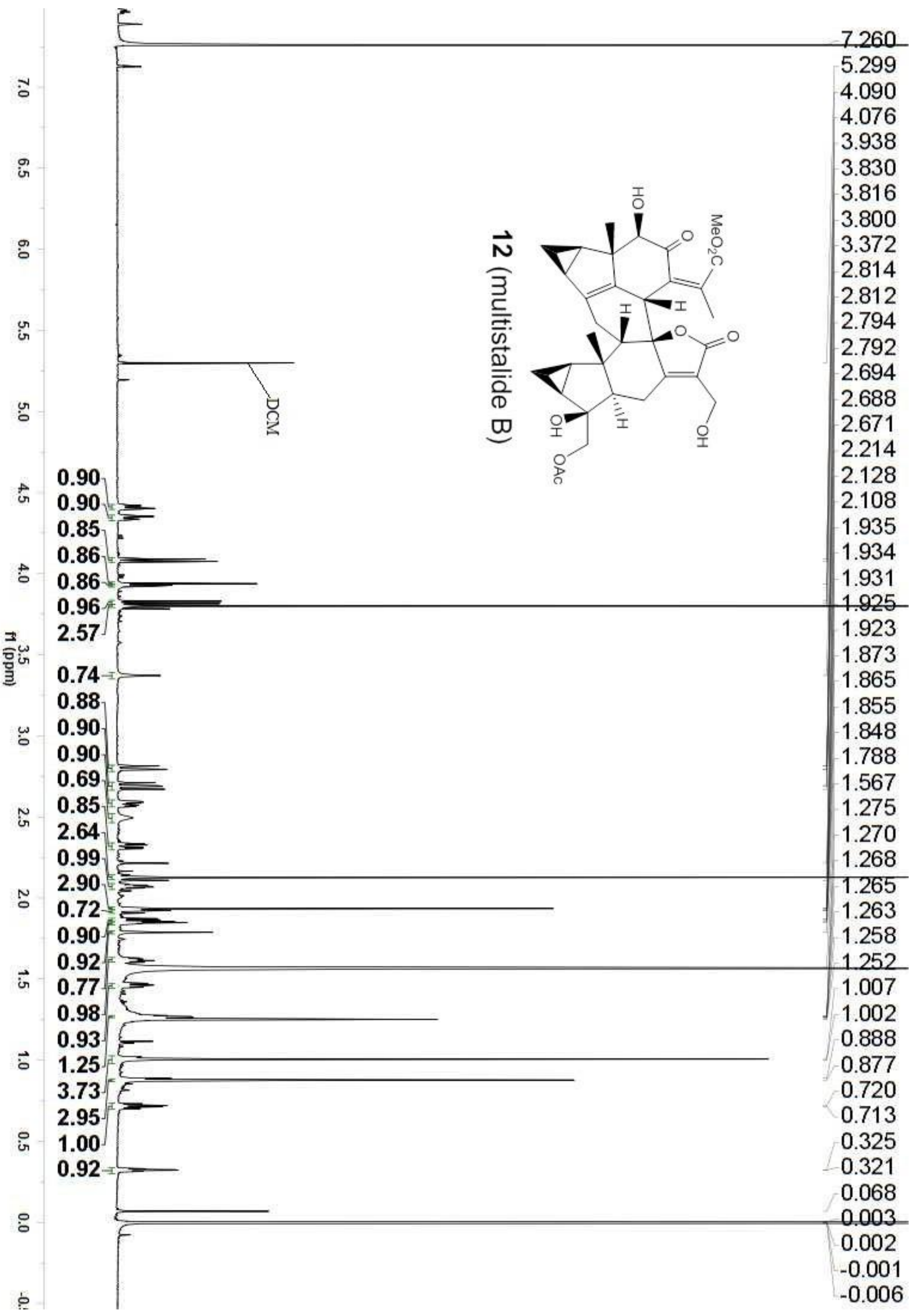

Supplementary Figure 71. ^{1}H NMR spectra of compound **12** in $CDCl_3$ (800 MHz)

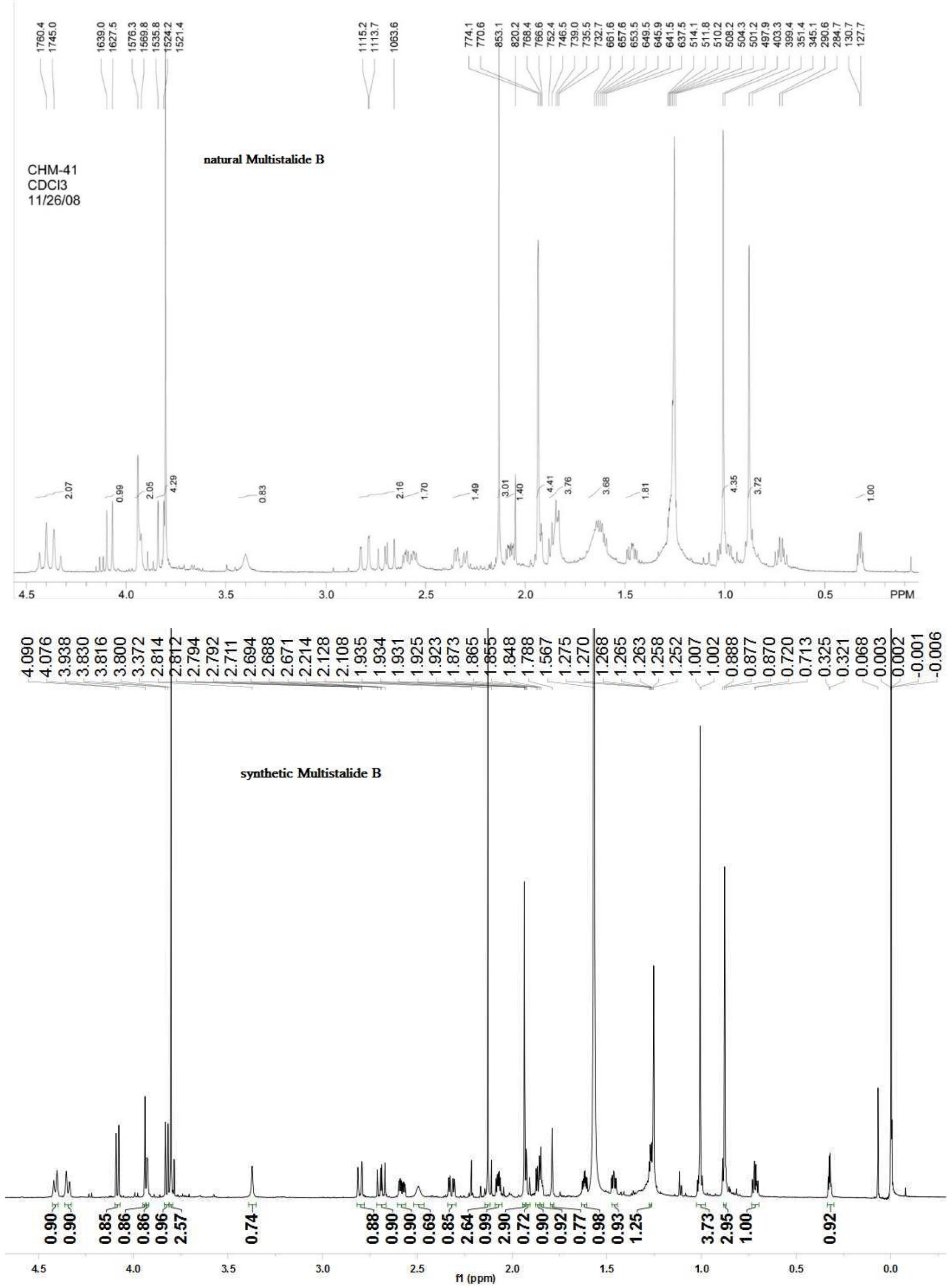

Supplementary Figure 72. Comparison of ^{1}H NMR data of natural Multistalide B[6](400 MHz) with those of synthetic Multistalide B (800 MHz)

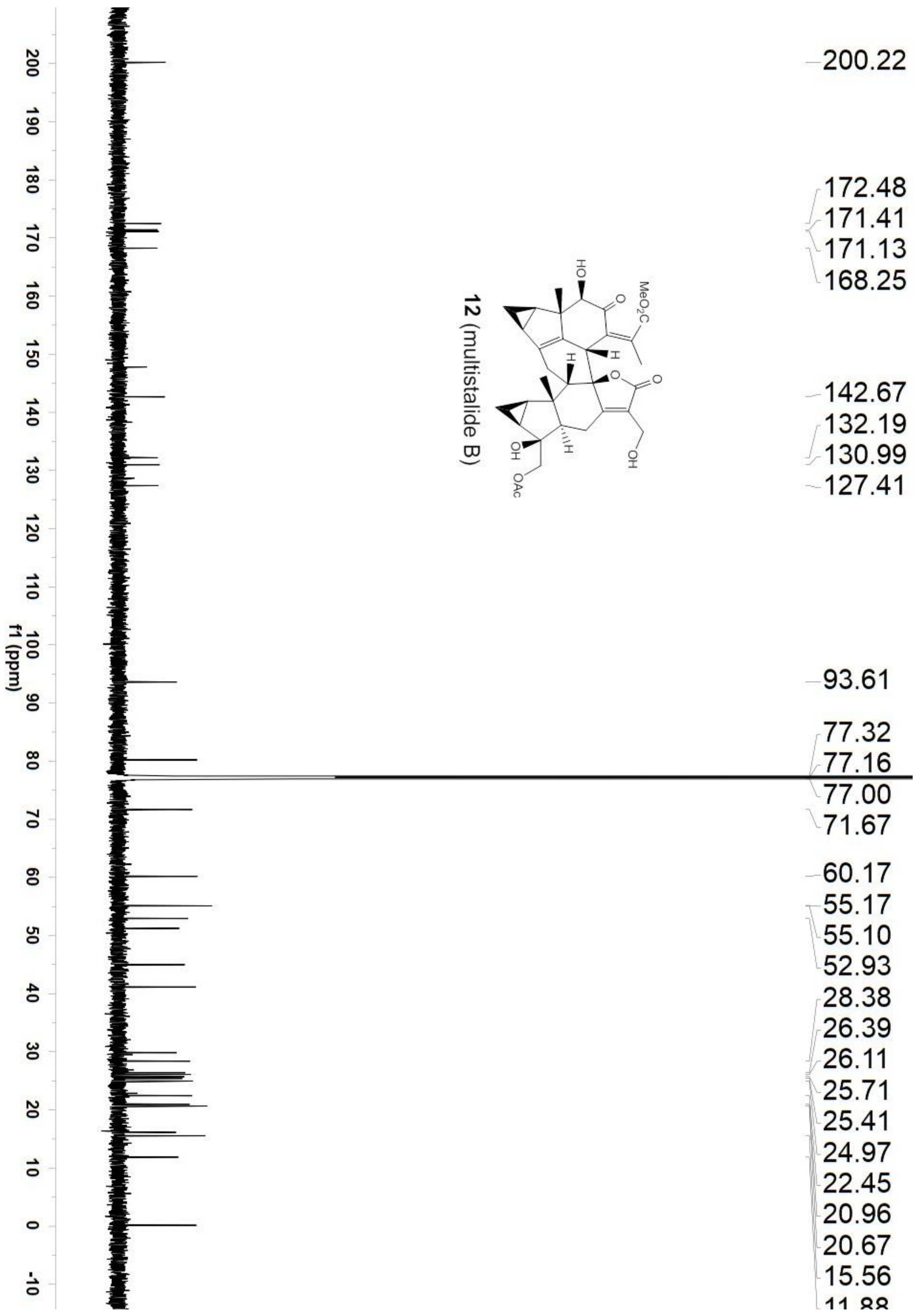

Supplementary Figure 73. ^{13}C NMR spectra of compound 12 in $CDCl_3$ (200 MHz)

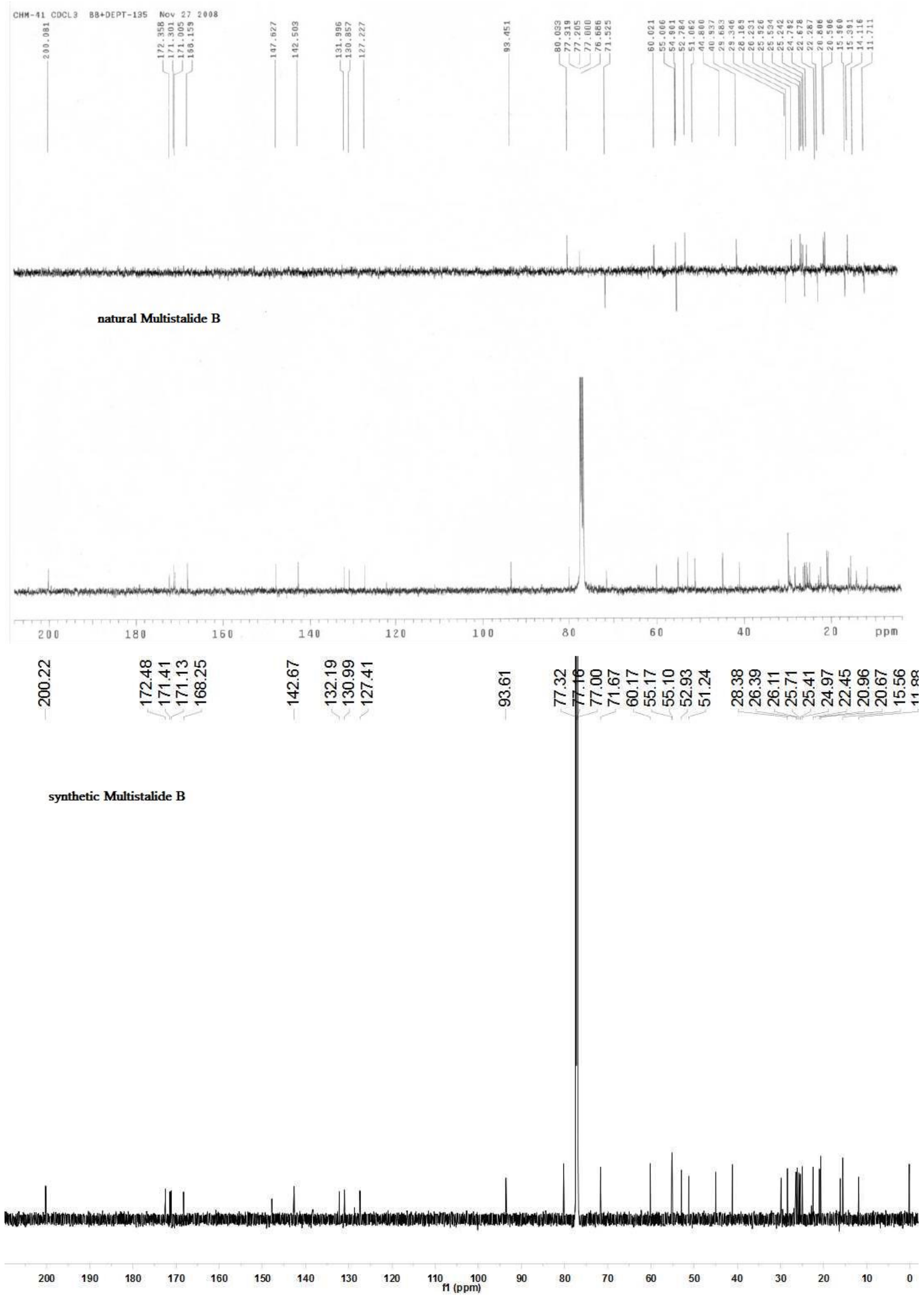

Supplementary Figure 74. Comparison of ^{13}C NMR data of natural Multistalide B[6] (100 MHz)with those of synthetic Multistalide B (200 MHz)

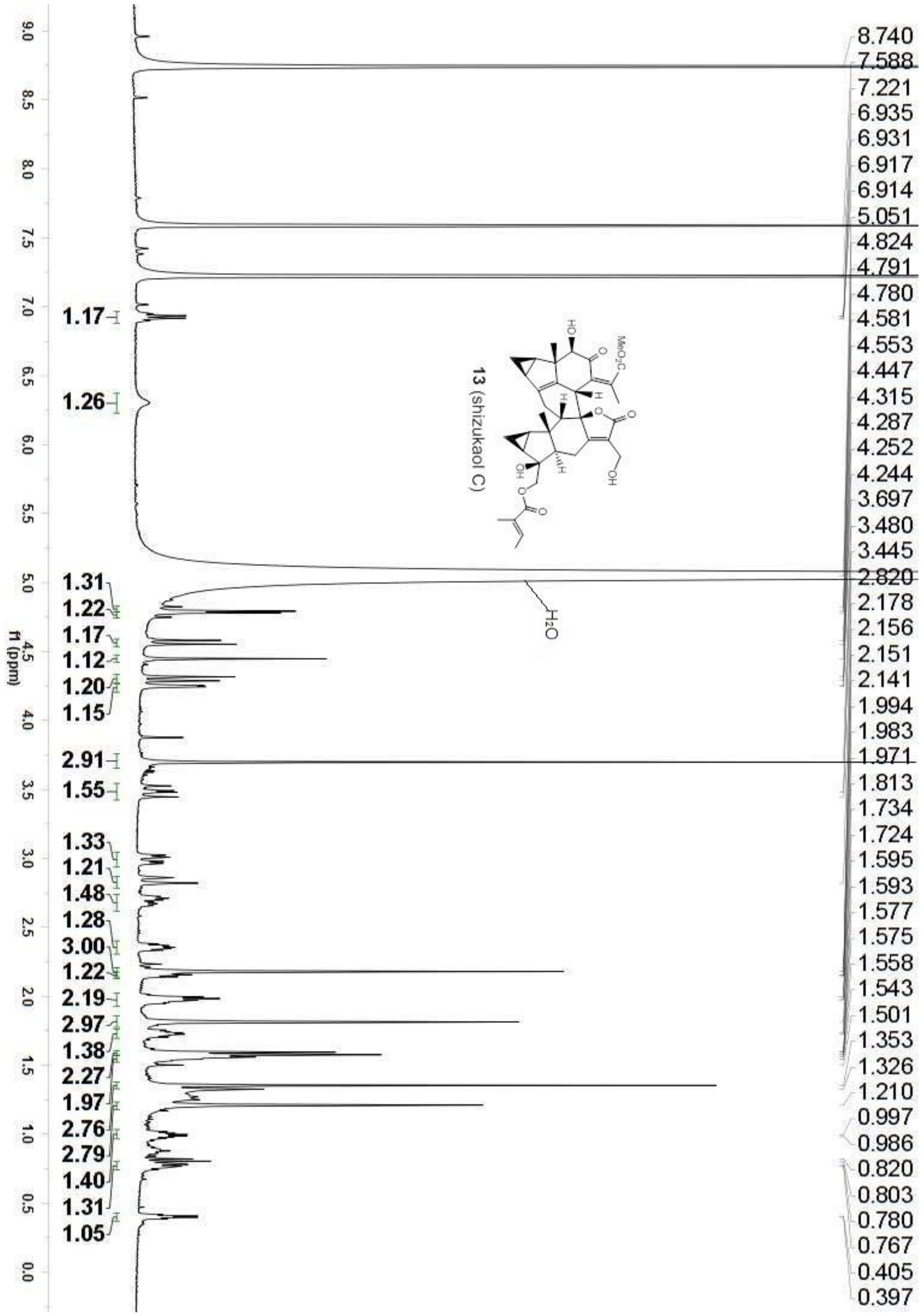

Supplementary Figure 75. ^{1}H NMR spectra of compound **13** in Pyridine-d_5 (400 MHz)

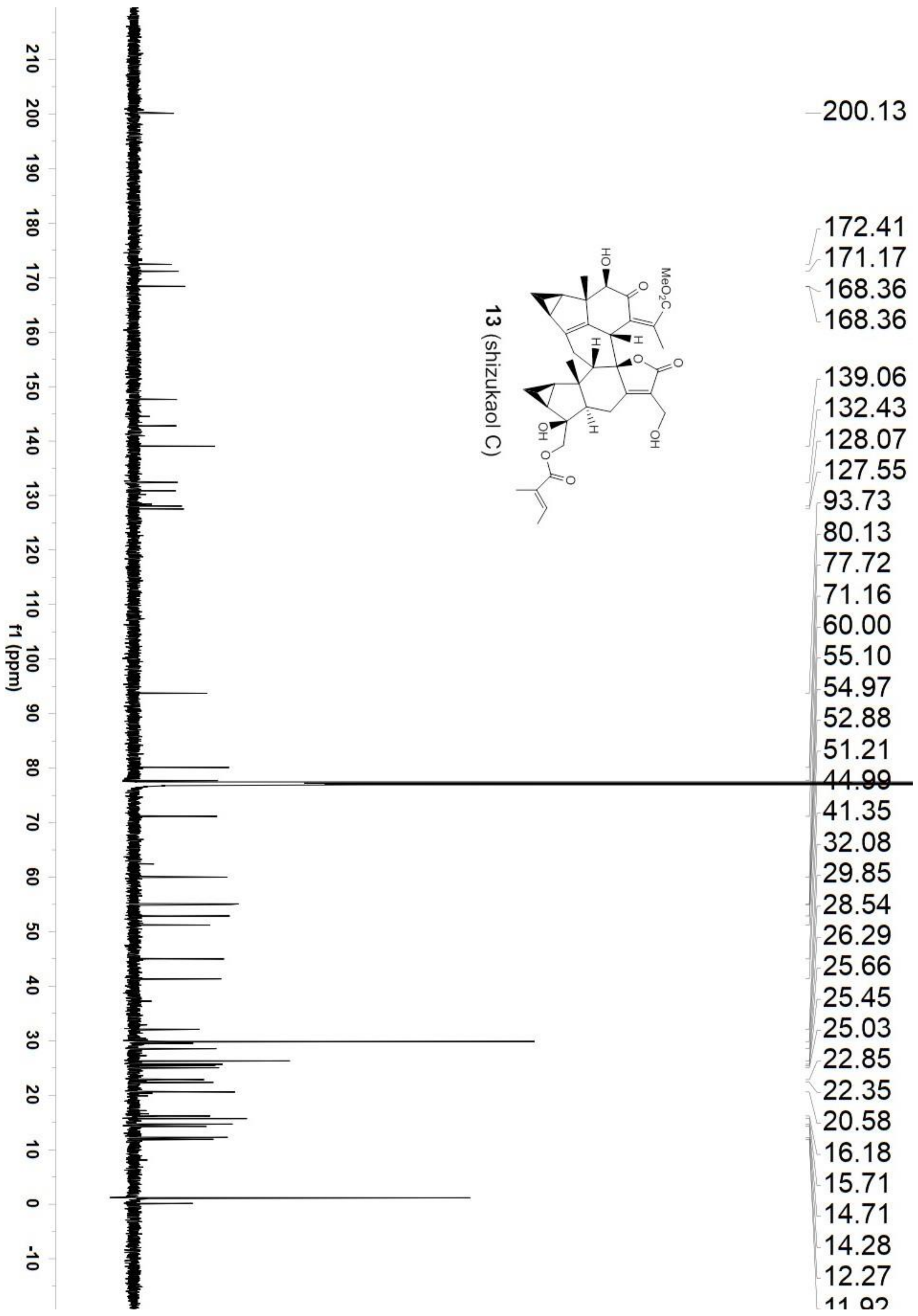

Supplementary Figure 76. ^{13}C NMR spectra of compound **13** in $CDCl_3$ (100 MHz)

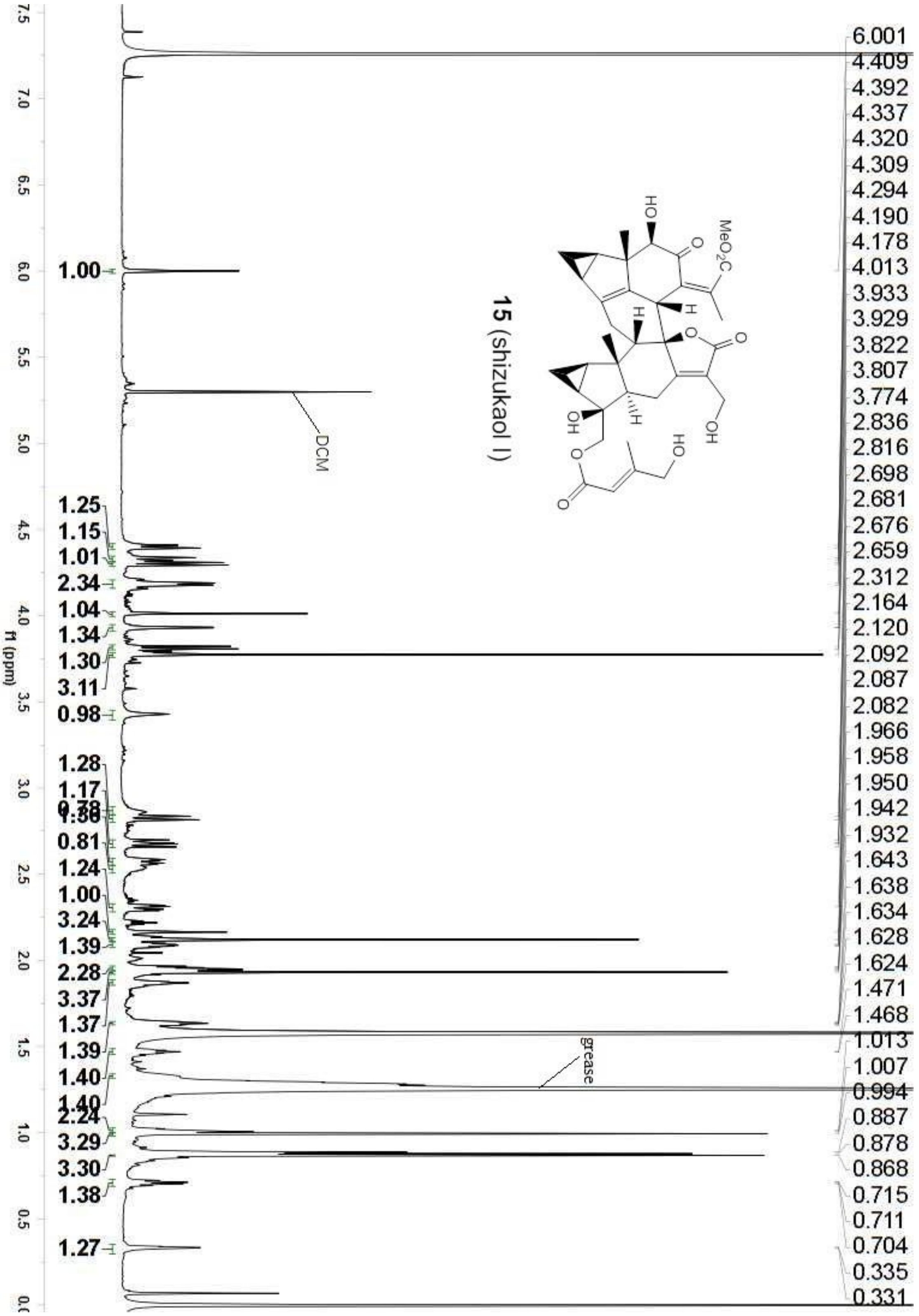

Supplementary Figure 77. ^{1}H NMR spectra of compound **15** in $CDCl_3$ (800 MHz)

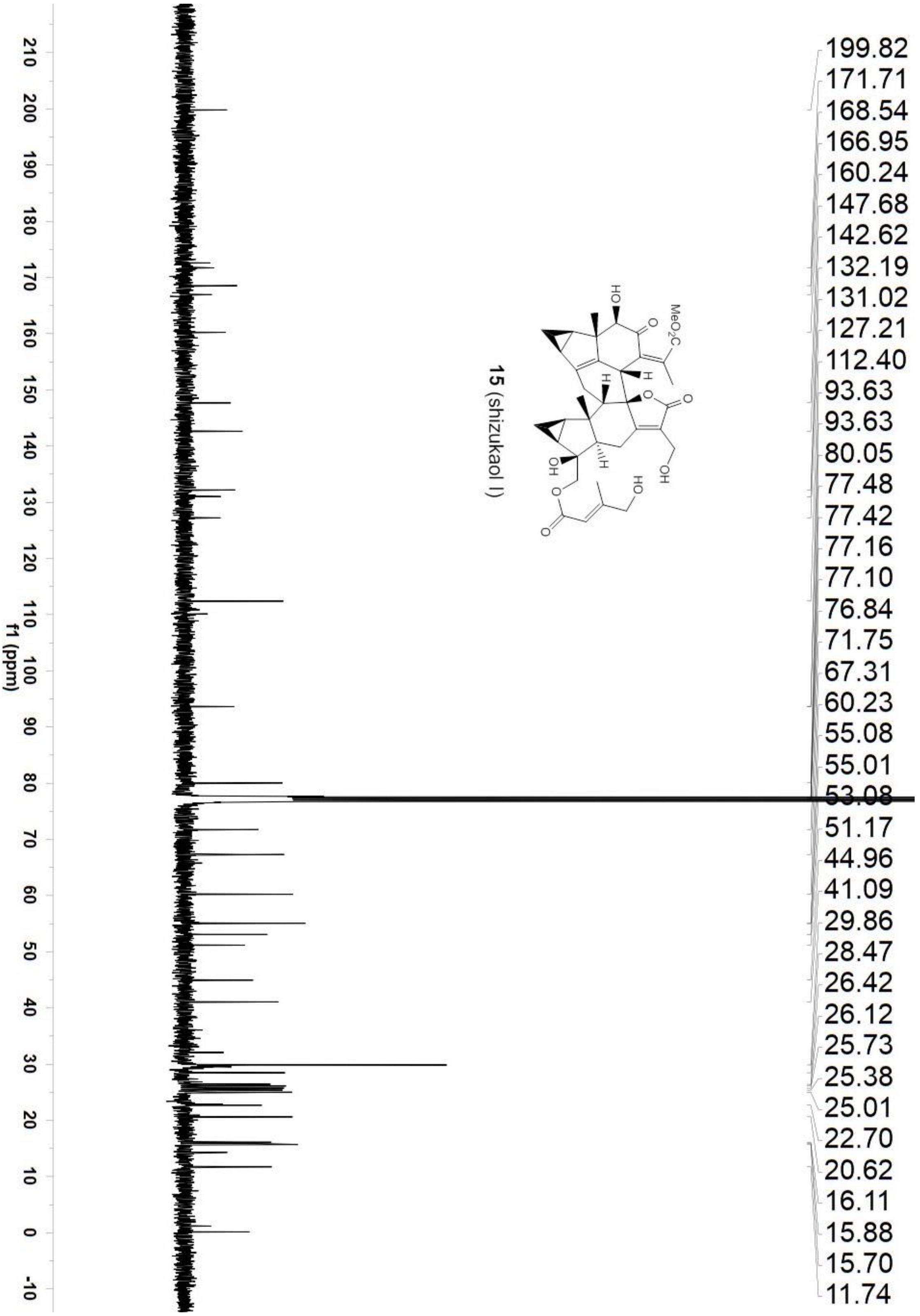

Supplementary Figure 78. ^{13}C NMR spectra of compound **15** in $CDCl_3$ (100 MHz)

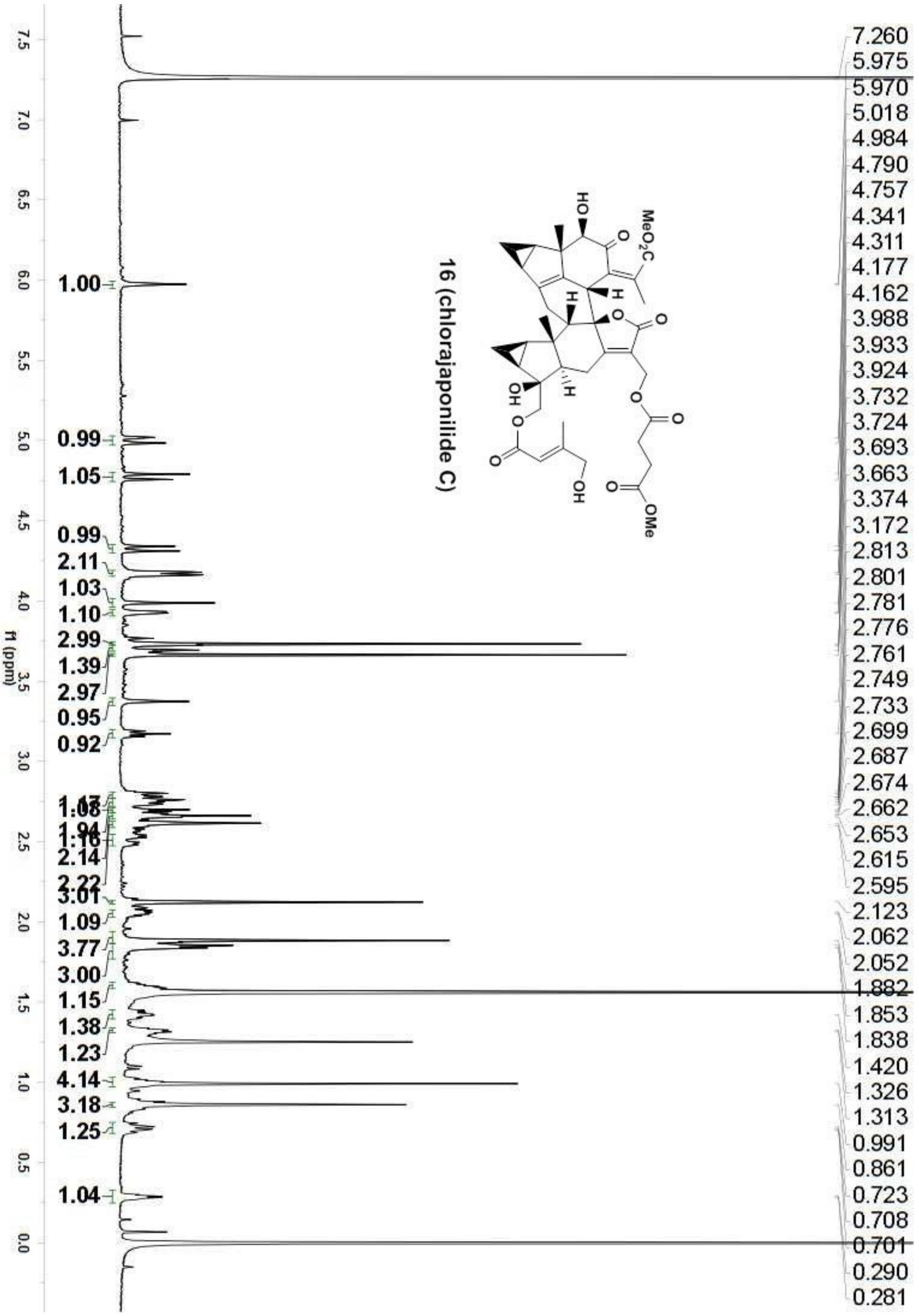

Supplementary Figure 79. ^{1}H NMR spectra of compound **16** in $CDCl_3$ (400 MHz)

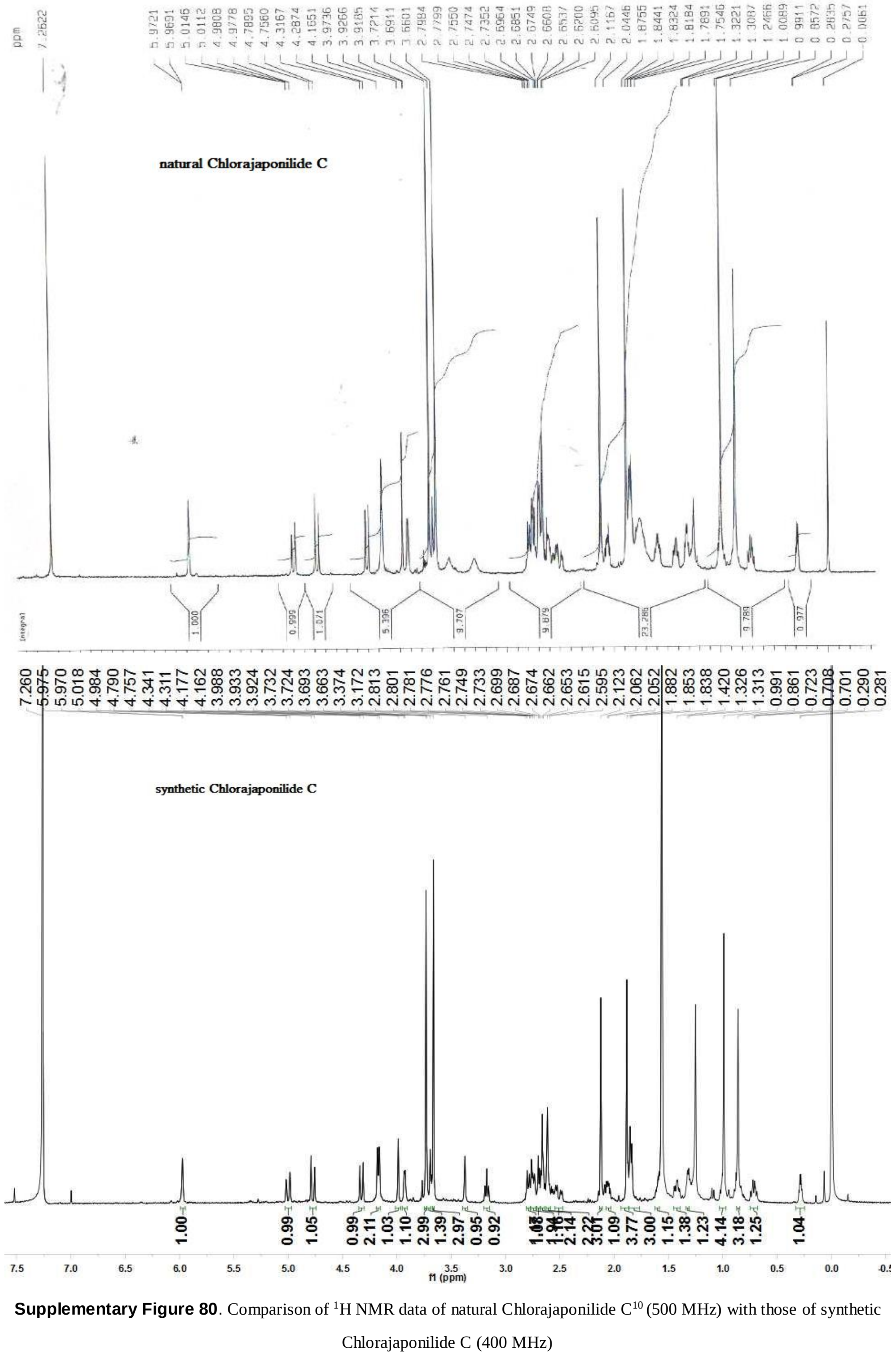

Supplementary Figure 80. Comparison of ^{1}H NMR data of natural Chlorajaponilide C[10] (500 MHz) with those of synthetic Chlorajaponilide C (400 MHz)

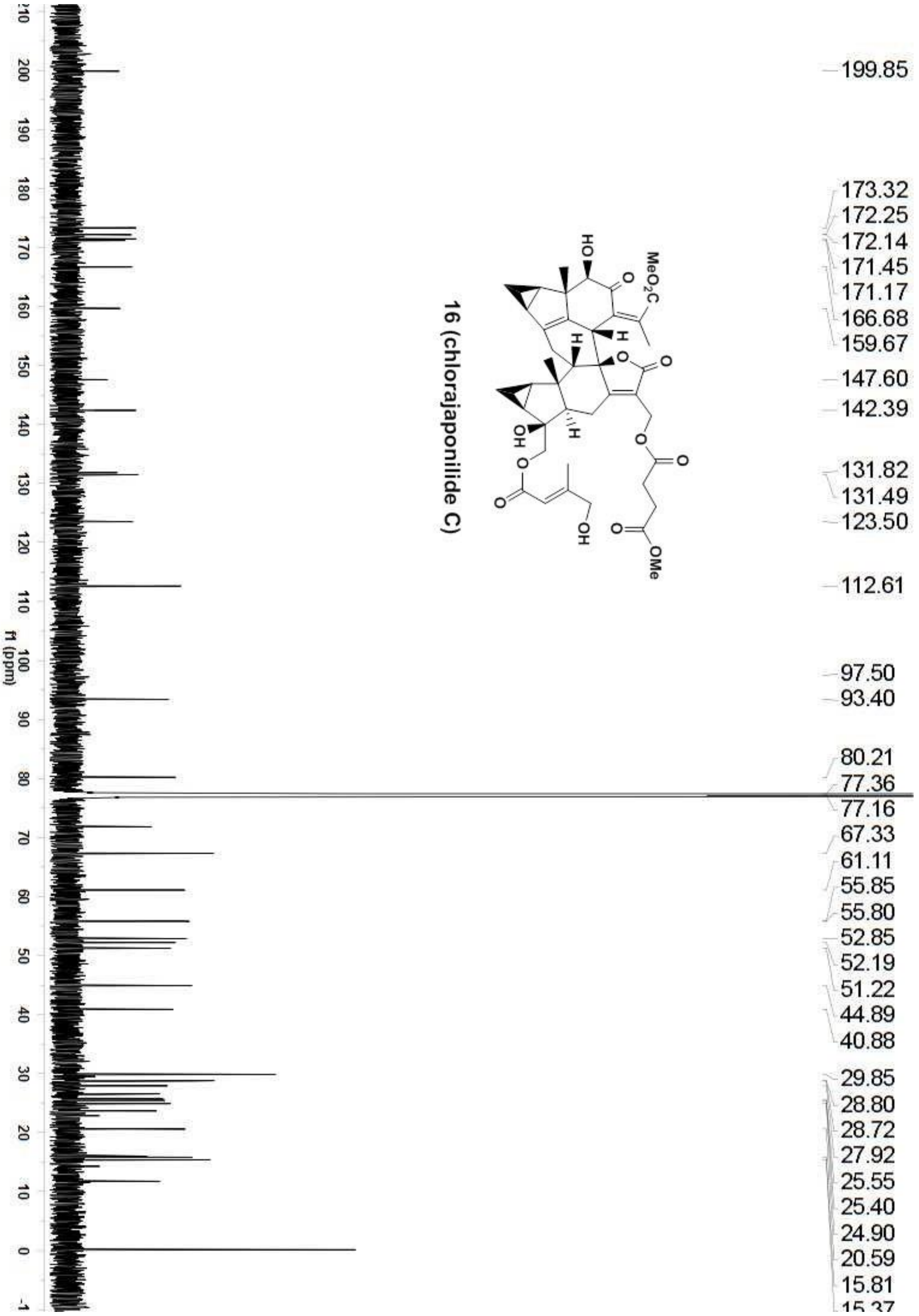

Supplementary Figure 81. ^{13}C NMR spectra of compound **16** in $CDCl_3$ (200 MHz)

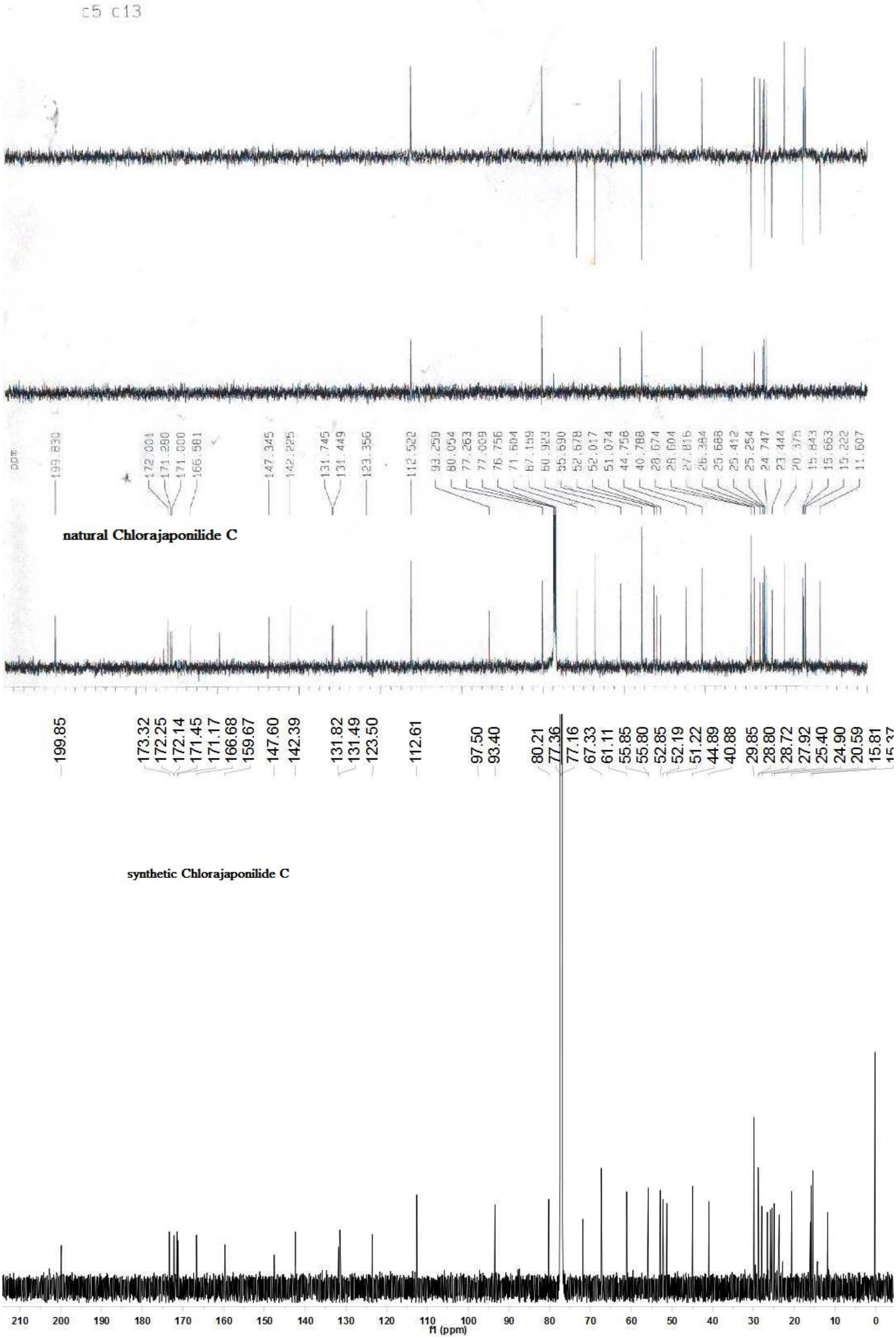

Supplementary Figure 82. Comparison of ^{13}C NMR data of natural Chlorajaponilide C [10] (125 MHz) with those of synthetic Chlorajaponilide C (200 MHz)

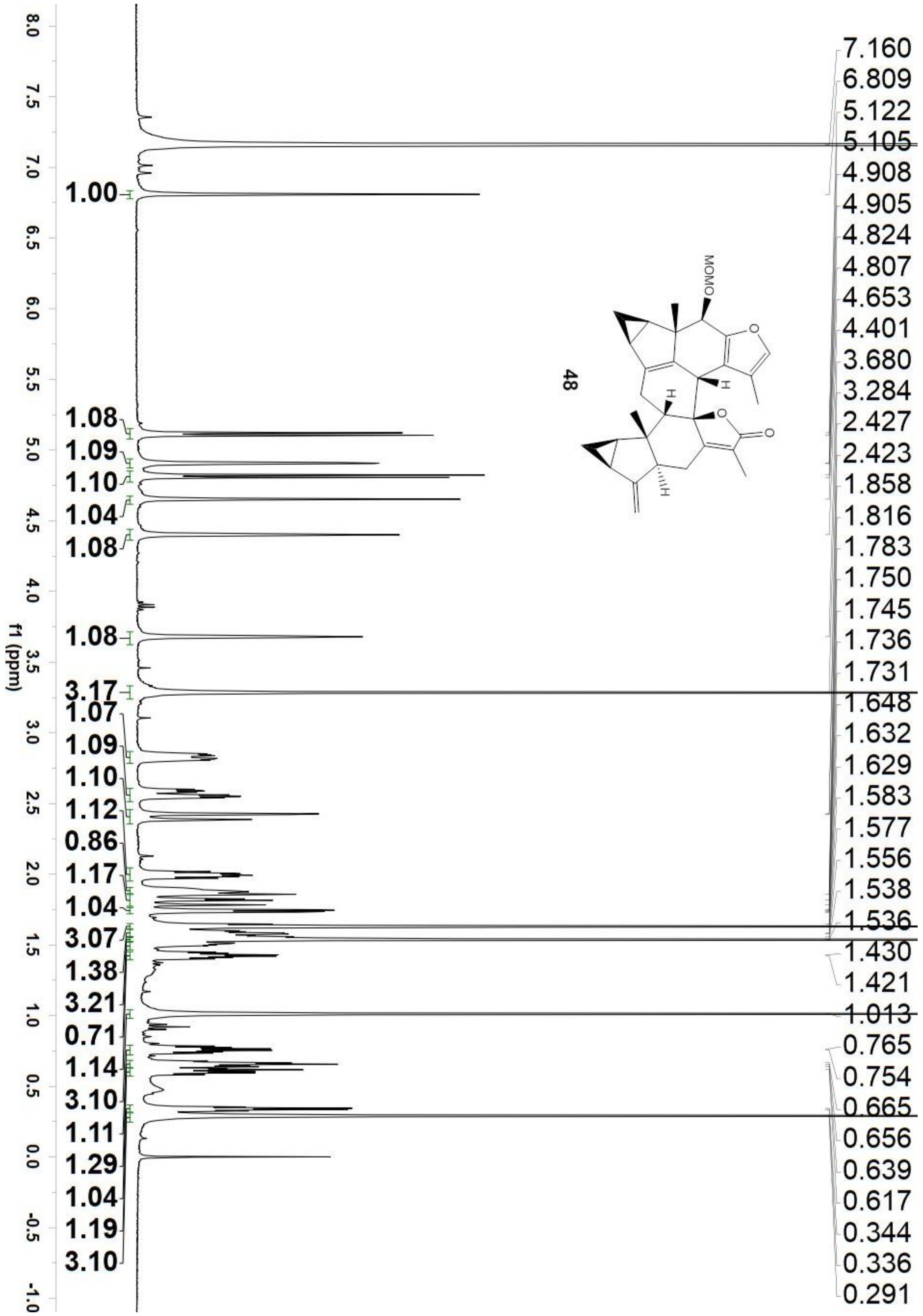

Supplementary Figure 83. ^{1}H NMR spectra of compound **48** in C_6D_6 (400 MHz)

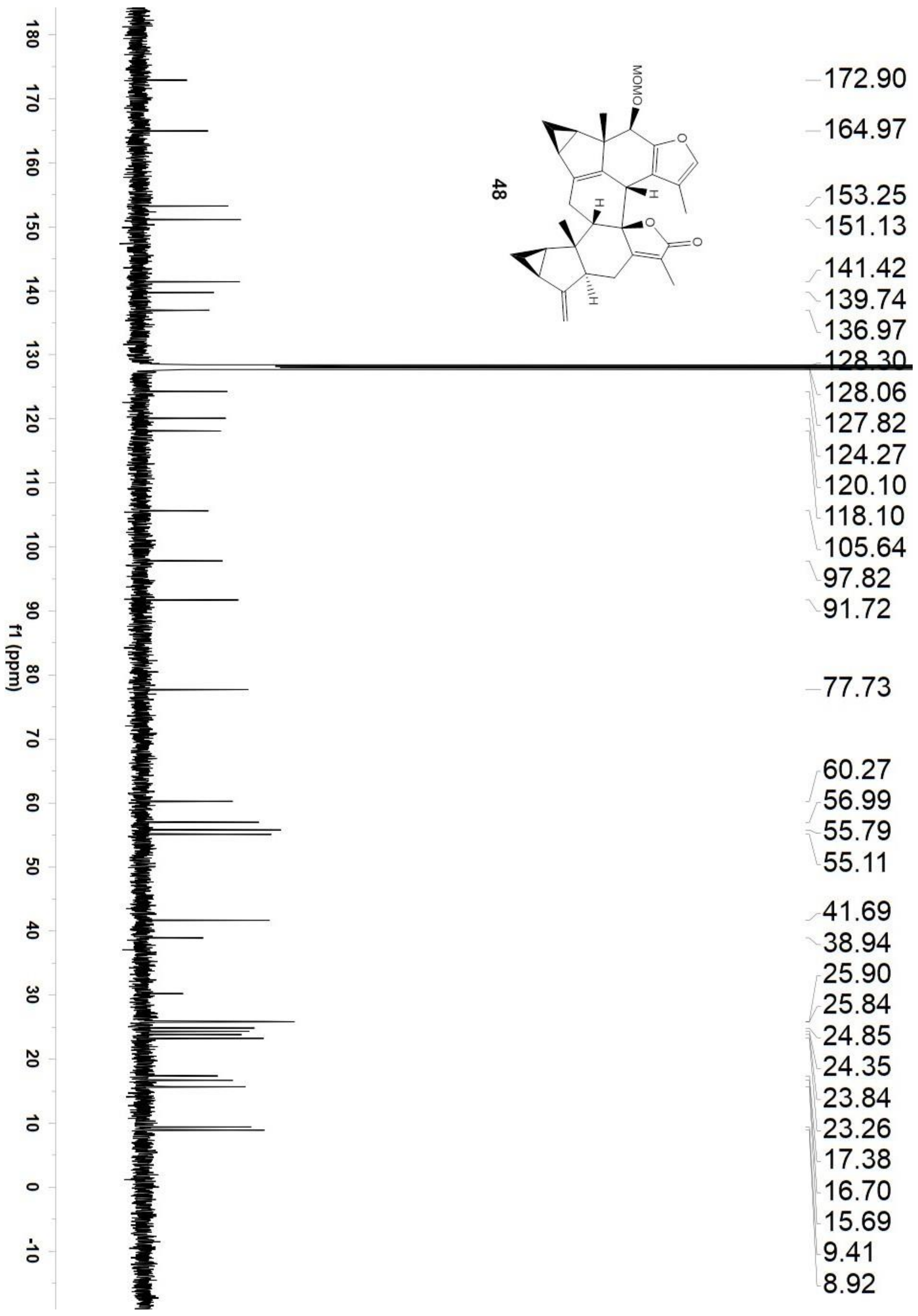

Supplementary Figure 84. 13C NMR spectra of compound **48** in C6D6 (100 MHz)

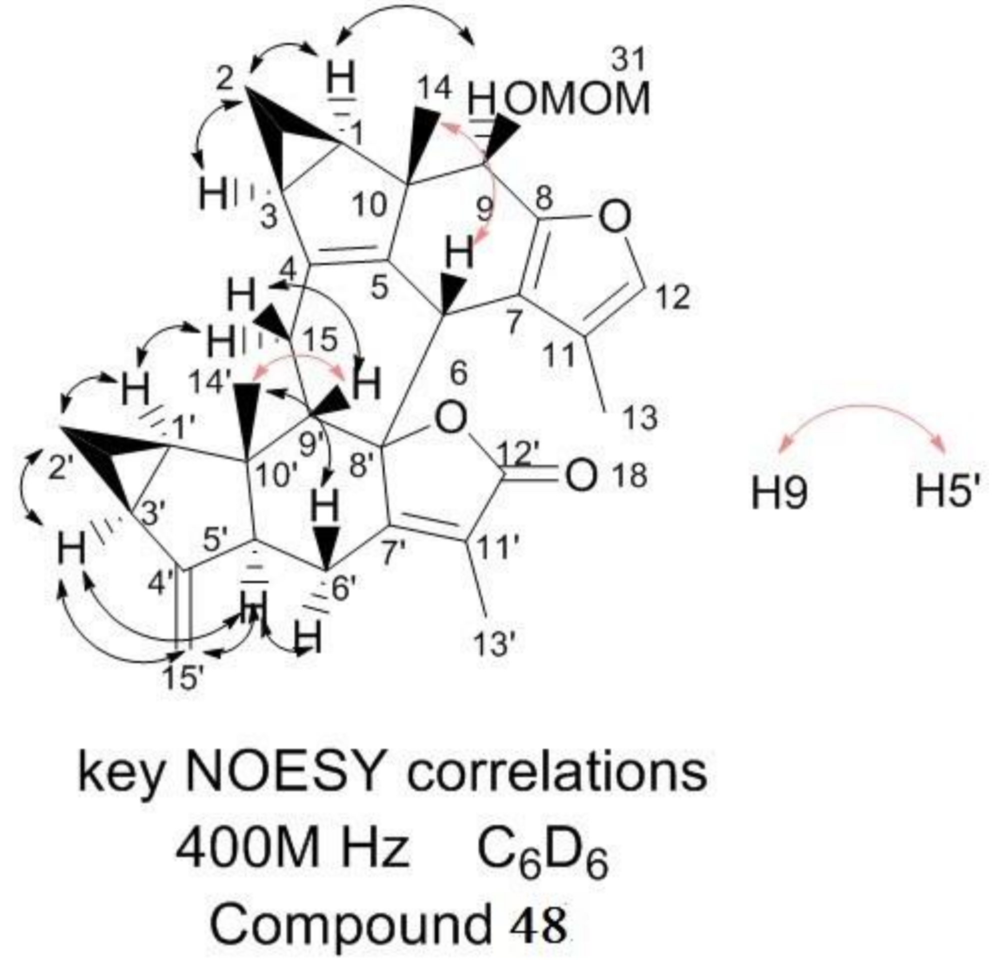

Supplementary Figure 85. Key NOESY correlations of compound **48**

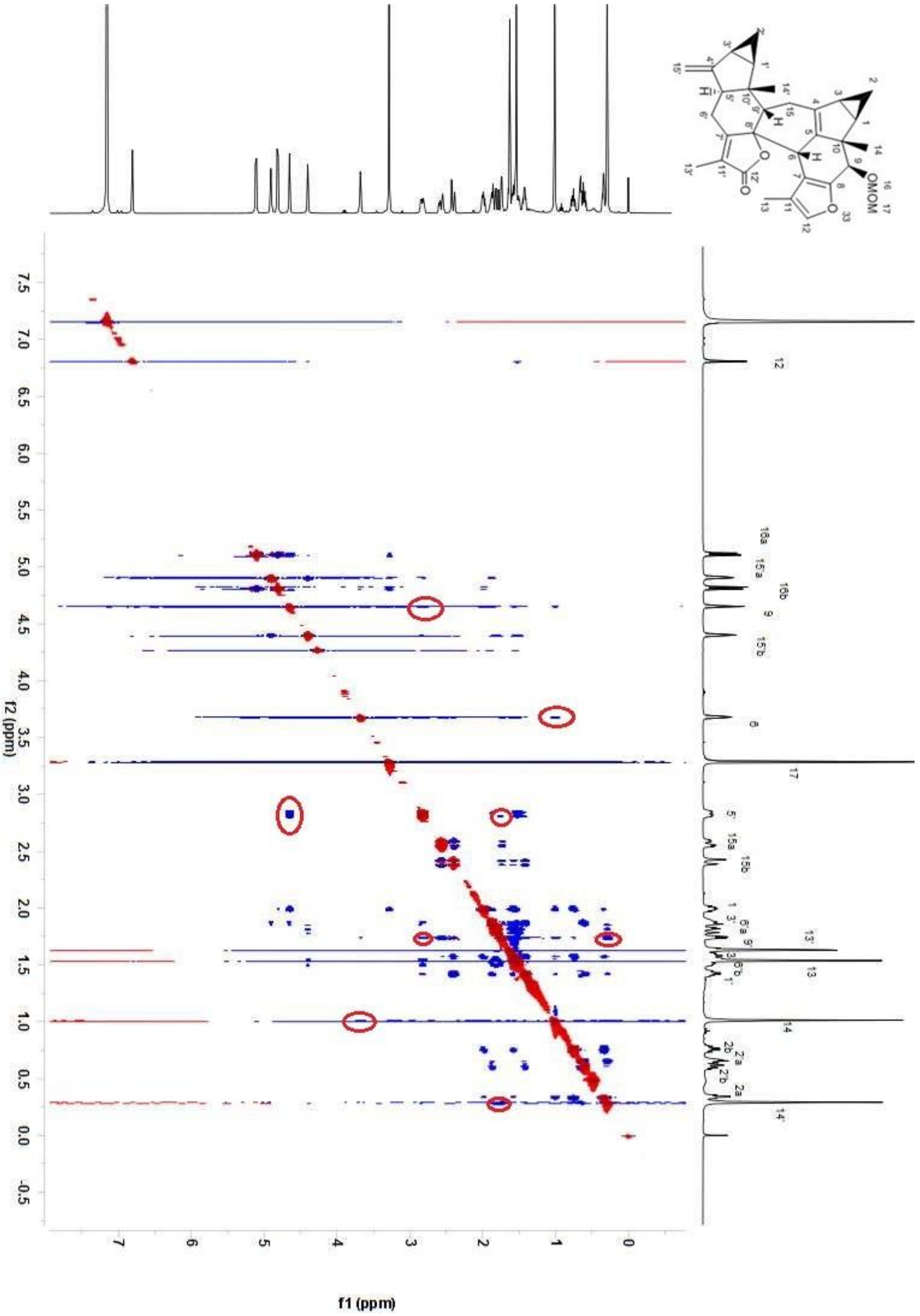

Supplementary Figure 86. NOESY NMR spectra of compound **48** in C_6D_6 (400 MHz)

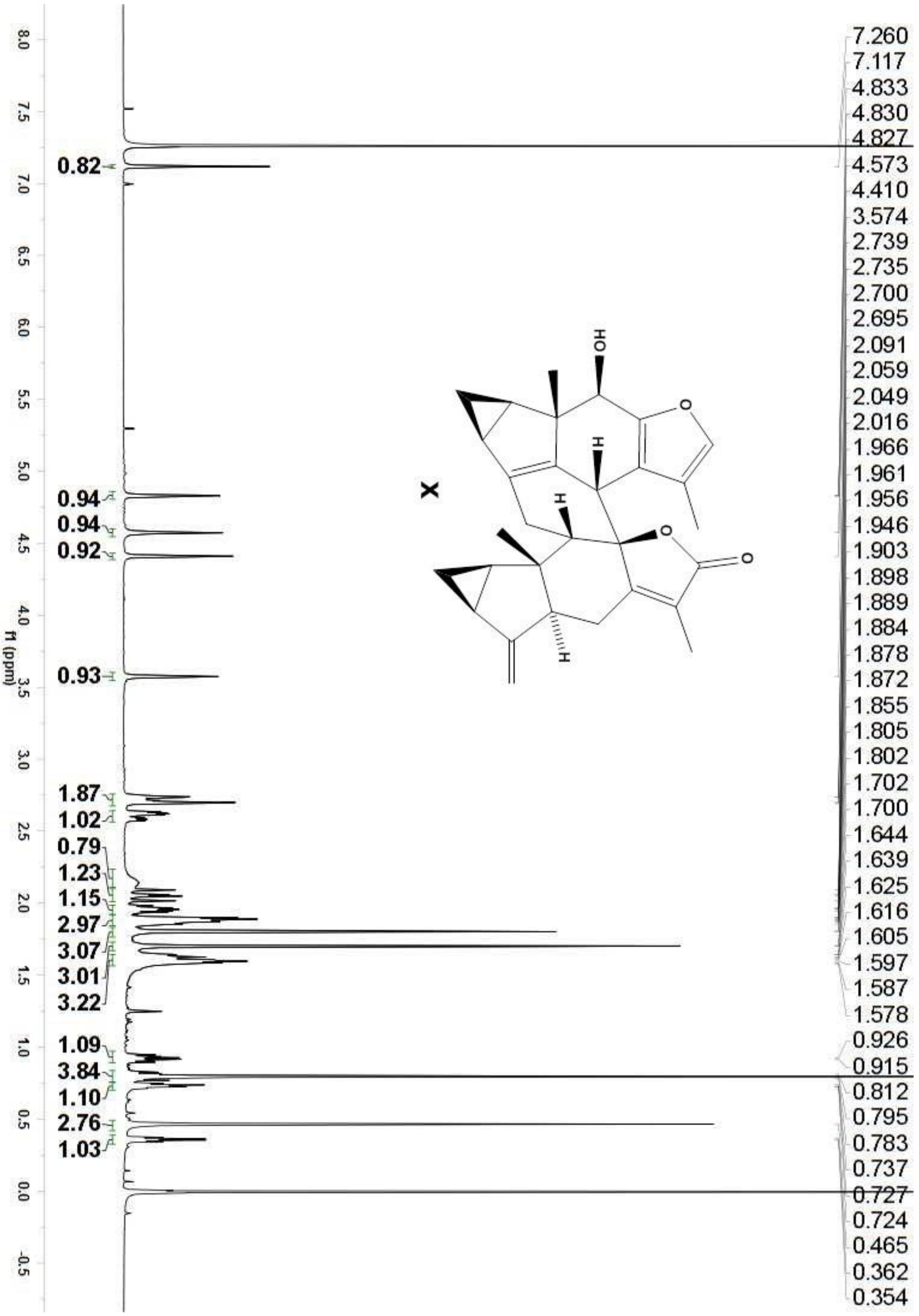

Supplementary Figure 87. ^{1}H NMR spectra of compound **x** in $CDCl_3$ (400 MHz)

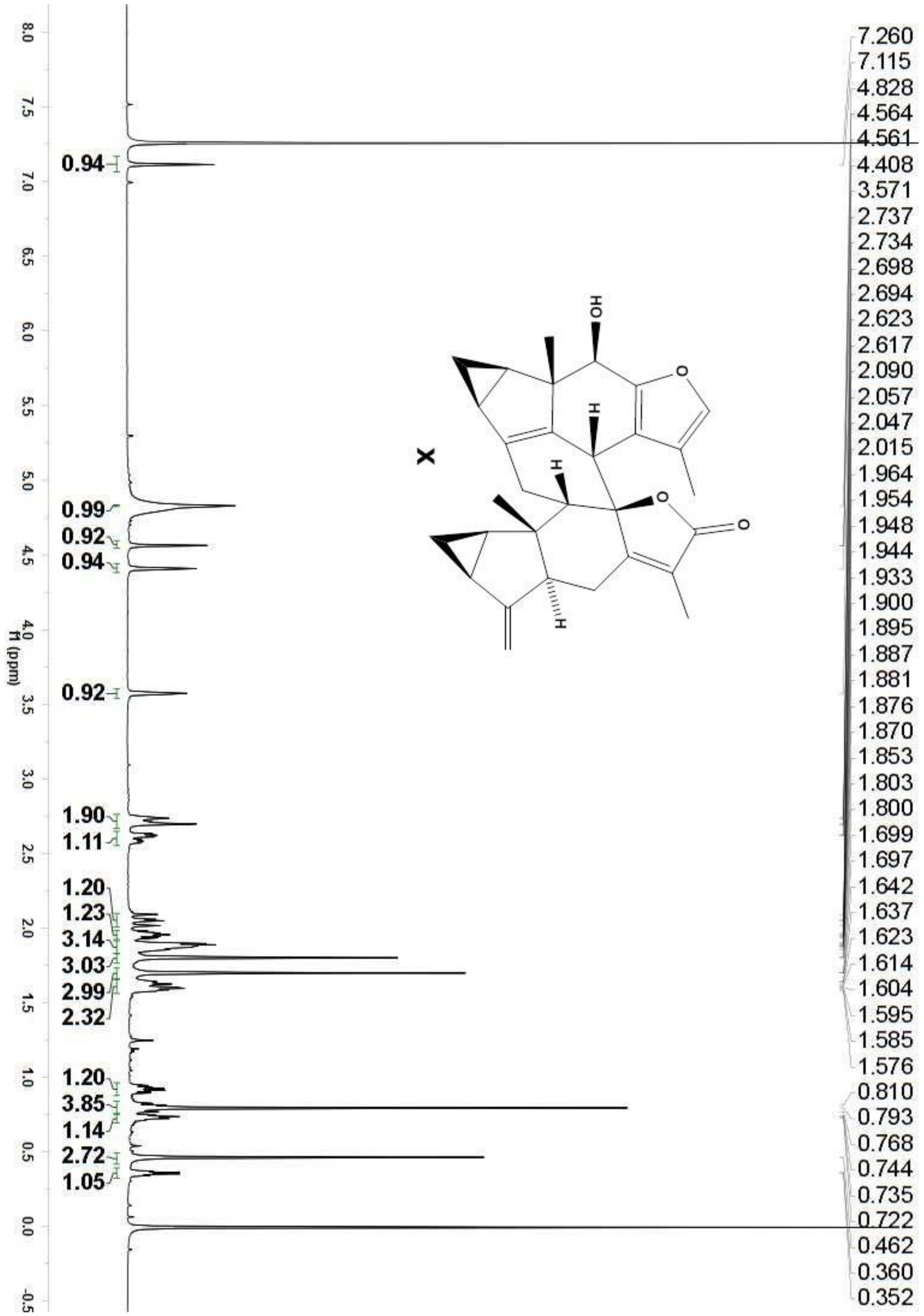

Supplementary Figure 88. ^{1}H NMR spectra of compound **x** in $CDCl_3$ with a drop of D_2O (400 MHz)

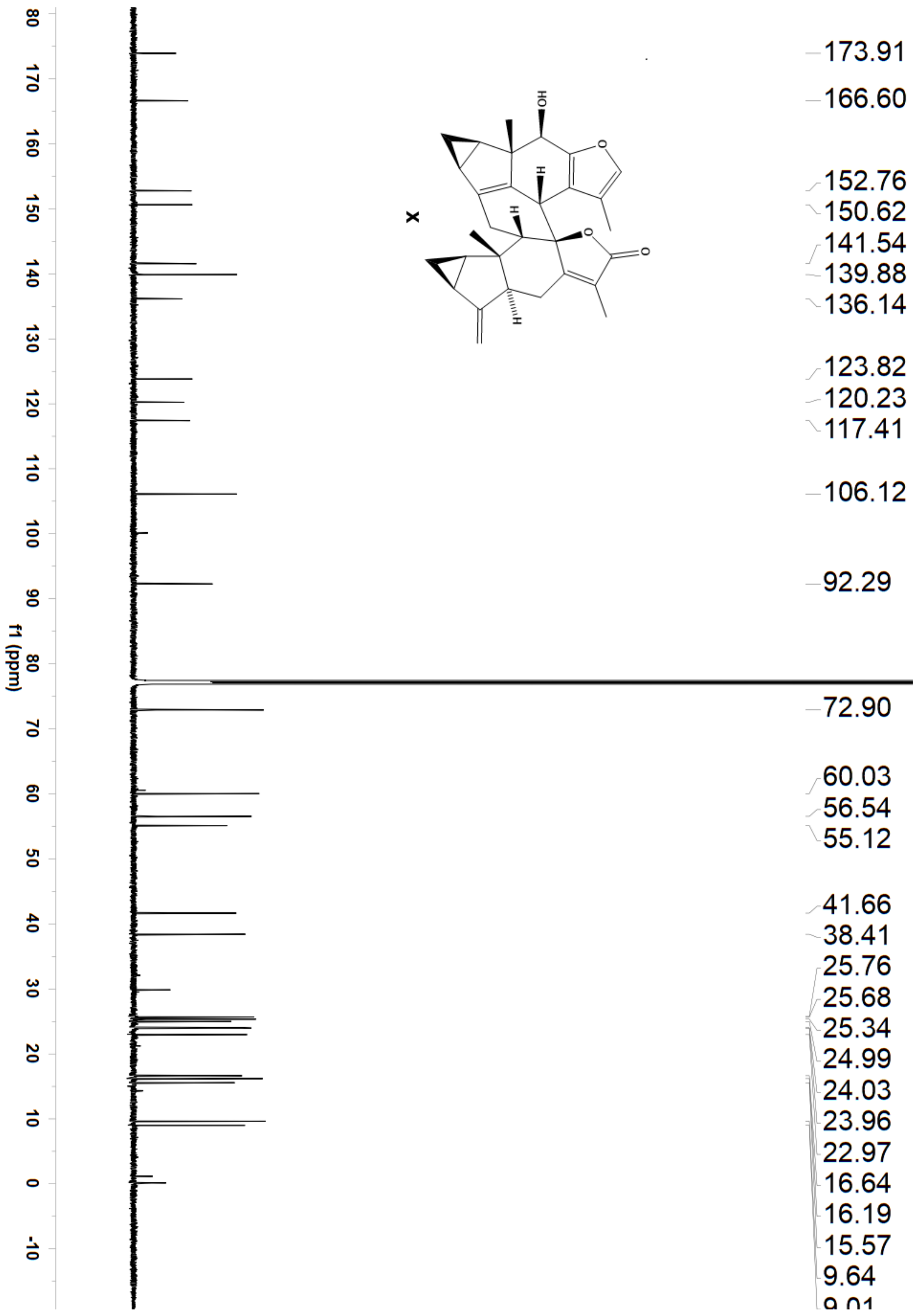

Supplementary Figure 89. ^{13}C NMR spectra of compound **x** in $CDCl_3$ (200 MHz)

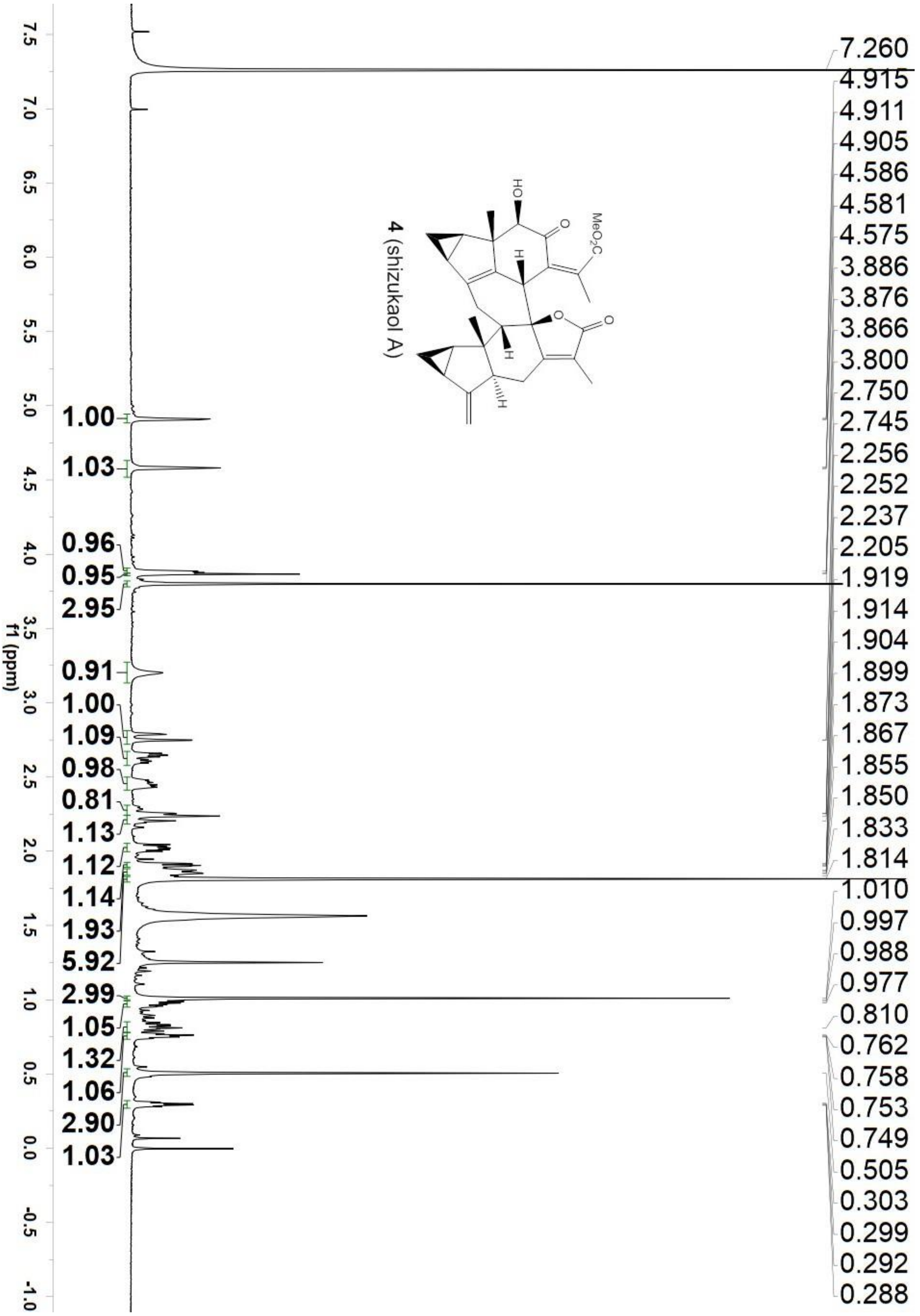

Supplementary Figure 90. ^{1}H NMR spectra of compound **4** in $CDCl_3$ (400 MHz)

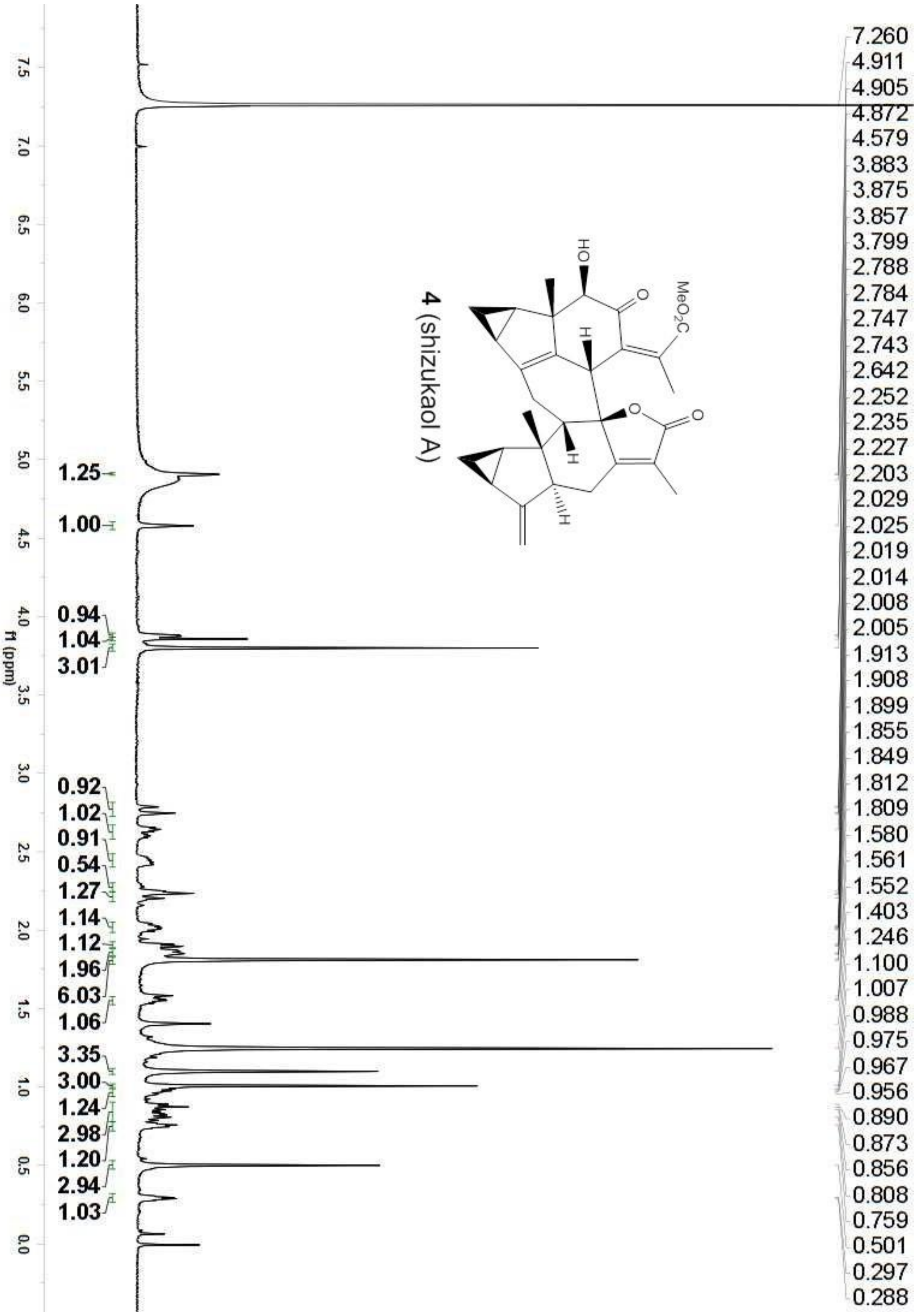

Supplementary Figure 91. ^{1}H NMR spectra of compound **4** in $CDCl_3$ with a drop of D_2O (400 MHz)

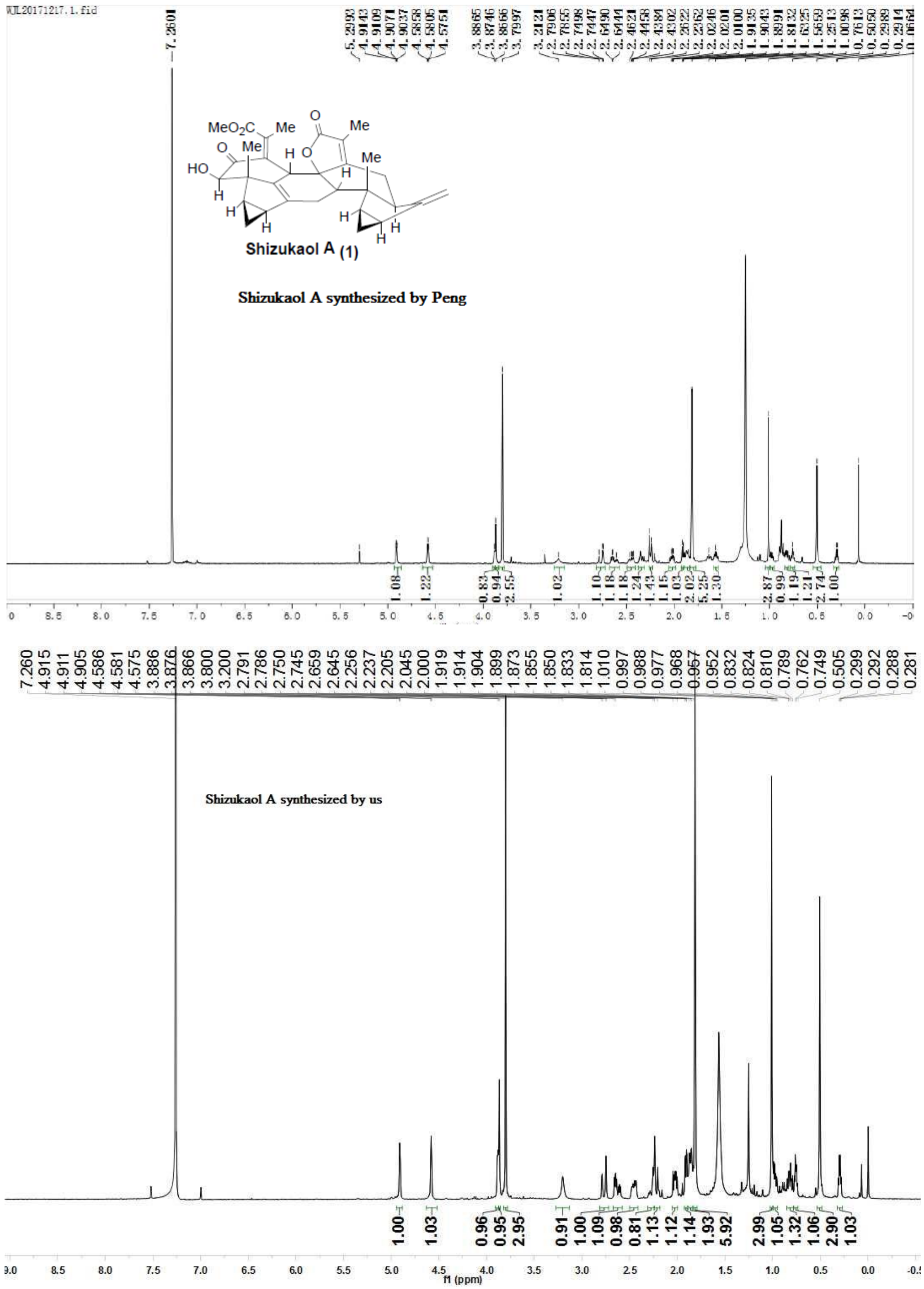

Supplementary Figure 92. Comparison of ^{1}H NMR data of Shizukaol A synthesized by Peng[13] (400 MHz) with those of Shizukaol A synthesized by us (400 MHz)

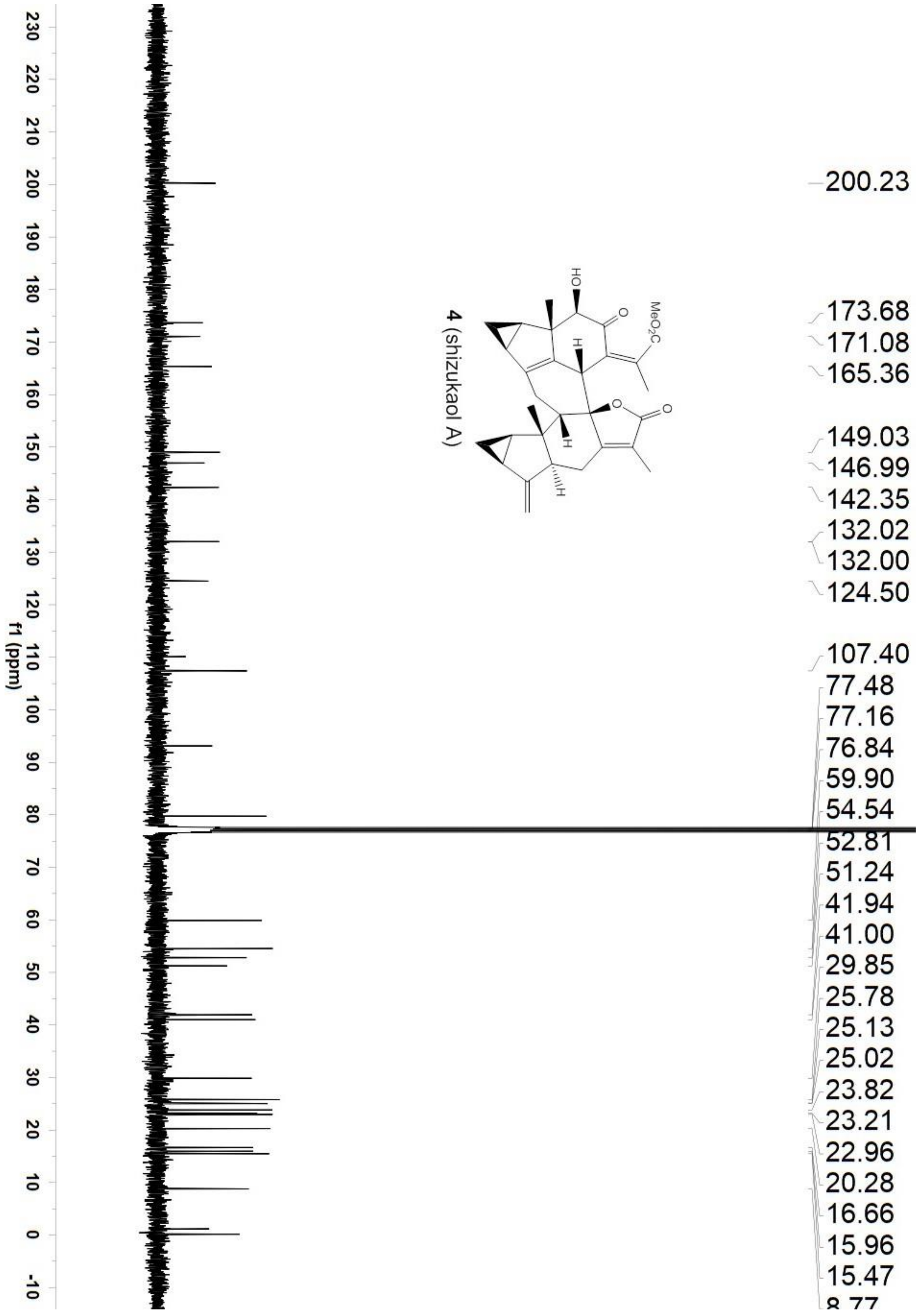

Supplementary Figure 93. ^{13}C NMR spectra of compound **4** in $CDCl_3$ (100 MHz)

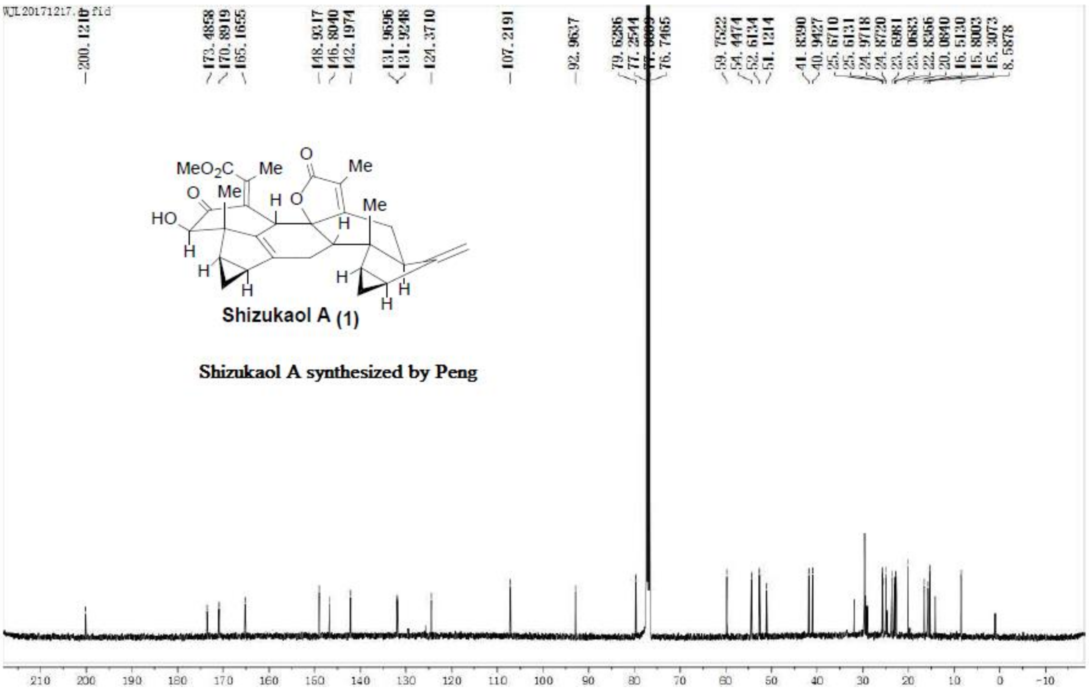

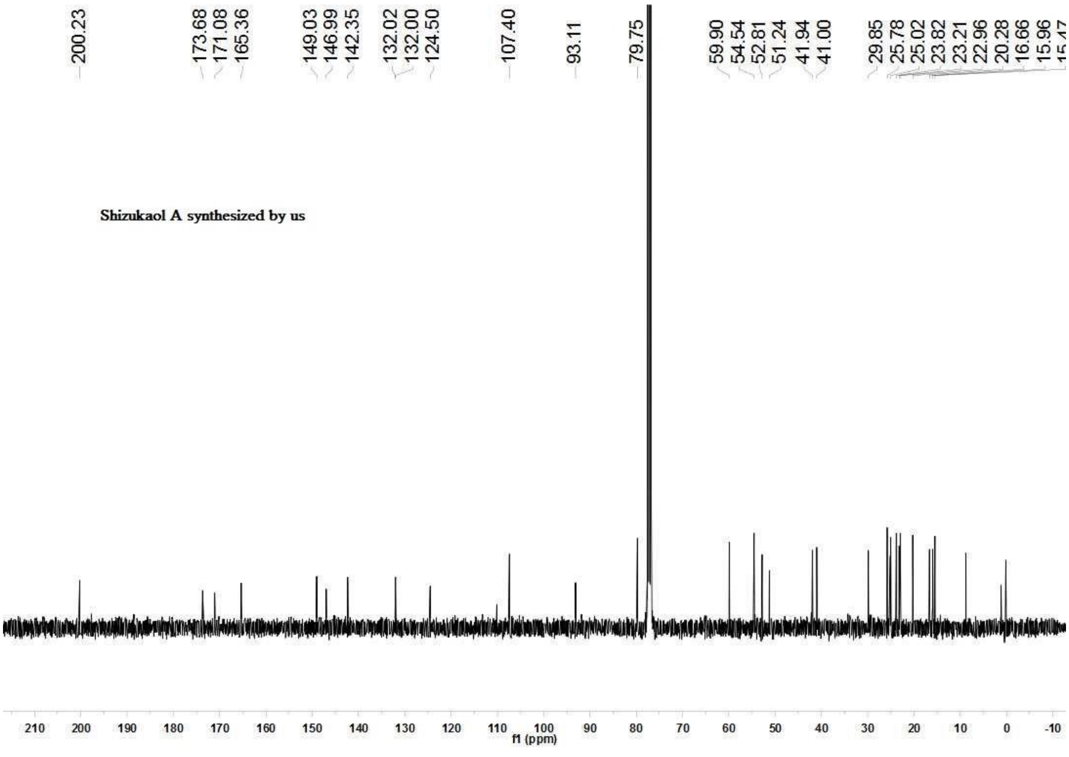

Supplementary Figure 94. Comparison of ^{13}C NMR data of Shizukaol A synthesized by Peng[13] (125 MHz) with those of Shizukaol A synthesized by us (100 MHz)

Supplementary References:

1. Yuan, C., Du, B., Yang, L. & B. Liu, Bioinspired Total Synthesis of Bolivianine: A Diels– Alder/Intramolecular Hetero-Diels–Alder Cascade Approach. *J. Am. Chem. Soc.* **135**, 9291-9294 (2013).
2. Yuan, C., Du, B., Deng, H., Man, Y. & Liu, B. Total Syntheses of Sarcandrolide J and Shizukaol D: Lindenane Sesquiterpenoid [4+2] Dimers. *Angew. Chem. Int. Ed.* **56**, 637-640 (2017).
3. Yang, Y., Li, J., Du, B., Yuan, C., Liu, B. & Qin, S. An entry to vinylcyclopropane through palladium-catalyzed intramolecular cyclopropanation of alkenes with unstabilized allylic tosylhydrazones. *Chem. Commun.* **51**, 6179-6182 (2015).
4. Su, C. & Williard, P.-G. Isomerization of Allyl Ethers Initiated by Lithium Diisopropylamide. *Org. Lett.* **12**, 5378-5381 (2010).
5. Wang, P., Luo, J., Zhang, Y.-M. & Kong, L.-Y. Sesquiterpene dimers esterified with diverse small organic acids from the seeds of *Sarcandra glabra*. *Tetrahedron* **71**, 5362-5370 (2015).
6. Zhang, S., Yang, S.-P., Yuan, T., Lin, B.-D., Wu, Y. & Yue, J.-M. Multistalides A and B, two novel sesquiterpenoid dimers from *Chloranthus multistachys*. *Tetrahedron Lett.* **51**, 764-766 (2010)
7. Kawabata, J. & Mizutani, J. Dimeric sesquiterpenoid esters from *Chloranthus serratus*. *Phytochemistry* **31**, 1293-1296 (1992).
8. Yang, Z.-J., Ge, W.-Z., Li, Q.-Y., Lu, Y., Gong, J.-M., Kuang, B.-J., Xi, X., Wu, H., Zhang, Q., Chen, Y. Syntheses and Biological Evaluation of Costunolide, Parthenolide, and Their Fluorinated Analogues. *J. Med. Chem.* **58**, 7007−7020 (2015).
9. Kawabata, J., Fukushi, E. & Mizutani, J. Sesquiterpene dimers from *Chloranthus japonicus*. *Phytochemistry* **39**, 121-125 (1995).
10. Fang, P.-L., Cao, Y.-L., Yan, H., Pan, L.-L., Liu, S.-C., Gang, N.-B., Lü, Y., Chen, C.-X., Zhong, H.-M., Guo, Y. & Liu, H.-Y. Lindenane Disesquiterpenoids with Anti-HIV-1 Activity from *Chloranthus japonicus*. *J. Nat. Prod.* **74**, 1408-1413 (2011).
11. Yue, G., Yang, L., Yuan, C., Jiang, X. & Liu, B. Total Synthesis of (±)-Chloranthalactone A. *Org. Lett.* **13**, 5406-5408 (2011).
12. Kawabata, J., Fukushi, Y., Tahara, S. & Mizutani, J. Shizukaol A, a sesquiterpene dimer from *Chloranthus japonicus*. *Phytochemistry* **29**, 2332-2334 (1990).

13 Wu, J.-L., Lu, Y.-S., Tang, B. & Peng, X.-S. Total syntheses of shizukaols A and E. *Nat. Commun.* **9**, 4040 (2018).
